# AT-hook DNA-binding motif-containing protein one knockdown downregulates EWS-FLI1 transcriptional activity in Ewing’s sarcoma cells

**DOI:** 10.1371/journal.pone.0269077

**Published:** 2022-10-04

**Authors:** Takao Kitagawa, Daiki Kobayashi, Byron Baron, Hajime Okita, Tatsuo Miyamoto, Rie Takai, Durga Paudel, Tohru Ohta, Yoichi Asaoka, Masayuki Tokunaga, Koji Nakagawa, Makoto Furutani-Seiki, Norie Araki, Yasuhiro Kuramitsu, Masanobu Kobayashi

**Affiliations:** 1 Advanced Research Promotion Center, Health Sciences University of Hokkaido, Kanazawa, Ishikari-Tobetsu, Hokkaido, Japan; 2 Department of Omics and Systems Biology, Graduate School of Medical and Dental Sciences, Niigata University, Niigata, Japan; 3 Department of Tumor Genetics and Biology, Faculty of Life Sciences, Kumamoto University, Kumamoto-Shi, Kumamoto, Japan; 4 Center for Molecular Medicine and Biobanking, University of Malta, Msida, Malta; 5 Division of Diagnostic Pathology, Keio University School of Medicine, Shinano, Shinjuku-ku, Tokyo, Japan; 6 Department of Molecular and Cellular Physiology, Yamaguchi University Graduate School of Medicine, Ube, Yamaguchi, Japan; 7 Department of Systems Biochemistry in Pathology and Regeneration, Yamaguchi University Graduate School of Medicine, Ube, Yamaguchi, Japan; 8 Department of Obstetrics and Gynecology, Yamaguchi University Graduate School of Medicine, Ube, Yamaguchi, Japan; Hirosaki University Graduate School of Medicine, JAPAN

## Abstract

Ewing’s sarcoma is the second most common bone malignancy in children or young adults and is caused by an oncogenic transcription factor by a chromosomal translocation between the EWSR1 gene and the ETS transcription factor family. However, the transcriptional mechanism of EWS-ETS fusion proteins is still unclear. To identify the transcriptional complexes of EWS-ETS fusion transcription factors, we applied a proximal labeling system called BioID in Ewing’s sarcoma cells. We identified AHDC1 as a proximal protein of EWS-ETS fusion proteins. AHDC1 knockdown showed a reduced cell growth and transcriptional activity of EWS-FLI1. AHDC1 knockdown also reduced BRD4 and BRG1 protein levels, both known as interacting proteins of EWS-FLI1. Our results suggest that AHDC1 supports cell growth through EWS-FLI1.

## Introduction

Ewing’s sarcoma is the second most common bone malignancy in children or young adults. This tumor is caused by a chromosomal translocation of the EWS RNA binding protein 1 (EWSR1) and the E-twenty-six (ETS) transcription factor family, which mainly consists of the Friend Leukemia integration 1 (FLI1), ETS-related gene (ERG), ETS translocation variant 4 (ETV4), or other kinds of ETS transcription factors [1, 2]. The EWS-FLI1 fusion protein, consisting of the EWSR1 gene and the FLI1 gene, which is caused by chromosomal translocation, is detected in more than 85% of cases in Ewing’s sarcoma.

Transcription factors have been undruggable because they do not have ligand-binding pockets that small molecules can recognize and do not have a folding structure [3]. Transcriptional complexes that interact with oncogenic transcription factors are promising targets. EWS-ETS fusion proteins need more co-operational transcription factors and co-transcriptional regulators for the oncogenic functions [4–6]. Several interacting partners of EWS-ETS fusion proteins have been isolated as druggable targets [1]. RNA helicase A interacts with EWS-FLI1, and their interaction is inhibited by a small molecule, YK-4-279, resulting in reduced tumor growth in vitro and in vivo [7]. PARP1 also interacts with EWS-FLI1, and PARP1 inhibitors inhibit tumor growth [8]. Recently, BRD4, one of the super-enhancers and a target of the BET inhibitor, also interacted with the EWS-ETS fusion protein and reduced tumor growth [9, 10]. Therefore, transcriptional complexes with the EWS-ETS fusion protein might be a druggable target.

The proximal protein biotinylation method has been developed to identify proximal complexes of the target proteins using the biotin identification (BioID) and the ascorbate peroxidase (APEX) method [11]. Roux *et al*. developed a BioID method that uses BirA mutant (R118G) to provide biotinyl-5’-AMP intermediate and induces nonspecific biotinylation of the proximal proteins [12]. EWS-FLI1 interactome analysis using the BioID method has already been achieved in human embryonic kidney 293T (HEK293T) cells. This approach showed that the cation-independent mannose 6-phosphate receptor works as a transporter of lysosomal hydrolases via lysosome-dependent turnover of EWS-FLI1 [13].

This study aims to identify new interacting proteins of EWS-ETS fusion proteins using the BioID system in Ewing’s sarcoma cells and investigate whether these affect cell growth and transcription of EWS-ETS fusion proteins. Our approach identified AT-hook DNA-binding motif-containing protein 1 (AHDC1) as one of the proximal proteins for EWS-ETS fusion proteins. AHDC1 has been revealed as a responsible gene in Xia-Gibbs syndrome patients, which causes an autosomal dominant multisystem developmental disorder [14–19]. AHDC1 knockdown showed reduced protein levels of EWS-FLI1 or target proteins of EWS-FLI1. AHDC1 knockdown also reduced the transcriptional level of NR0B1 that harbors the GGAA microsatellite region within the promoter region. In addition, AHDC1 knockdown showed reduced cell growth in Ewing’s sarcoma cell lines but not non-Ewing’s cells. Together, we suggest that AHDC1 is one of the transcriptional co-regulators of EWS-ETS fusion proteins in Ewing’s sarcoma cells.

## Materials and methods

### Cell culture

The A673 cell line was purchased from the European Collection of Authenticated Cell Cultures (ECACC) and cultured in Dulbecco’s Modified Eagle Medium (DMEM, Cat. No. 044–29765, Fujifilm-Wako chemical) supplemented with 10% heat-inactivated fetal bovine serum (FBS) and 1x Penicillin-Streptomycin Solution (Cat. No. 168–23191, Fujifilm-Wako chemical). The Seki cell line was established by Nojima *et al*. [20], purchased from the Cell Resource Center for Biomedical Research, Institute of Development, Aging and Cancer, Tohoku University (Cat. No. TKG 0725, Miyagi, Japan), and cultured in RPMI-1640 (Cat. No. 189–02025, Fujifilm-Wako chemical) with 10% FBS and 1x Penicillin-Streptomycin Solution. The NCR-EW2 cell line and SAOS-2 cell line (ATCC HTB-85) were cultured in RPMI-1640 with 10% FBS and 1x Penicillin-Streptomycin Solution. Human Embryonic Kidney cells 293 (HEK293) cells and hTERT RPE-1 (ATCC CRL-400), Lenti-X^™^ 293T cell line (Cat. No. 632180, Takara-bio), and U-2 OS cell line were cultured in DMEM with 10% FBS and 1x Penicillin-Streptomycin Solution. Seki, NCR-EW2, and Lenti-X293T cells were spread onto a 0.1% gelatin-coated dish.

### Plasmids

PrimeSTAR max polymerase (Cat. No. R045A, Takara-Bio) or KOD one polymerase (Cat. No. KMM-101, Toyobo) was used for precise cloning. The Welcome Sanger Institute kindly provided the pPB-LR5 [21] and pCMV-HyPBase [22] for the *piggyBac* system. The puromycin-resistant gene region, amplified from the linear puro marker (Cat. No. 631626, Takara-Bio), was inserted using the In-fusion HD cloning kit (Cat. No. 639648, Takara-bio) into the SpeI restriction site in the pPB-LR5, resulting in pPB-LR5-puro. The Tet3G-tet promoter-3xFLAG-EGFP fragment was amplified and inserted using the In-fusion HD cloning kit, resulting in the construction of pPBP-tet-3xEGFP. The BioID fragment was amplified from pcDNA3.1 mycBioID (Addgene: 35700) [12] and inserted into the pPBP-tet-3xEGFP after cutting at the KpnI and PmeI restriction enzyme sites, resulting in the construction of pPBP-tet-3xBioID-gs. The EWS-FLI1, EWS-ERG, and EWS-ETV4 genes were amplified from pcDNA3-EWS-FLI1typeI, EWS-ERG, EWS-ETV4 [23], and inserted into the PmeI restriction enzyme site of pPBP-tet-3xBioID-gs, resulting in pPBP-tet-3xBioID-EWS-FLI1, pPBP-tet-3xBioID-EWS-ERG, and pPBP-tet-3xEWS-ETV4, respectively. The AHDC1 gene (Genbank accession No. NM_001029882) was amplified from the cDNA of hTERT RPE-1 cells and inserted into the KpnI and PmeI restriction enzyme sites of pPBP-tet-3xEGFP, resulting in the construction of pPBP-tet-3xAHDC1. pGreenpuro shRNA cloning and expression lentivector was purchased from System Bioscience (Cat. No. SI505A-1, System Biosciences). Primers for shRNA are shown in S1 Table. For shAHDC1, shFLI1, and shEWS, each primer shAHDC1-f and shAHDC1-r, shFLI1-f and shFLI1-r, shEWS-f and shEWS-r were annealed and inserted into the EcoRI and BamHI restriction enzyme sites of the pGreenpuro shRNA cloning vector. For measuring the transcriptional activity of EWS-FLI1, the NR0B1 promoter region was cloned from A673 genomic DNA, which was purified using a QIAamp DNA Mini Kit (Cat. No. 51304, QIAGEN), and inserted into the XhoI restriction enzyme site of pNL1.1[Nluc] vector (Cat. No. N1001, Promega), resulting in the construction of pNL1.1-NR0B1pro vector.

### Lentivirus production and transduction

For shRNA-expressing lentivirus production, 5 × 10^6^ Lenti-X 293T cells were cultured in 10 ml of DMEM medium on a plate coated with 0.1% gelatin for 24 h. Seventeen μg of pGreenpuro shRNA-expressing vector, ten μg of pCAG-HIVgp (RDB04394, RIKEN BRC) [24], and 10 μg of pCMV-VSV-G-RSV-Rev (RDB04393, RIKEN BRC) [24] were mixed with 111 μl of 1 mg/ml PEI MAX^®^ (pH7.5) (Cat. No. 24765–1, Polysciences) in Opti-MEM^™^ I Reduced Serum Medium (Cat. No. 31985070, Thermofisher Scientific) for 10 min. After changing the medium, the DNA mixture was treated and incubated for 6–24 h. The next day, 100 μl of 500 mM sodium butyrate was added to enhance lentivirus production after changing the medium. The next day, 10 ml of medium were filtrated on 0.45 μm PVDF membrane of Millex-HV^®^ filter unit (Cat. No. SLHV R25 LS, MERCK KGaA), 3.5 ml of 4x PEG solution (32% PEG6000, 400 mM NaCl, 40 mM HEPES, adjusted to pH7.4) were added [25] for 1 h at 4°C and followed by centrifugation at 3000 rpm for 30 min at 4°C. The lentiviral pellet was mixed with 100 μl of Phosphate buffered saline (-) [PBS(-), 137 mM NaCl, 8.1mM Na_2_HPO_4_, 2.68 mM KCl, 1.47 mM KH_2_PO_4_, pH7.4] containing 2.5% glycerol and stored at -80°C. Cells were cultured in a 12-well or 6-well plate for one day. The medium was replaced with a medium containing lentivirus particles and five μg/ml of DEAE-dextran to enhance lentivirus production [26] and incubated for two days. The medium was again cultured one more day for further analysis.

### Knockdown of target genes

Cells were cultured in a 6-well plate for a day; 100 pmol of siRNA was mixed with 4 μl of Lipofectamine^™^ RNAiMAX Transfection Reagent (Cat. No. 13778030, Thermofisher scientific) in Opti-MEM^™^ I Reduced Serum Medium and incubated for 10 min, followed by transfer to each well. A Stealth RNAi^™^ siRNA Negative Control Med GC Duplex #2 (siNC, Cat. No. 12935112, Thermofisher Scientific) was used as negative control siRNA. The AHDC1 siAHDC1 used was a Stealth RNAi^™^ siRNA (siRNA ID: HSS146954, Thermofisher Scientific).

### Reverse transcription-quantitative PCR (RT-qPCR)

Total RNA was purified using the FastGene^™^ RNA basic kit (Cat. No. FG-80050, NIPPON Genetics). According to the procedure, cDNA was obtained using ReverTra Ace^®^ qPCR RT Master Mix with gDNA Remover (Cat. No. FSQ-301, Toyobo). qPCR was performed using Applied Biosystems^™^ PowerUp^™^ SYBR^™^ Green Master Mix (Cat. No. A25742, Thermofisher Scientific) with a StepOnePlus^™^ Real-Time PCR System (Thermofisher Scientific). The thermal cycling parameters followed PCR amplification conditions: 50°C for 2 min and 95°C for 2 min, 40 cycles of 95°C for 15 s, and 60°C for 1 min. The oligonucleotides used for RT-qPCR are shown in S2 Table. Relative quantification of each target was normalized by Glyceraldehyde-3-phosphate dehydrogenase (GAPDH). Error bars indicate the standard deviation of three independent biological replicates. Statistical analyses were performed by Student’s t-test.

### Western blot analysis

Cells were cultured and lysed in RIPA buffer [50 mM Tris-HCl pH8, 150 mM NaCl, 1% Nonidet P-40 (NP-40), 0.1% sodium dodecyl sulfate (SDS), 0.5% sodium deoxycholate, 10 μg/mL leupeptin, 10 μg/mL aprotinin, 1 mM Phenylmethylsulfonyl fluoride (PMSF), 1.5 mM Na_2_VO_4_, 10 mM NaF], sonicated for 10–15 s, and centrifugated at 15000 rpm for 15 min. Supernatants were used for the following procedure. According to the manufacturer protocol, protein concentration was determined by the Protein assay BCA kit (Cat. No. 297–73101, Fujifilm-Wako chemical). An equal amount of protein (10 μg) was applied in 5–20% SDS-polyacrylamide gel (SuperSep Ace; Cat. No. 199–15191, Fujifilm-Wako chemical) and transferred to the PVDF membrane (Immobilon-P; Millipore, Bedford, MA, USA). The membrane was blocked by 5% skimmed milk or 5% BSA for 1 h with shaking, incubated with a primary antibody at 4°C overnight, and a horseradish peroxidase (HRP)-conjugated secondary antibody for 1 h at room temperature with shaking. The membrane was visualized by Immunostar Zeta (Cat. No. 297–72403, Fujifilm-Wako chemical) and detected by using an Amersham Imager 600 (GE healthcare) or a WSE-6100 LuminoGraph I (ATTO Co., Ltd). Immunostaining for the PVDF membrane was performed using the following antibodies: FLI1 (1:1000 dilution, Cat. No. ab15289, Abcam), FLI1(EPR4646) (Cat. No. ab133485, Abcam), EWSR1 (1:2000 dilution, Cat. No. 11910S, Cell Signaling Technology), BRD4 (1:1000 dilution, Cat. No. AMAb90841, Sigma-Aldrich), DDDDK (1/2000 dilution, Cat No. PM020, MBL), DYKDDDDK (1:4000 dilution, Cat. No. 018–22381, Fujifilm-Wako chemical), NKX2-2 (1:1000 dilution, Cat. No. ab187375, Abcam), NR0B1 (1:2000 dilution, Cat. No. PA5-95912, Invitrogen), p27 Kip1 (D69C12) (1:2000 dilution, Cat. No. 3686, Cell Signaling Technology), GAPDH (D16H11) (1:5000 dilution, Cat. No. 5174, Cell Signaling Technology), BRG1 (A52) (1:2000 dilution, Cat. No. 3508, Cell Signaling Technology), AHDC1 (1:1000 dilution, Cat. No. HPA028648, Atlas antibodies), SOX2 (1:2000 dilution, Cat. No. GTX627405, GeneTex), NUDT21 (1:100 dilution, Cat. No. sc-81109, Santa Cruz Biotechnology), CPSF6 (1:2000 dilution, Cat. No. 75168, Cell Signaling Technology), CPSF7 (1:100 dilution, Cat. No. sc-393880, Santa cruz), RBM33 (1:1000 dilution, Cat. No. A303-928A, Bethyl laboratories), c-Jun (60A8) (1:1000 dilution, Cat. No. 9165, Cell Signaling Technology), JunB (C37F9) (1:1000 dilution, Cat. No. 3753, Cell Signaling Technology), JunD (D17G2) (1:1000 dilution, Cat. No. 5000, Cell Signaling Technology), c-Fos(9F6) (1:1000 dilution, Cat. No. 2250, Cell Signaling Technology), FRA1 (D80B4) (1:1000 dilution, Cat. No. 5281, Cell Signaling Technology), FRA2 (D2F1E) (1:1000 dilution, Cat. No. 19967, Cell Signaling Technology), CREB5 (1:1000 dilution, Cat. No. 14196-ap, Proteintech) TdTIF1 (1:100 dilution, Cat. No. sc-166296, Santa Cruz Biotechnology), ARNT (1:100 dilution, Cat. No. sc-55526, Santa Cruz Biotechnology), RBM26 (1:1000, Cat. No. HPA040252, Sigma-Aldrich), IRX4 (1:1000 dilution, Cat. No. AV32066, Sigma-Aldrich), beta-actin (AC-15) (1:2000 dilution, Cat. No. A1978, Sigma-Aldrich), SNAIL (C15D3) (1:1000 dilution, Cat. No. 3879, Cell Signaling Technology), α-SMA (1A4) (1:2000 dilution, Cat. No. ab7817, Abcam). For inhibition of protein synthesis or proteasome, we treated with 20 μg/ml Cycloheximide (Cat. No. sc-3508B, Santa Cruz Biotechnology) / dimethyl sulfoxide (DMSO) or 10 μM (S)-MG-132 (Cat. No. 10012628, Cyman chemical) / DMSO in siRNA-treated cells.

### Immunostaining

Cells were cultured and fixed using 4% Paraformaldehyde/PBS(-) for 15 min, permeabilized using 0.1% Triton X-100/PBS(-) for 15 min, and blocked using 1% goat serum (Cat. No. 50062Z, Life technologies) for 15 min. Cells were incubated with primary antibodies at 4°C overnight and stained with secondary antibodies and five μg/ml 4’,6-Diamidino-2-phenylindole, dihydrochloride (DAPI). Primary antibodies used were DDDDK (1:1000 dilution, Cat. No. PM020, MBL), DYKDDDDK (1:2000 dilution, Fujifilm-Wako pure chemical), BRD4 (1:200 dilution, Sigma-Aldrich), or BRG1 (1:50 dilution, CST). Secondary antibodies used were Alexa Fluor 488-conjugated Goat anti-Mouse IgG (1:500 dilution, Cat. No. A-11001, Thermofisher Scientific), Alexa Fluor 488-conjugated Goat anti-Rabbit IgG (1:500 dilution, Cat. No. A-11034, Thermofisher Scientific), Alexa Fluor 555-conjugated Goat anti-Mouse IgG (1:500 dilution, Cat. No. A-21422, Thermofisher scientific), or Alexa Fluor 555-conjugated Goat anti-Rabbit IgG (1:500 dilution, Cat. No. A-21428, Thermofisher Scientific). SlowFade^™^ diamond antifade mountant (Cat. No. S36963, Thermofisher Scientific) was used as a mounting reagent. Nikon A1R HD25 system confocal microscope with ECLIPSE Ti2E (Nikon) was used for the observation.

### Chromatin immunoprecipitation (ChIP) assay

Cells that induced FLAG-tagged proteins by 1 μg/ml doxycycline in a 10-cm dish for 1 d were washed by PBS(-), treated with 275 μl of 37% formaldehyde at 1% final concentration in the medium for 10 min, and followed by addition of 1 ml of 1.25 M glycine for 5 min. Cells were washed again with PBS(-) and collected in PBS(-) by centrifugation at 2000 rpm for 5 min. The cell pellet was lysed by 300 μl of ChIP lysis buffer [1% TritonX-100, 0.1% sodium deoxycholate, 0.1% SDS, 2 mM EDTA, 150 mM NaCl, 50 mM Tris (pH8)] for 15 min and sonicated with 40% amplitude of a sonicator 250 (Branson) for 30 s twice, and centrifuged for 10 min at 12000 rpm. Two hundred fifty μl of the supernatant was mixed with 250 μl of RIPA buffer, and 50 μl was divided as a 10% Input. The remaining mixture was rotated with 50 μl of anti-DDDDK-tag mAb magnetic beads (M185-11, MBL) for 3 h at 4°C. Beads were washed by 1 ml of low salt buffer [1% TritonX-100, 0.1% SDS, 2mM EDTA, 150 mM NaCl, 50 mM Tris-HCl(pH8)] twice, high salt buffer [1% TritonX-100, 0.1% SDS, 2mM EDTA, 500 mM NaCl, 50 mM Tris-HCl(pH8)] twice, LiCl buffer [0.25 M LiCl, 1% NP-40, 1% sodium deoxycholate, 1 mM EDTA, 50 mM Tris-HCl(pH8)] twice, and TE buffer [1 mM EDTA, 10 mM Tris-HCl(pH8)] twice. For reverse crosslinking, beads were treated with 120 μl of elution buffer (1% SDS, 100 mM NaHCO_3_) for 15 min on a rotator twice. The supernatant from the beads was added to 9.6 μl of 5 M NaCl and 2 μl of 10 mg/ml RNase A at 65°C overnight, followed by the addition of 2 μl of protein K for 1 h at 60°C with 10% Input samples. The DNA from the supernatant was cleaned by Fastgene^™^ PCR/Gel extraction kit (FG-91302, NIPPON genetics). The polymerase chain reaction was performed by KOD one PCR master mix (KMM-101, Toyobo) according to the procedure with NR0B1-ChIP-f (5’-tgaaatttaacgctgcaagcaaaatgg-3’) and NR0B1-ChIP-r (5’-ccgcagtgagaaattttgatccttgt-3’) primers at 35 cycles.

### Biotin labeling in living cells and elution of the biotinylated proteins

Cells were induced to produce BioID fusion proteins by 1 μg/ml of doxycycline with 50 μM biotin for 24 h in a 10-cm dish. Isolation of the biotinylated proteins was followed by the Couzens *et al*. method [27]. After washing the cells with PBS 3 times, cells were lysed by 500 μl of RIPA buffer (50 mM Tris-HCl pH8, 150 mM NaCl, 1% NP-40, 0.1% SDS, 0.5% sodium deoxycholate, 1 mM EDTA, 1mM EGTA, 10 μg/ml leupeptin, 10 μg/ml aprotinin, 1 mM PMSF, 1.5 mM Na_2_VO_4_, 10 mM NaF). The cell lysate was incubated with 1 μl of Benzonase (Cat. No. 70746-3CN, Millipore), shaken on an icebox for 1 h, then sonicated for 15 s, and centrifuged at 15000 rpm for 15 min. The supernatant was mixed with 50 μl of streptavidin sepharose (Cat. No. 17-5113-01, GE healthcare), shaken at 4°C for 3 h after being washed with PBS(-) once. After collecting beads by centrifugation, the beads were washed with RIPA buffer without protease inhibitors once, washed with TAP buffer (50 mM HEPES-KOH pH 8.0, 100 mM KCl, 10% glycerol, 2 mM EDTA, 0.1% NP-40) twice, and washed with 50 mM ammonium bicarbonate (pH 8.0) six times. The beads were incubated with 100 μl of 50 mM Tris-HCl (pH8.5) and 1 μl of 5 μg/μl dithiothreitol (DTT) with shaking at 37°C for 1 h. In addition, 1 μl of 12.5 mg/mL iodoacetamide was added to the beads and incubated with shaking at 37°C for 1h in the dark. The beads were added with 2.5 μl of 200 ng/μl Trypsin/Lys-C Mix (Cat. No. V5073, Promega) at 37°C with shaking overnight. The supernatant was collected by centrifuge, collected again after the beads were washed with 50 mM Tris-HCl (pH8.5), and added with 10 μl of 20% trifluoroacetic acid (TFA).

For the desalting step, styrene-divinylbenzene (SDB)-stage tip was washed with 20 μL of 0.1% TFA in 80% acetonitrile and further washed SDB-stage tip with 20 μl of 0.1% TFA in 2% acetonitrile. The peptide digest was transferred to the SDB-stage tip and trapped by centrifugation. The SDB-stage tip was washed with 20 μl of 0.1% TFA in 2% acetonitrile and 0.1% TFA in 80% acetonitrile. The peptides were eluted with 200 μl of 0.1% TFA, and 1–2 μl of peptide solution was applied for mass spec analysis.

### Liquid chromatograph–mass spectrometry (LC-MS) analysis and label-free quantification

For BioID analysis, the peptide samples were subjected to a nano-flow reversed-phase (RP) LC-MS/MS system (EASY-nLC^™^ 1200 System coupled to an Orbitrap Fusion Tribrid Mass Spectrometer: Thermo Fisher Scientific, San Jose, CA) with a nanospray ion source in positive mode. Samples were loaded onto a 75-μm internal diameter × 2-cm length RP C18 precolumn (Thermo Scientific Dionex) and washed with loading solvent before switching the trap column in line with the separation column, a nano-HPLC C18 capillary column (0.075 × 125 mm, 3 mm) (Nikkyo Technos, Tokyo, Japan). A 60-min gradient with solvent B (0.1% Formic acids in 80% acetonitrile) of 5–40% for separation on the RP column equilibrated with solvent A (0.1% formic acid in water) was used at a flow rate of 300 nl/min. MS and MS/MS scan properties were as follows; Orbitrap MS resolution 120,000, MS scan range 350–1500, isolation window m/z 1.6, and MS/MS detection type was ion trap with a rapid scan rate.

All MS/MS spectral data were searched against entries for human in the Swiss-Prot database (v2017-06-07) with a mutant form of *E*. *coli* biotin ligase (BirA) using the SEQUEST database search program using Proteome Discoverer 2.2 (PD2.2). The peptide and fragment mass tolerances were set to 10 ppm and 0.6 Da, respectively. For variable peptide modifications, oxidation of methionine and biotinylation of lysine, in addition to carbamidomethylation of cysteine for a fixed modification, were considered. Database search results were filtered by setting the peptide confidence value as high (FDR < 1%) for data-dependent mass analysis data. For label-free quantification, the peptide and protein amount were calculated from the precursor ion intensities using the workflow of Precursor Ions Quantifier in PD2.2. The amount of mutant form of BirA quantified in each analysis was used for the bait normalization, and data from three independent replicas were averaged. ANOVA was performed using the same workflow to calculate the adjusted *P* values to control experiments (BirA and BirA-Luc2).

### Immunoprecipitation

Immunoprecipitation was performed with a slight modification of the following procedure [28]. Cells expressing 3xFLAG-tagged EGFP, ADHC1, or EWS-FLI1 under the control of a Tet-on system which was cultured in a medium containing 1 μg/ml doxycycline for 1 d, were washed by PBS(-) 3 times, collected in PBS(-) after scraping, and centrifuged at 450 *g* for 10 min at 4°C. The pellets were treated with 1 ml of hypotonic lysis buffer (10 mM KCl, 10 mM Tris pH 7.5, 1.5 mM MgCl_2_) supplemented with 1 mM DTT, 1 mM PMSF, 10 μg/ml Leupeptin, and 10 μg/ml Aprotinin for 15 min on ice followed by centrifugation at 400 *g* for 5 min at 4°C. Pellets were treated with 500 μl of hypotonic lysis buffer again and mixed by pipetting 10 times, followed by centrifugation at 10000 *g* for 20 min at 4°C. Pellets were treated with high-salt extraction buffer (0.42 M KCl, 10 mM Tris pH 7.5, 0.1 mM EDTA, 10% glycerol supplemented with 1 mM PMSF, 10 μg/ml Leupeptin, and 10 μg/ml Aprotinin) with 1 μl Benzonase (70746, Millipore), and gently shaken on an icebox for 30 min, followed by centrifugation at 20000 *g* for 5 min at 4°C. Supernatants were diluted by Milli-Q water to adjust to 150 mM salt concentration. 300 μg of nuclear lysate were topped up to 500 μl using IP wash buffer (150 mM KCl, 10 mM Tris pH 7.5, 0.1 mM EDTA, 10% glycerol) supplemented with 1 mM PMSF, 10 μg/ml Leupeptin, and 10 μg/ml Aprotinin. Fifty μl of Anti-FLAG M2 magnetic beads (Cat. No. M8823, Sigma Aldrich) or DDDDK magnetic beads (M185-11, MBL) were washed by PBS(-) once and rotated in 1 ml of 5% BSA/PBS(-) 1 h at 4°C. The nuclear lysate was mixed and rotated with anti-FLAG magnetic beads for 3 h at 4°C and washed using 1 ml of IP wash buffer 4 times. Beads were mixed with 50 μl of 2x SDS sample buffer at 95°C for 5 min. The supernatants were used for Western blotting analysis.

The nuclear lysate was collected using the above method for endogenous protein immunoprecipitation. 500 μg of nuclear lysate was topped up to 500 μl using IP wash buffer and mixed with 10 μg of FLI1 [EPR4646] antibody (ab133485, Abcam) or rabbit normal IgG (Cat.148-09551, Wako pure chemical), followed by rotation at 4°C for 2 h. The nuclear lysate/IgG was mixed with 25 μl of Pierce^™^ Protein A/G Magnetic Beads (Cat. 88802, Thermofisher Scientific) with rotation at 4°C for 2h. The beads were washed with IP wash buffer 4 times and mixed with 50 μl of 2x SDS sample buffer at 95°C for 5 min. The supernatants were used for Western blotting analysis.

### Cell viability assay

Lentiviral-transduced cells were collected without a drug selection, and 1 × 10^3^ cells were spread in a 96-well plate. An equal volume of CellTiter-Glo^®^ 2.0 reagent (Cat. No. G924B, Promega) was transferred into each well and incubated for 5 min. After pipetting each well, the mixture was transferred into a 1.5-ml tube, mixed by a shaker for 10 min at room temperature, and luminescence was measured by a GloMax^®^ 20/20 Luminometer (Cat. No. E5311, Promega). For measuring measure apoptotic activity, an equal volume of Caspase-Glo^®^ 3/7 Assay System (Cat. No. G8090, Promega) was transferred into each well and incubated for 1 h, and measured by a GloMax^®^ 20/20 Luminometer. For the spheroid formation assay, lentiviral-transduced cells were collected, and 1 × 10^4^ cells were spread in a PrimeSurface96U (Cat. No. MS-9096U, Sumitomo Bakelite). The medium was changed every 2 d, and photos were taken by an All-in-One fluorescence microscope BZ-810X (Keyence).

### Scratch wound healing assay

Lentiviral-transduced cells were cultured in a 12-well plate. The cell layer was scratched by a 1-ml tip, washed with PBS(-) twice, the medium was replaced with DMEM without FBS, and photos were taken by the BZ-810X.

### Promoter reporter assay

1 × 10^4^ cells were cultured in a 96-well plate. The next day, 50 ng of the pNL1.1-NR0B1 vector was transfected with 0.1 μl of Lipofectamine^™^ Stem Transfection Reagent (Cat. No. STEM00003, Thermofisher Scientific) according to the procedure and incubated for 4 h. Three pmol of siNC or siAHDC1 stealth siRNA was incubated with 0.125 μl of Lipofectamine^™^ RNAiMAX Transfection Reagent (Cat. No. 13778030, Thermofisher scientific) in Opti-MEM^™^ I Reduced Serum Medium for 10 min and treated in each well. After 2 d, an equal volume of Nono-Glo Live-cell assay system (Cat. No. N2011, Promega) was added to each well and mixed by pipetting and shaking for 5 min and measured by a GloMax^®^ 20/20 Luminometer. Luminescence of no-transfected cells was subtracted from each sample. Error bars show the standard deviation of five independent biological replicates. Statistical analyses were performed by Student’s t-test.

## Results

### Biotinylation of proximal proteins by BioID in A673 cells

**Fig 1:**
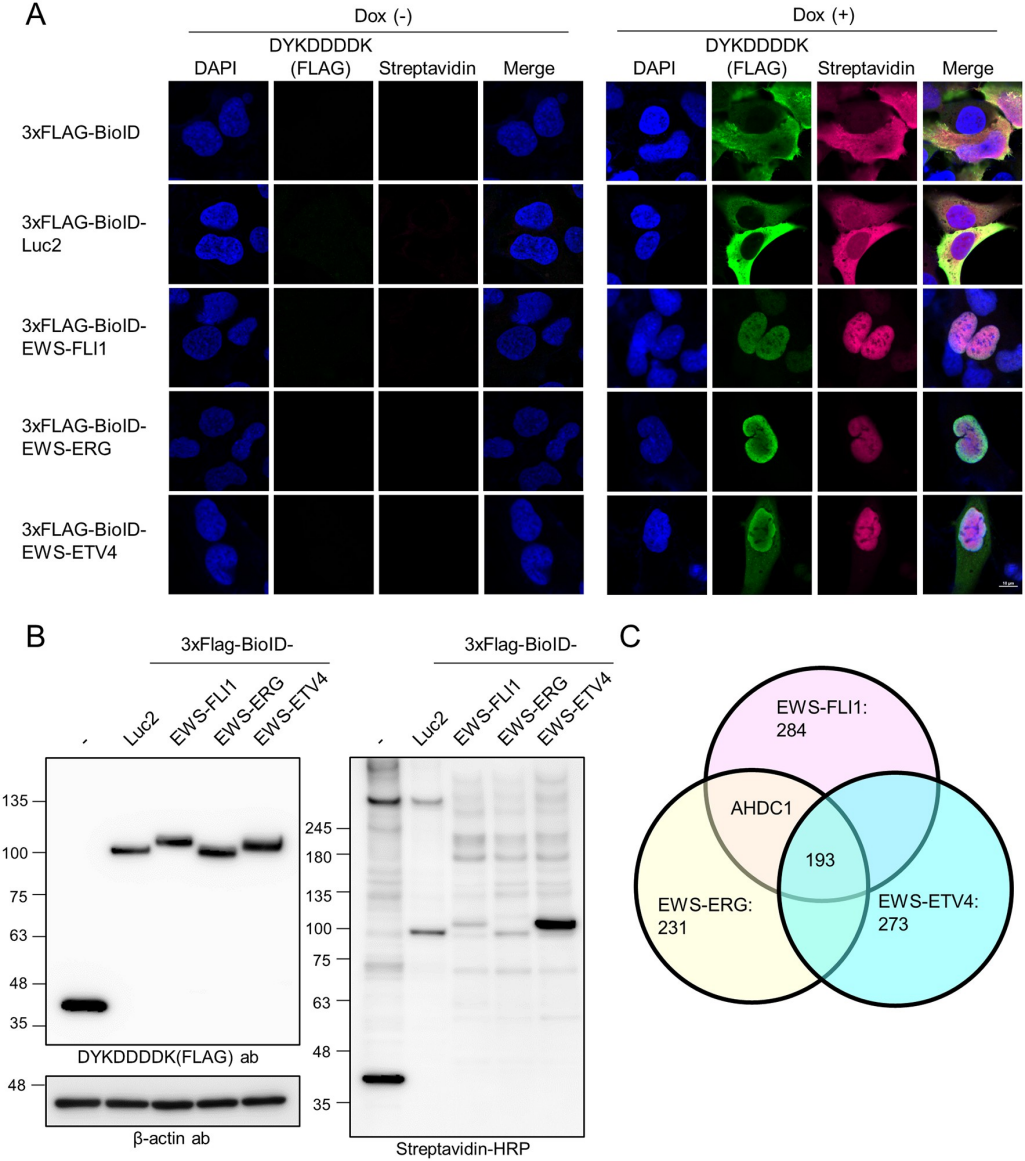
Identification of AHDC1 as a proximal protein of EWS-ETS fusion proteins. (A) 3xFLAG-BioID-tagged EGFP or EWS-ETS fusion proteins under the control of Tet-on promoter were expressed in A673 cells by 1 μg/ml doxycycline or absence for 1 d. FLAG-tag or biotinylated proteins were stained with DYKDDDDK antibody or Alexafluor633-conjugated streptavidin, respectively, with DAPI. (B) Western blotting analysis of each BioID sample. FLAG-tag was stained with DYKDDDDK antibody. Biotinylated proteins were stained with streptavidin-HRP, and β-actin was stained as an internal control. (C) Identified protein numbers from each EWS-ETS fusion protein sample by mass spectrometry analysis.

For BioID-tagged EWS-ETS fusion protein expression, we constructed the *piggyBac* system under the control of the Tet-on system to regulate the gene expression. BioID-tagged EWS-FLI1, EWS-ERG, or EWS-ETV4-expressing plasmids were transfected into A673 cells with a hyperactive *piggyBac* transposase previously generated for applications in mammalian genetics [22]. After a puromycin selection, cells expressed each BioID-tagged gene by doxycycline with biotin. BioID alone or BioID-tagged Luc2 (firefly luciferase) were used as a negative control and labeled biotin to proximal proteins in all cell fractions (Fig 1A). In addition, BioID-tagged EWS-ETS fusion proteins were mainly localized in the nuclei. Next, we checked whether BioID-tagged EWS-ETS fusion proteins could biotinylate proximal proteins in A673 cells by Western blotting (Fig 1B). Streptavidin-HRP staining confirmed the appearance of various biotinylation bands. BioID-tagged EWS-ETS fusion proteins were highly detected compared to endogenous EWS-FLI1 in a medium containing 1 μg/ml doxycycline (S1A Fig). NKX2-2 and NR0B1 downregulated in cells expressing BioID-tagged EWS-ETS fusion proteins. In addition, BioID-tagged EWS-ETS fusion protein expression reduced cell growth (S1B Fig).

We prepared three independent biological replicates for each cell line, collected biotinylated proteins by a streptavidin sepharose set up using the Couzens *et al*. method [27], and identified proteins by mass spectrometry analysis (Fig 1C and S3 Table). The negative controls were used BioID or BioID-tagged Luc2. For label-free quantification, peptide and protein amount were calculated from precursor ions (Materials and methods) and normalized using BirA(R118G) in each analysis. Each abundance intensity was calculated for each ratio using both BioID and BioID-Luc2. Out of 3879 proteins detected in all samples, 193 proteins were identified as proximal proteins shared from the three fusion proteins (Abundance ratio: each fusion proteins list compared to BioID and BioID-Luc2 > 5, Abundance Ratio Adj. p-value < 0.05). The STRING database visualized the common list of protein-protein networks, and 6 clusters were divided by k-means clustering (S2 Fig and S4 Table) [29]. Cluster 1 was contained polyadenylation specificity factors complex-related proteins [CPSF1, CPSF2, CPSF4, CPSF5 (NUDT21), CPSF6, CPSF7, and CSTF2]. Cluster 2 contained transcription factors-related proteins (CREB5, FOS, FOSL1, FOSL2, JUN, JUNB, JUND, TCFL2, and TEAD1). Cluster 3 contained splicing factor-related proteins (SF3A1, SF3A2, SF3A3, SF3B2, SF3B4, SNRNP70, SNRPA, SNRPC). Cluster 4 contained a DREAM MuvB core complex component (LIN37, LIN52, and LIN9). Cluster 5 contained mediator complex-related proteins (Cyclin-C, Cyclin-T2, MED11, MED13L, MED22, MED25, and MED30). Cluster 6 contained chromatin remodeling complex-related proteins [ARID1A, ARID2, SMARCA4 (BRG1), DPF1, PBRM1, PHF10, SMARCAL1, SMARCB1, SMARCC1, SMARCD1, SMARCE1, and SS18L1]. We checked the reproducibility of candidates in the common list by western blotting using several antibodies (S3 Fig). CPSF5, CPSF6, CPSF7, RBM33, c-JUN, JUNB, JUND, c-FOS, FOSL1, FOSL2, CREB5, and DNTTIP1 were confirmed by pulldown experiments using streptavidin beads, but ARNT, RBM26, and IRX4 did not confirm reproducibility.

Previous proximal proteins screening of EWS-FLI1 in 293 cells identified 366 proteins after subtracting false positive data [13]. We compared 193 proteins that were identified in our screening, resulting in overlaps of 21 proteins (ATF1, ATF7IP, ARID1A, BICRA, CPSF7, CREB5, CSTF2, DNTTIP1, ESS2, JUN, JUN-B, NKX2-5, RBM33, SMARCC1, TCF7L2, PRPF40A, SF3B4, SMARCE1, SNRPA, SNRPC, and SUMO2).

To characterize between EWS-ETS fusion proteins and an uncharacterized protein, we first focused on AHDC1 protein. AHDC1 was contained in the BioID-tagged EWS-FLI1 and EWS-ERG protein samples (Fig 1C). However, AHDC1 did not significantly differ in the BioID-tagged EWS-ETV4 protein list.

**Fig 2:**
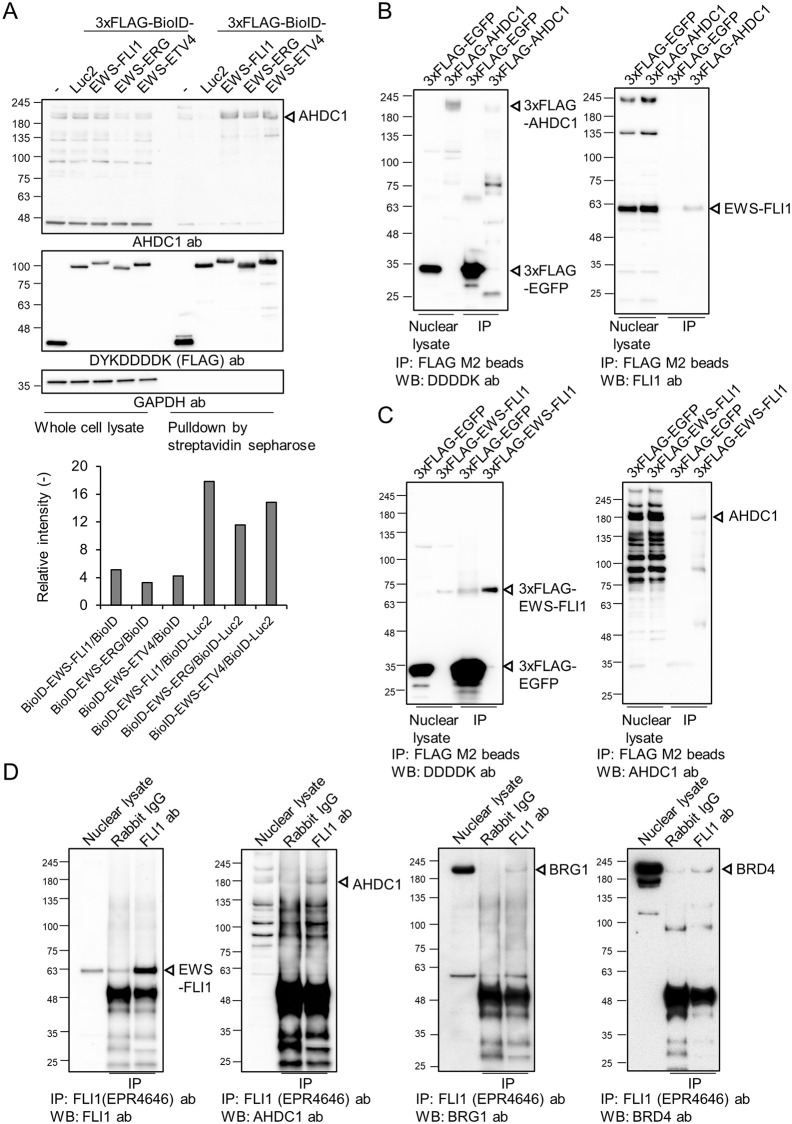
Immunoprecipitation of AHDC1. (A) Western blotting analysis after streptavidin-conjugated sepharose beads. Ten μg of proteins and one-tenth of pulldown input were used as a whole-cell lysate and a biotinylated protein sample, respectively. Band intensity was compared as a BioID or BioID-tagged Luc2. GAPDH antibody was used as a negative control. (B) Western blotting analysis of co-immunoprecipitated samples. 300 μg of nuclear lysate was mixed with FLAG M2 magnetic beads for immunoprecipitation. Five μg of nuclear and one-fifth of the immunoprecipitation input were used for western blotting. (C) Western blotting analysis of co-immunoprecipitated samples. 300 μg of nuclear lysate was mixed with FLAG M2 magnetic beads for immunoprecipitation. (D) Western blotting analysis of co-immunoprecipitated samples. 500 μg of nuclear lysate was combined with FLI1 antibody and protein A/G magnetic beads for immunoprecipitation. Five μg of nuclear lysate and one-fifth of the immunoprecipitation input were used for western blotting.

To determine whether AHDC1 is a proximal protein of EWS-ETS fusion proteins, we purified the biotinylated proteins again and detected AHDC1 (S4A Fig and Fig 2A). The intensity of AHDC1 in the EWS-ETS protein sample was higher than in each BioID and BioID-Luc2 sample. Next, immunoprecipitation for AHDC1 was performed using FLAG-tagged AHDC1-expressing cells (Fig 2B). FLAG-tagged AHDC1 was immuno-precipitated with endogenous EWS-FLI1 protein compared to FLAG-tagged EGFP. This result was reproduced using different magnetic beads (S4B Fig). FLAG-tagged EWS-FLI1 was also immunoprecipitated with endogenous AHDC1 compared to FLAG-tagged EGFP (Fig 2C). Moreover, endogenous EWS-FLI1 immunoprecipitants were included in AHDC1 with BRD4 and BRG1 (Fig 2D).

### AHDC1 knockdown affects gene expression of EWS-FLI1 target genes

**Fig 3:**
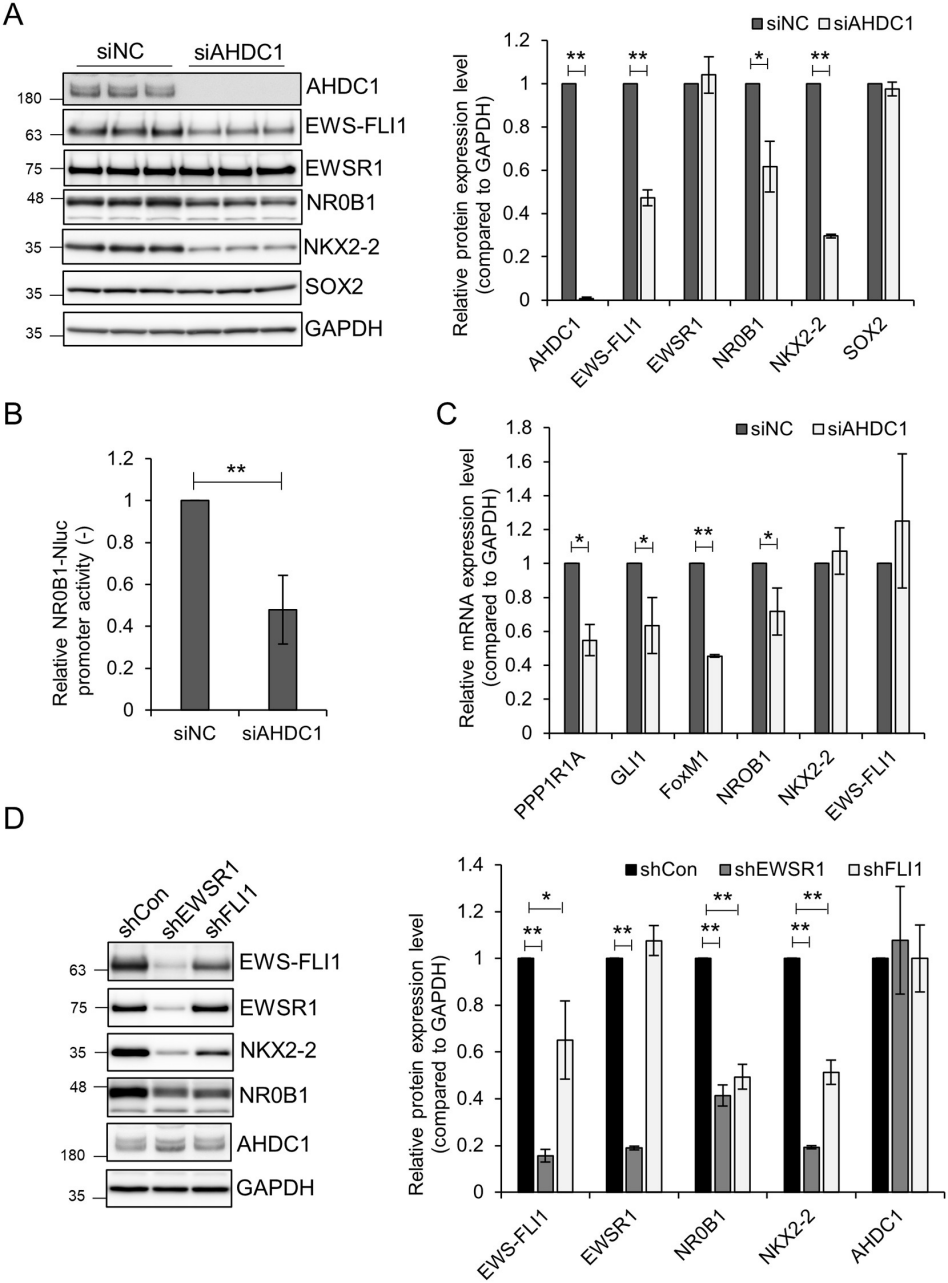
AHDC1 knockdown reduces gene expression of EWS-FLI1 protein. (A) siAHDC1-treated A673 cells were cultured for 2 d. Each protein was detected by its respective antibody. (B) NR0B1 promoter-Nluc plasmid was transfected into A673 cells, incubated for 4 h, and treated with siRNA for 2 d. The Nluc activity was measured by a Nono-Glo Live-cell assay system (C) siAHDC1-treated A673 cells were collected, total RNA was purified, and reverse-transcribed to cDNA. Each gene was quantified by the respective primer set using RT-qPCR. (D) Lentivirus expressing each shRNA was transduced into A673 cells for 3 d. GAPDH was used to normalize the relative values of western blotting and RT-qPCR. Three independent experiments quantified western blotting or RT-qPCR. Five independent experiments quantified the Nluc assay. *P* values were calculated by the student’s t-test. * p<0.05; ** p<0.001.

To evaluate whether AHDC1 affects gene expression of EWS-FLI1, we treated A673 cells with siRNA for the AHDC1 knockdown experiment. AHDC1 knockdown showed reduced EWS-FLI1 protein expression level but not EWSR1 (Fig 3A). The nuclear receptor NR0B1 and the homeobox transcription factor NKX2-2 were up-regulated in Ewing’s sarcoma [30–32]. NR0B1 and NKX2-2 protein expression levels were reduced in siAHDC1-treated cells. Silencing of EWS-FLI1-bound GGAA microsatellite by a dCas9-KRAB system showed downregulation of NKX2-2 and SOX2 protein expression in A673 and SKNMC cells [33]. However, AHDC1 knockdown did not change the SOX2 protein level in A673 cells. We also tested whether AHDC1 knockdown reduces protein expression levels in other Ewing’s sarcoma cell lines. For this purpose, we treated Seki or NCR-EW2 cell lines, both of which have been established as Ewing’s sarcoma cells, with siAHDC1 RNA [20, 34]. EWS-FLI1 and NR0B1 were downregulated in both cell lines (S4C and S4D Fig). NKX2-2 was only downregulated in NCR-EW2 cells.

The NR0B1 gene harbors EWS-FLI1-bound GGAA microsatellites within its promoter region [35]. We cloned the NR0B1 promoter region upstream of Nanoluc and measured NR0B1 promoter activity in siAHDC1-treated cells (Fig 3B). AHDC1 knockdown showed downregulation of NR0B1 promoter activity in A673 cells. In addition, the ChIP assay using cells expressing doxycycline-induced 3xEGFP, 3xFLAG-EWS-FLI1, or 3xFLAG-AHDC1 showed that EWS-FLI1 and AHDC1 could bind the GGAA microsatellite on the NR0B1 promoter (S5A Fig). We also measured mRNA levels of target genes of EWS-FLI1 by RT-qPCR in siAHDC1-treated cells (Fig 3C). mRNA expression of PPP1R1A, GLI1, FoxM1, and NR0B1 genes highly expressed in Ewing’s sarcoma cells was dependent on EWS-FLI1 [35–38]. These genes were downregulated in siAHDC1-treated cells but not NKX2-2 and EWS-FLI1. We speculated that AHDC1 might affect EWS-FLI1 protein stability. EWS-FLI1 protein turnover is proteasome-dependent and may also be lysosomal-dependent [13, 39]. In AHDC1 knockdown cells, EWS-FLI1 protein amount reduced rapidly compared to the negative control after treatment of Cycloheximide, a protein synthesis inhibitor (S5B Fig). EWS-FLI1 and NKX2-2 protein amount increased by adding MG-132, a proteasome inhibitor, as previously reported (S5C Fig) [39, 40]. In AHDC1 knockdown cells, decreased EWS-FLI1 and NKX2-2 protein amount was not fully suppressed by MG-132 treatment. Although we do not examine the lysosomal degradation of EWS-FLI1, EWS-FLI1 might partially degrade in lysosome by independently proteasomal machinery, or AHDC1 might also regulate ubiquitination proteins. Alternatively, AHDC1 might affect the post-transcriptional machinery of EWS-FLI1 and NKX2-2. We need to elucidate this hypothesis in the future.

To determine whether the EWS-FLI1 protein is involved in AHDC1 gene expression, we performed an EWS-FLI1 knockdown (Fig 3D). AHDC1 protein expression level was not altered in shEWSR1 or shFLI1-treated cells. These results suggest that AHDC1 is involved in EWS-FLI1-mediated transcriptional regulation, at least by binding to the GGAA microsatellite region on the NR0B1 promoter. However, how AHDC1 regulates EWS-FLI1 protein levels still needs to be examined.

### AHDC1 knockdown attenuates cell growth in Ewing’s cells

**Fig 4:**
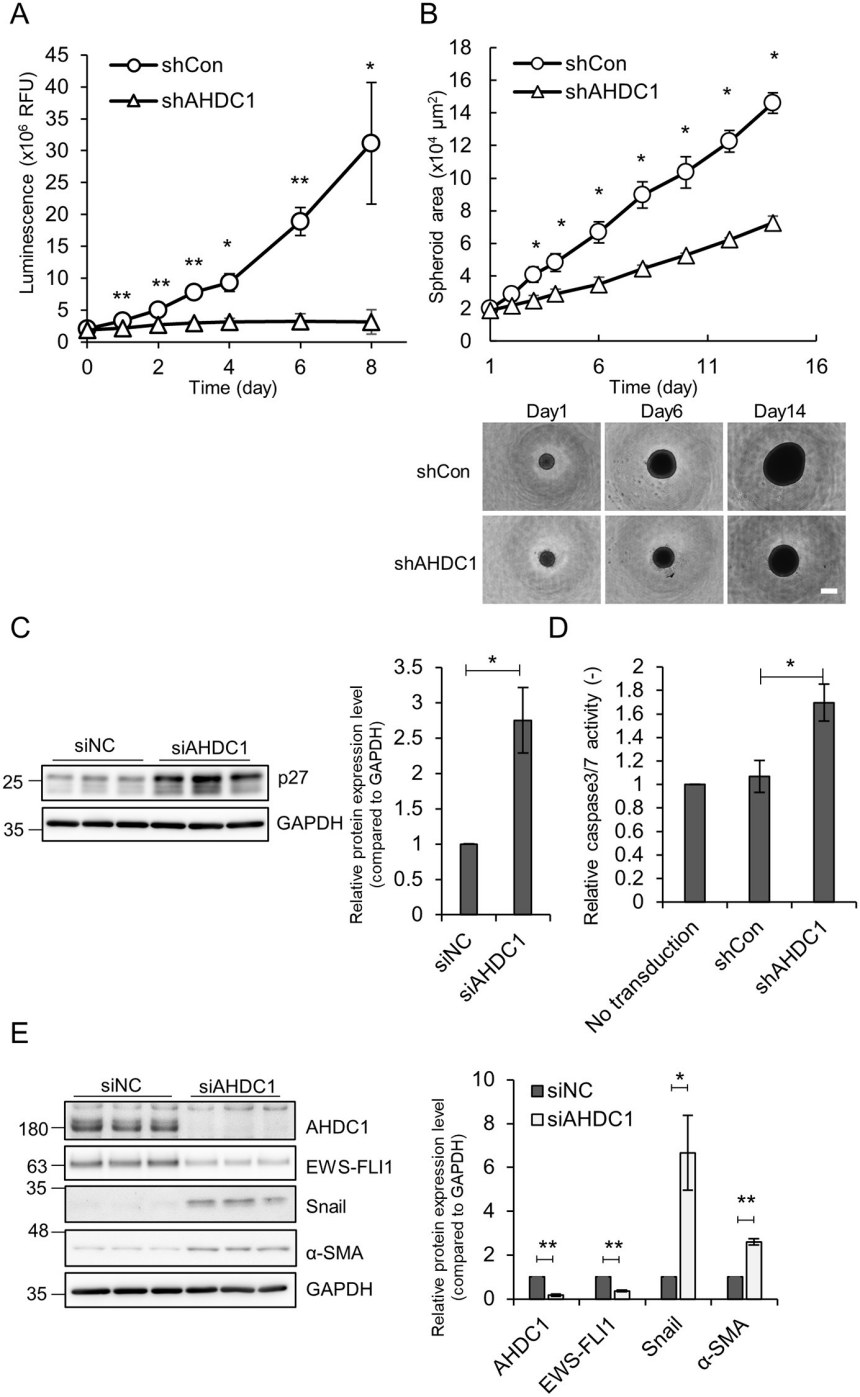
AHDC1 knockdown reduces cell growth in Ewing’s sarcoma cells. (A) Lentivirus expressing shRNA was transduced to A673 cells for 3 d. 1 × 10^3^ Cells were spread onto a 96-well plate and cultured again. Cell viability was determined by CellTiter-Glo2.0 on the indicated day. (B) shRNA-expressing cells were transferred into a 3D culture plate. The spheroid size was determined by a Keyence BZ-810X microscopy. Scale bar, 500 μm. (C) siAHDC1-treated A673 cells were cultured for 2 d. Western blotting analysis was performed by p27 and GAPDH antibodies. Relative values were normalized by GAPDH. (D) Lentivirus expressing shRNA was transduced to A673 cells for 3 d. Caspase activity was measured by a Caspase-Glo 3/7 Assay. (E) siAHDC1-treated cells were analyzed by western blotting. *P* values were calculated by the student’s t-test. * p<0.05; ** p<0.001.

EWS-ETS fusion proteins are essential for the cell growth of Ewing’s sarcoma. To test whether AHDC1 affects cell growth in Ewing’s sarcoma cells, we transduced shAHDC1-expressing lentivirus in A673 cells (S6 Fig). EWS-FLI1, NR0B1, and NKX2-2 protein expression were reduced in shAHDC1-expressing cells as well as in siAHDC1-treated cells. After lentivirus transduction, cells were collected and spread again onto the 96-well microplate, resulting in the reduction of cell growth (Fig 4A). In addition, the spheroid culture of shAHDC1-expressing cells also showed reduced cell growth in a 3D-culture well (Fig 4B). Seki and NCR-EW2 cells were also treated with shAHDC1-expressing lentivirus, resulting in the reduction of cell growth (S7A Fig). AHDC1 knockdown was performed in HEK293 or hTERT RPE-1 cells as non-Ewing’s cell types (S7B Fig). NR0B1 was weakly expressed in both cell lines but did not alter after shAHDC1 transduction (S7B Fig). HEK293 and hTERT RPE-1 cells did not show reduced cell growth after shAHDC1 transduction (S7C Fig). In addition, SAOS-2 and U-2 OS osteosarcoma cell lines did not alter any growth defects after shAHDC1 knockdown, suggesting that AHDC1 affects cell growth in Ewing’s sarcoma cells (S8 Fig).

Next, we assessed cell cycle progression and apoptotic activity after AHDC1 knockdown. siAHDC1-treated cells presented an increased p27 protein level (Fig 4C). In addition, shAHDC1-expressing cells showed a high caspase activity level (Fig 4D). EWS-FLI1 knockdown shows increased cell adhesion, migration, and invasion in Ewing sarcoma cells [41–43]. AHDC1 knockdown and EWS-FLI1 knockdown up-regulated Snail and α-smooth muscle actin (α-SMA), both of which are the epithelial-mesenchymal transition marker (Fig 4E and S9A Fig), but AHDC1 knockdown did not affect cell migration (S9B and S9C Fig). These results suggest that AHDC1 affects cell cycle progression and suppression of apoptosis in Ewing’s sarcoma cells.

### AHDC1 knockdown reduces BRD4 and BRG1 protein expression

**Fig 5:**
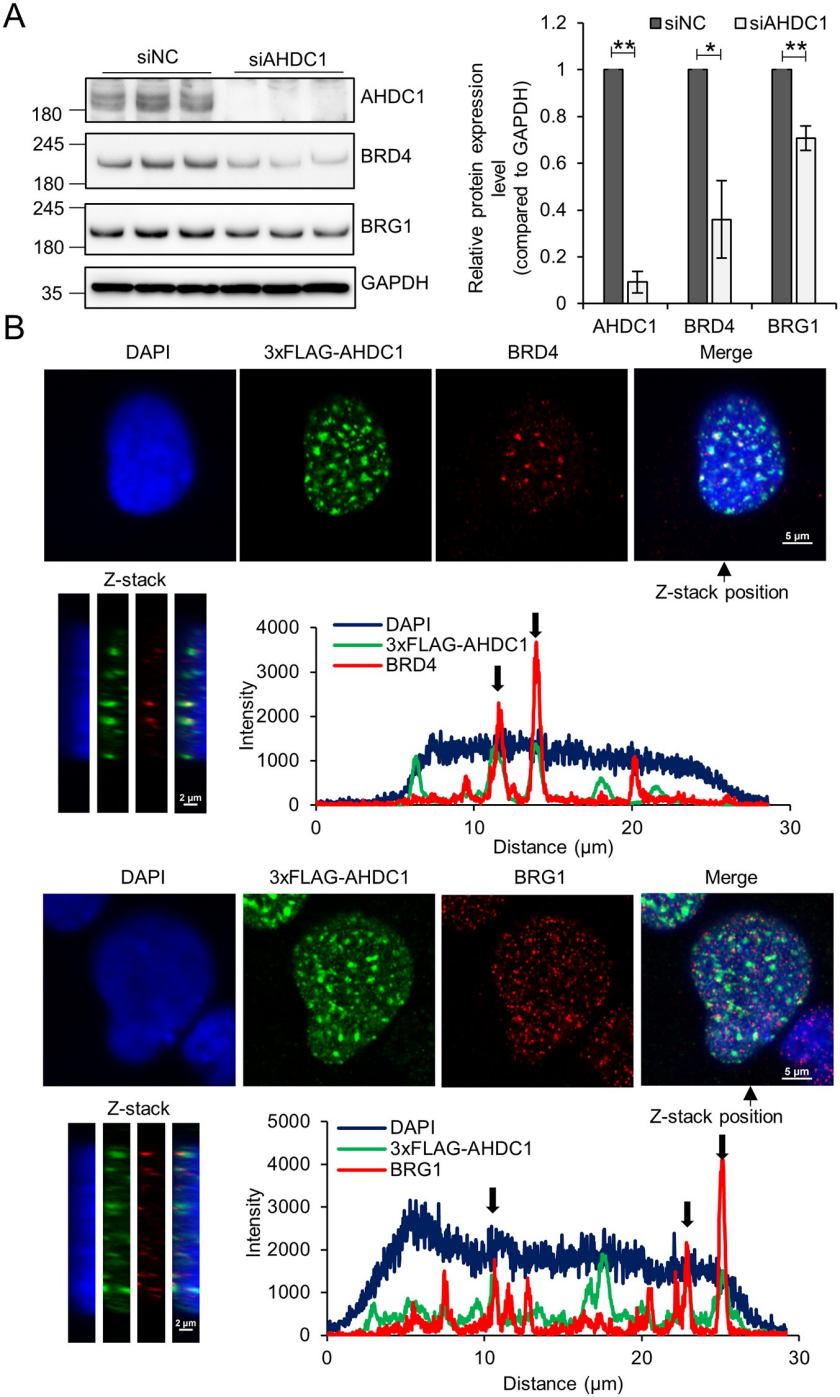
AHDC1 knockdown reduces BRD4 and BRG1 in A673 cells. (A) siAHDC1-treated cells were collected for western blotting. Each antibody detected the respective protein, and the relative value was normalized by GAPDH. (B) 3xFLAG-AHDC1 was induced by 0.1 μg/ml doxycycline for 1 day, fixed, and permeabilized. Scale bar, 5 μm. Z-stack scale bar, 2 μm. *P* values were calculated by the student’s t-test. * p<0.05; ** p<0.001.

In our proximal proteins screening of EWS-ETS fusion proteins, we also identified BRD4 and BRG1 (S2 Fig and S3 Table) [44]. BRD4 has been shown to interact with EWS-FLI1, and BRD4 inhibition by BET inhibitors results in reduced cell growth in Ewing’s sarcoma cells [9, 10, 45, 46]. EWS-FLI1 recruited BRG1/BRM-associated factor (BAF) complexes containing BRG1 to the GGAA microsatellite region [5]. We tested whether BRD4 and BRG1 protein expression levels are affected by AHDC1 knockdown (Fig 5A). AHDC1 knockdown showed reduced BRD4 and BRG1 protein expression levels. Fluorescent protein-tagged AHDC1 localized in the nuclei in Hela cells [47]. We expressed FLAG-tagged AHDC1 by using the *piggyBac* system under the control of the Tet-on system in A673 cells and stained with BRD4 and BRG1 (Fig 5B). AHDC1 was partially co-localized with endogenous BRG4 and BRG1 at the z-stack visualization. AHDC1 may contribute to stabilizing BRG1 and BRD4 proteins by co-localization. We need to clarify the relationship between AHDC1 and chromatin regulators in the future.

## Discussion

Proximal protein identification using new tools such as APEX2, BioID, or their derivatives has been a promising tool for biochemical approaches in vitro or in vivo [11]. In this study, we isolated AHDC1 as a proximal protein of the EWS-ETS proteins using the screening of the BioID system. AHDC1 was necessary to grow Ewing’s sarcoma cells but not non-Ewing’s sarcoma cells such as HEK293 or hTERT RPE-1 cells. In addition, AHDC1 affected gene expression of EWS-FLI1 target genes. Thus, AHDC1 may be one of the regulators for oncogenic function in Ewing’s sarcoma cells.

In BioID analysis using the three EWS-ETS fusion proteins, we identified 193 proteins (Fig 1C), while the 366 proteins were previously identified in 293 cells [10]. Twenty-one proteins overlapped in our list. We thought of three possible reasons for this difference: first, differences in purification methods. We purified the biotinylated proteins with streptavidin sepharose, digested, and collected them with Trypsin/Lys-C on beads. In the previous paper, the biotinylated proteins were purified with streptavidin agarose and separated by SDS-PAGE, followed by in-gel digestion with Trypsin [10]. In-gel digestion may have reduced the number of identified protein amounts. Second, there are cellular differences. We used an Ewing’s sarcoma cell line for BioID analysis, whereas the previous study used 293 cells [10]. These differences may be due to differences in gene expression patterns. Third, differences in negative controls. We used BioID and BioID-tagged Luc2 as negative controls. We searched protein amounts from precursor ions in each BioID sample and compared the amount of BirA(R118G) in each sample for normalization (Materials and methods). In previous data, negative controls were subtracted from the CRAPome database [44]. This difference may result in a difference in proteins identified by each BioID method. Our raw data of the BioID screening contained much contamination that non-specifically binds to streptavidin Sepharose beads because 3879 proteins were identified, but 193 proteins were only found as proximity proteins of EWS-ETS fusion proteins. Hence, the purification method needs to be improved, including the type of beads and washing method.

We also checked BioID-tagged fusion protein expression. Doxycycline-induced BioID-tagged EWS-ETS proteins were highly expressed compared to endogenous EWS-FLI1 (S1A Fig). Although several proteins could be reproduced in the band after purification of streptavidin Sepharose without ARNT, RBM26, and IRX4 (S3 Fig), we do not subtract the truncated EWS or ETS fusion proteins as the negative control. We guess that our list might include proximal proteins of intact EWSR1, FLI1, ERG, and ETV4. In addition, excessive induction may lead to nonspecific biotinylation, and nonspecific biotinylated proteins may be included in the list of proteins we have identified.

In 193 proteins, ARID1A, BRG1 (SMARCA4), BRD4, SMN1, SMARCC1, SF1, SMARCB1, and SNRPC (U1-C) have been known as interacting proteins for EWS-FLI1 or EWSR1 [10, 28, 48–51]. We added the BioID-tag to the N-terminal side of the EWS-ETS fusion protein and the BirA biotinylated proximal proteins in a location-dependent manner. Therefore, our experiments may not have identified the protein that interacts with the C-terminus of the EWS-ETS fusion protein.

The Xia-Gibbs syndrome has been identified as a de novo heterozygous truncating mutation of AHDC1 [14]. More than 100 cases of mutations related to the diagnosis of the Xia-Gibbs syndrome have been reported [15]. Not only heterozygous mutations of AHDC1 but also micro-duplication of the genome containing the AHDC1-coding region showed similar symptoms [52]. Thus, deregulation of AHDC1 gene expression affects the developmental process. AHDC1 has an AT-hook DNA binding motif, a PDZ motif, and other conserved domains within the coding sequence [47]. Feng *et al*. showed that AHDC1 expression was highly expressed in cervical cancer cells compared with immortalized cervical epithelium, and its expression was regulated by a long noncoding RNA, LINC01133 [53]. However, the molecular mechanisms for AHDC1 in cancer cells are still unclear.

EWSR1 is an RNA-binding protein comprising FET family proteins (FUS, TAF15, and EWSR1). EWSR1 is also one of the paraspeckle components that is a subcellular body in the nucleus and co-localized with SFPQ1, NONO, and PSPC1 [54, 55]. AHDC1 was also isolated as one of the paraspeckle components co-localized with EWSR1 [54]. Khayat *et al*. showed that wild-type AHDC1 localized in the nucleus, and Xia-Gibbs patients with a mutation of AHDC1 have disrupted wild-type AHDC1 localization in HeLa cells [47]. Our proximal proteins screening of EWS-ETS fusion proteins did not isolate SFPQ, NONO, or PSPC1. However, CPSF5 (NUDT21), CPSF6, and CPSF7 were isolated as paraspeckle components and are the components of the cleavage factor Im (CFIm) complex that brings about cleavage of 3’UTR of mRNA for polyadenylation were isolated as proximal proteins of EWS-ETS fusion proteins (S2 Fig and S3 Table) [54]. These results suggest that some paraspeckle components may interact with transcriptional complexes with EWS-ETS fusion proteins.

FET family proteins comprising FUS, EWSR1, and TAF15 are not only involved in neurodegenerative disease but also act as oncoproteins in sarcoma or leukemia by chromosomal translocation. The N-terminal region of FET family proteins comprising SYGQ-rich regions interacts with the SWI/SNF chromatin remodeling complex containing BRG1 [5, 28]. In our screening, the chromatin remodeling complex containing BRG1, ARID1A, SMARCC1, SMARCD1, SMARCE1, SMARCB1, and SMARCAL1 were isolated as proximal proteins of EWS-ETS fusion proteins (S2 Fig and S3 Table). EWS-FLI1 recruits BRG1 to open the chromatin structure at the GGAA microsatellite region [5]. In our observations, AHDC1 contributed to maintaining the BRG1 protein expression level (Fig 5A and 5B). We postulate that the SWI/SNF chromatin remodeling complex may regulate EWS-FLI1 transcriptional activity with AHDC1.

AHDC1 did not regulate the gene expression of EWS-FLI1 at the transcriptional level, while EWS-FLI1 protein was downregulated (Fig 3A and 3C). Our results also showed that a proteasomal inhibitor did not fully suppress the EWS-FLI1 protein level in AHDC1 knockdown (S5C Fig). AHDC1 might affect the lysosomal pathway for EWS-FLI1 protein level or might affect EWS-FLI1 protein level at post-transcriptional or protein synthesis level. We still need to continue this hypothesis. AHDC1 might stabilize BRD4 and BRG1 (Fig 5A). In addition, FLAG-tagged AHDC1 expression partially co-localized with BRD4 and BRG1 in Ewing’s sarcoma cells (Fig 5B). We hypothesize that AHDC1 might be one of the accessory proteins needed to stabilize BRD4, BRG1, and probably EWS-FLI1 in Ewing’s sarcoma cells.

Recently, Gibbin, a protein that encodes the AHDC1 gene, mediates connections between enhancers and promoters at the specific gene locus during development [50]. Proximity labeling of Gibbin showed that Gibbin mainly interacts with zinc-finger transcription factors and DNA methylation regulators. Gibbin loss caused hypermethylation and decreased CTCF deposition in BMP4/Retinoic acid-treated Human embryonic stem cells. ARID1A, FUBP1, and ZNF462 in Gibbin interactome were included in our EWS-ETS fusion proteins list. Although the protein size of the Gibbin and AHDC1 protein that we analyzed are different, we guess that AHDC1 might be one of the hubs between enhancers and promoters in the Ewing sarcoma cells and partially affect gene expression of the EWS-ETS fusion proteins.

Finally, we only performed experiments on AHDC1 using Ewing’s sarcoma cell line, and we need to progress a cell line-derived xenograft model or patient-derived xenograft model using mice. In addition, we need to analyze the difference between AHDC1 low state and EWS-FLI1 low state because AHDC1 knockdown did not fully complete transcriptional activity by EWS-FLI1 knockdown in the future.

## Supporting information

S1 Fig BioID-tagged EWS-ETS fusion protein expression inhibits cell growth. (A) Cells expressing each BioID-tagged protein were induced with 1 μg/ml doxycycline for 1 d and used for western blotting. Each protein was detected by its respective antibody. (B) 1 × 10^3^ Cells were spread onto a 96-well plate and cultured for 1 d. Cells were treated 1 μg/ml of doxycycline, and cell viability was determined by CellTiter-Glo2.0 on the indicated day.
(TIF)

S2 Fig Network analysis of proximal proteins of EWS-ETS fusion proteins by STRING. Data was visualized by STRING database. Six clusters were categorized by k-means clustering. The network edges indicate both functional and physical protein associations.
(TIF)

S3 Fig Western blotting analysis of biotinylated proteins using streptavidin sepharose. Cells expressing each BioID-tagged protein by 1 μg/ml doxycycline for 1 d were lysed and purified by Streptavidin sepharose. Each protein was detected by its respective antibody.
(TIF)

S4 Fig AHDC1 knockdown of Seki and NCR-EW2 cells. (A) Scheme of western blotting after purification of biotinylated proteins using Streptavidin sepharose. (B) The nuclear lysate was mixed with DDDDK magnetic beads and detected by its respective antibody. (C) siAHDC1-treated Seki cells were cultured for 2 d. Each protein was detected by its respective antibody. (D) siAHDC1-treated NCR-EW2 cells were cultured for 2 d. Each protein was detected by its respective antibody. *P* values were calculated by the student’s t-test. * p<0.05; ** p<0.001.
(TIF)

S5 Fig ChIP assay and protease inhibitor treatment in siAHDC1 knockdown. (A) For a ChIP assay, cells were expressed 3xFLAG-tagged EGFP, EWS-FLI1, and AHDC1 by 1 μg/ml doxycycline for 1 d. DDDDK magnetic beads purified cross-linked chromatin. The NR0B1 promoter that harbors a GGAA microsatellite region was performed using KOD one polymerase with NR0B1-ChIP-f and NRoB1-ChIP-r primers at 35 cycles. The ChIP assay was performed in three independent replicas. (B) Cells after treatment of siAHDC1 RNA were performed for 2 d, treated with 20 μg/ml Cycloheximide for 8 h, and lysed by 1xSDS sample buffer. Each protein was detected by its respective antibody. (C) Cells after treatment of siAHDC1 RNA were performed for 2 d, treated with 10 μM MG-132 for 8 h, and lysed by 1xSDS sample buffer. Each protein was detected by its respective antibody.
(TIF)

S6 Fig AHDC1 knockdown of A673 cells by lentivirus expressing shRNA. Lentivirus expressing shRNA was transduced to A673 cells for 3 d. Each protein was detected by its respective antibody. *P* values were calculated by the student’s t-test. * p<0.05; ** p<0.001.
(TIF)

S7 Fig AHDC1 knockdown shows reduced growth of Seki and NCR-EW2 by lentivirus expressing shRNA. (A) Lentivirus expressing shRNA was transduced to Seki or NCR-EW2 cells for 3 d. 1 × 10^3^ Cells were spread onto a 96-well plate and cultured again. Cell viability was determined by CellTiter-Glo2.0 on the indicated day. (B) Lentivirus expressing shRNA was transduced to HEK293 or hTERT RPE-1 cells. Each protein was detected by its relative antibody. (C) Lentivirus expressing shRNA was transduced to HEK293 or hTERT RPE-1 cells for 3 d. 1 × 10^3^ Cells were spread onto a 96-well plate and cultured again. Cell viability was determined by CellTiter-Glo2.0 on the indicated day. *P* values were calculated by the student’s t-test. * p<0.05; ** p<0.001.
(TIF)

S8 Fig Osteosarcoma cell lines do not show growth defects after shAHDC1 RNA-expressing lentivirus transduction. Cells were transduced by shAHDC1 lentivirus for 3 d. Cell viability was determined by CellTiter-Glo2.0 on the indicated day. (A) SAOS-2 cells. (B) U-2 OS cells. *P* values were calculated by the student’s t-test. * p<0.05.
(TIF)

S9 Fig Snail and α-SMA expression after EWS-FLI1 knockdown and scratch wound healing assay after AHDC1 knockdown. (A) A673 cells were transduced by shEWS or shFLI1 lentivirus for 3 d and lysed by RIPA buffer for western blotting. Each protein was detected by its relative antibody. (B) A673 cells were transduced by shAHDC1 lentivirus for 3 d and scratched on a dish. Cells were cultured in DMEM without FBS on the indicted day. Scale bar, 500 μm. *P* values were calculated by the student’s t-test. * p<0.05; ** p<0.001.
(TIF)

S1 Table Primers for shRNA. (XLSX)

S2 Table Primers for RT-qPCR. (XLSX)

S3 Table LC-MS data of each BioID sample. (XLSX)

S4 Table Clustering data of STRING database. (XLSX)

S1 Raw images (PDF)
