# Supplementary figures and images for: AT-hook DNA-binding motif-containing protein one knockdown downregulates EWS-FLI1 transcriptional activity in Ewing’s sarcoma cells
Source: PLoS One. 2022 Oct 4;17(10):e0269077. doi: 10.1371/journal.pone.0269077 (PMC9531837; doi:10.1371/journal.pone.0269077)

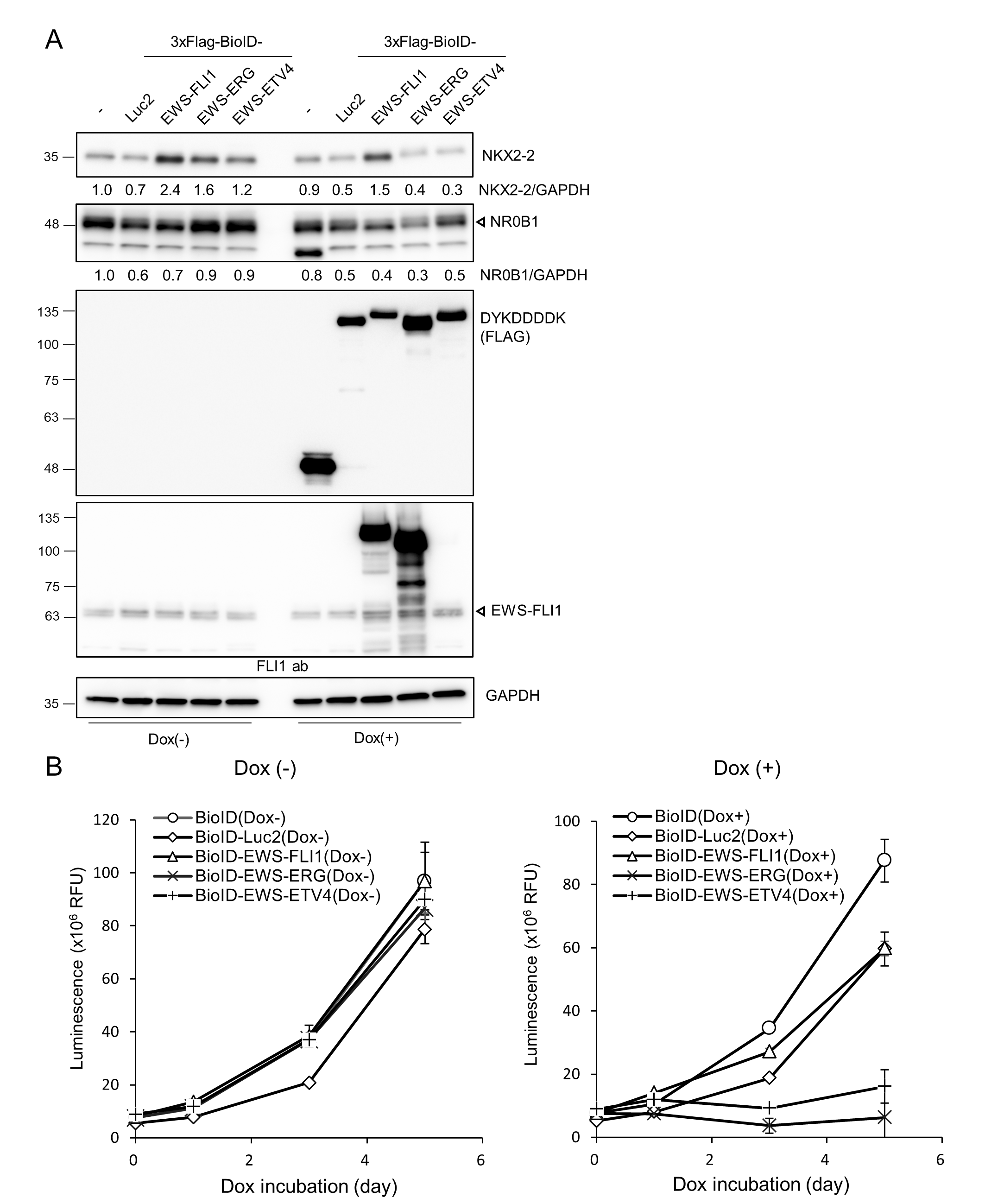

Supplement: S1 Fig — (A) Cells expressing each BioID-tagged protein were induced with 1 μg/ml doxycycline for 1 d and used for western blotting. Each protein was detected by its respective antibody. (B) 1 × 10^3^ Cells were spread onto a 96-well plate and cultured for 1 d. Cells were treated 1 μg/ml of doxycycline, and cell viability was determined by CellTiter-Glo2.0 on the indicated day. (TIF) [file pone.0269077.s001.tif]

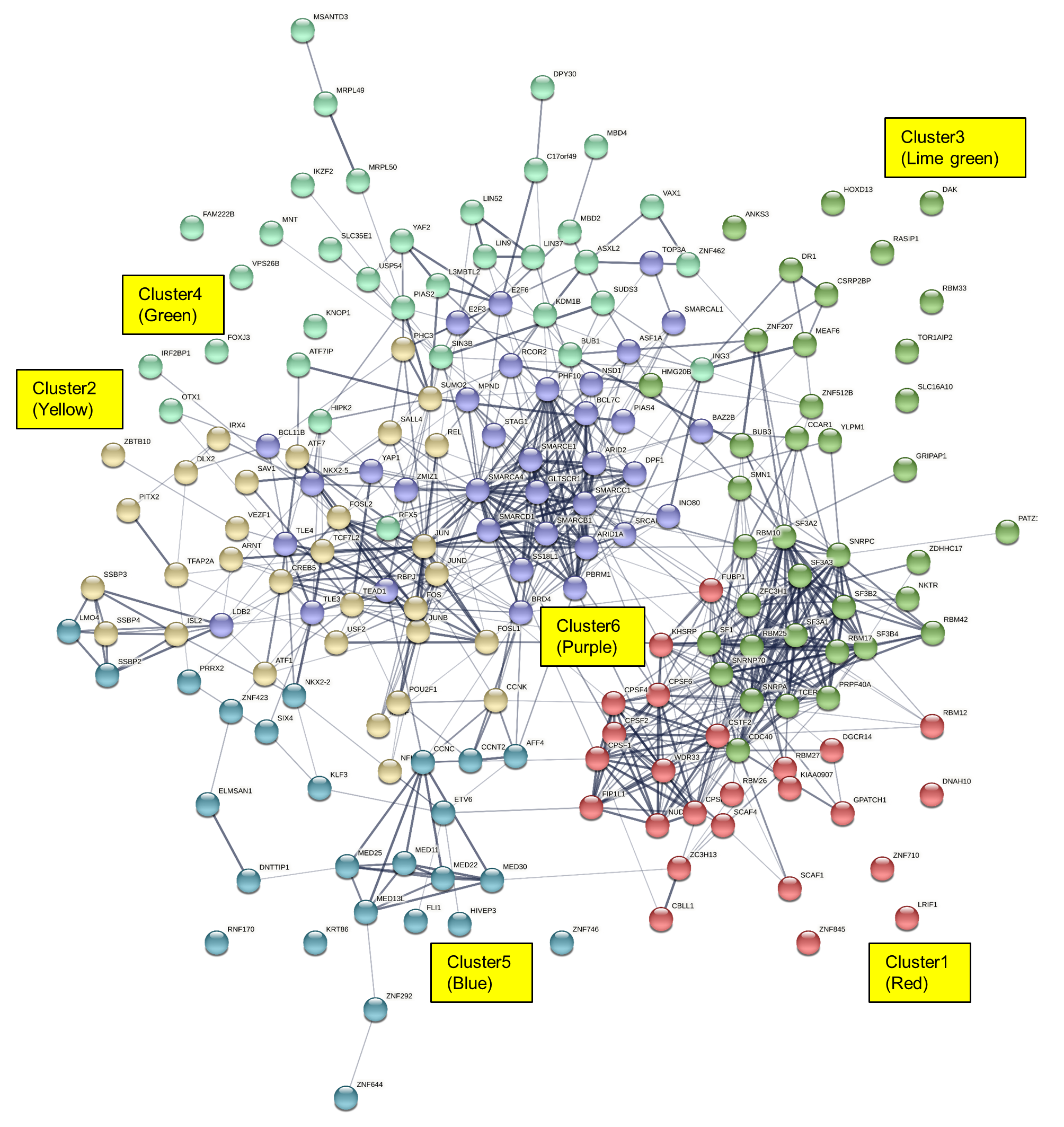

Supplement: S2 Fig — Data was visualized by STRING database. Six clusters were categorized by k-means clustering. The network edges indicate both functional and physical protein associations. (TIF) [file pone.0269077.s002.tif]

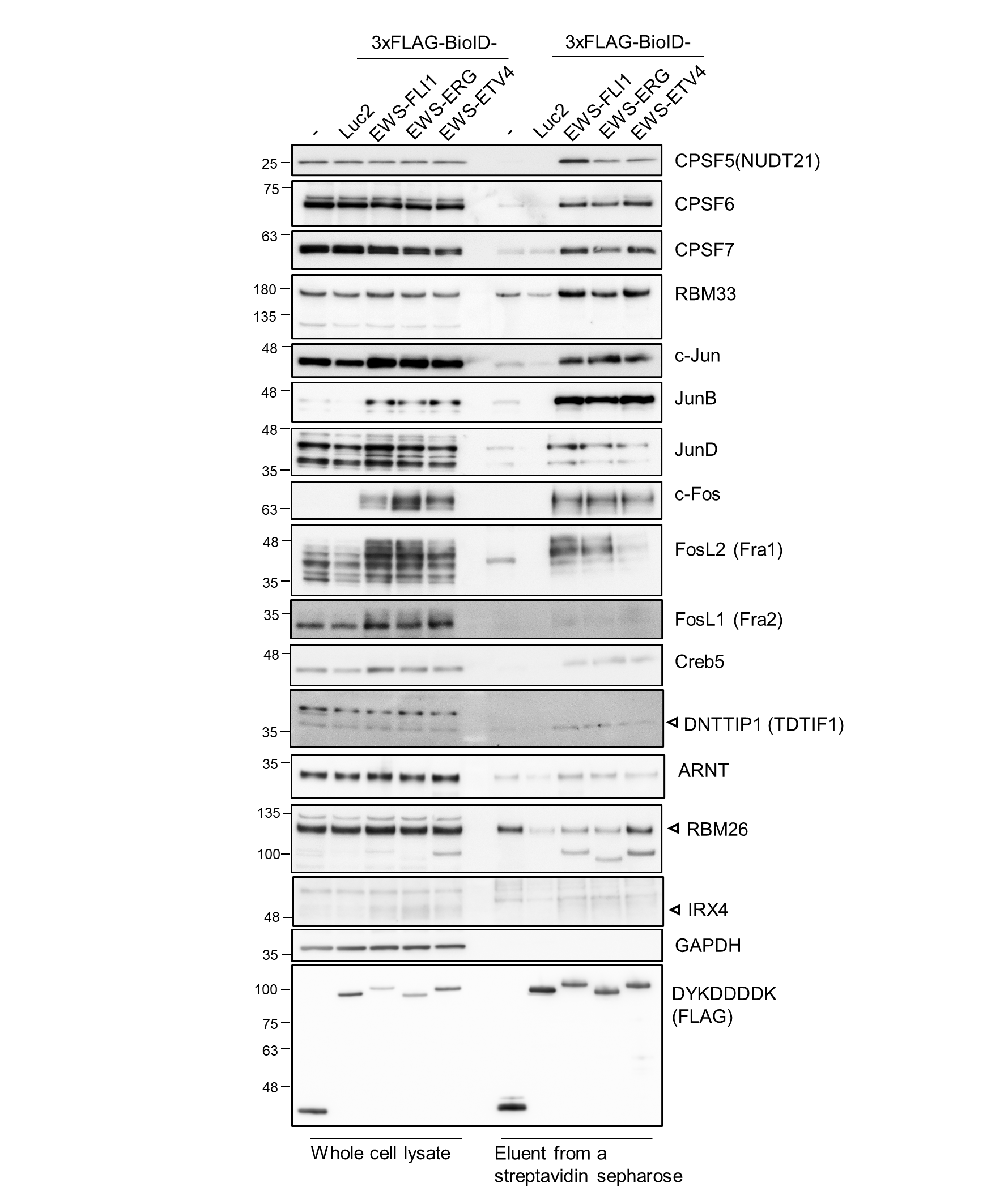

Supplement: S3 Fig — Cells expressing each BioID-tagged protein by 1 μg/ml doxycycline for 1 d were lysed and purified by Streptavidin sepharose. Each protein was detected by its respective antibody. (TIF) [file pone.0269077.s003.tif]

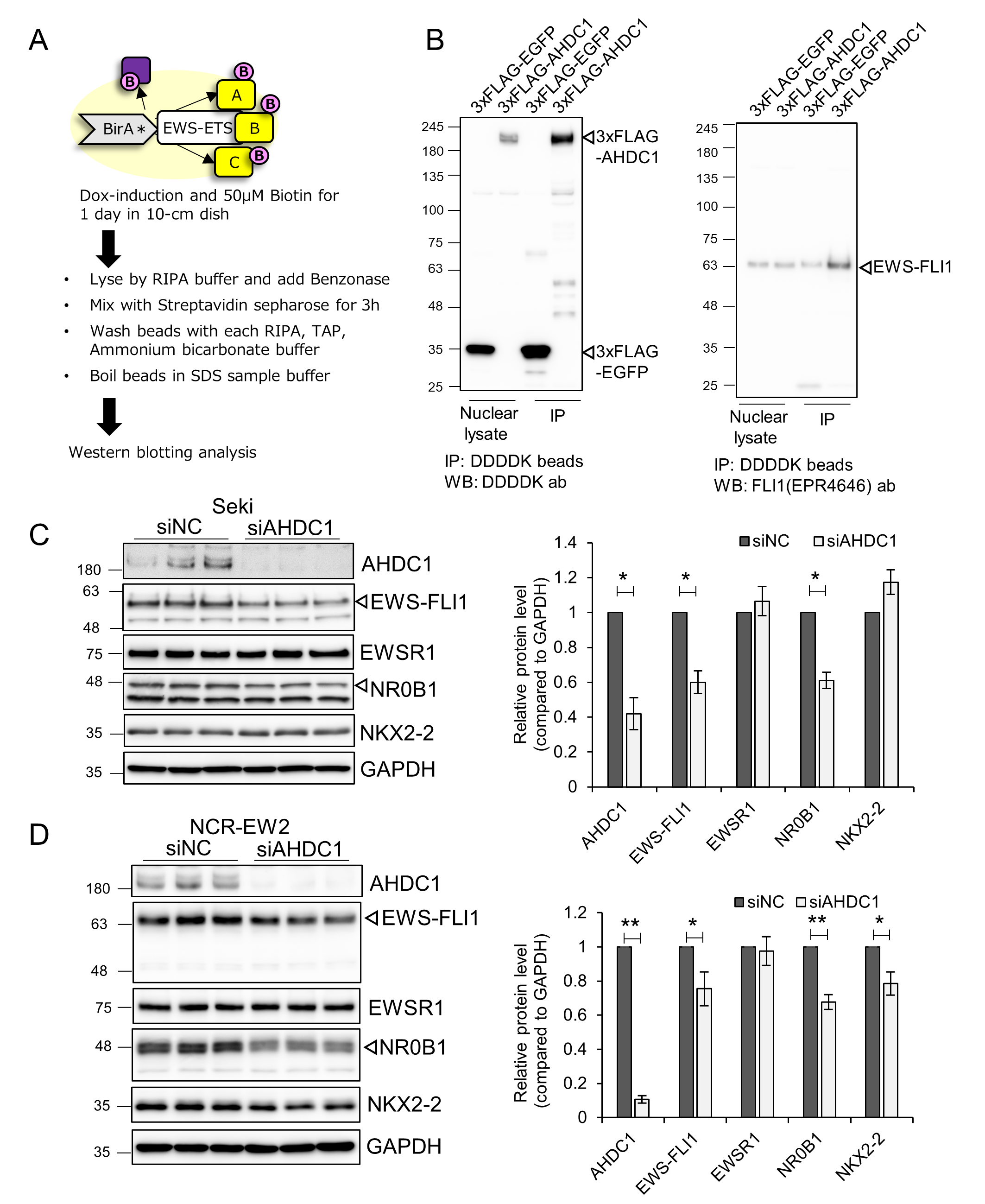

Supplement: S4 Fig — (A) Scheme of western blotting after purification of biotinylated proteins using Streptavidin sepharose. (B) The nuclear lysate was mixed with DDDDK magnetic beads and detected by its respective antibody. (C) siAHDC1-treated Seki cells were cultured for 2 d. Each protein was detected by its respective antibody. (D) siAHDC1-treated NCR-EW2 cells were cultured for 2 d. Each protein was detected by its respective antibody. P values were calculated by the student’s t-test. * p<0.05; ** p<0.001. (TIF) [file pone.0269077.s004.tif]

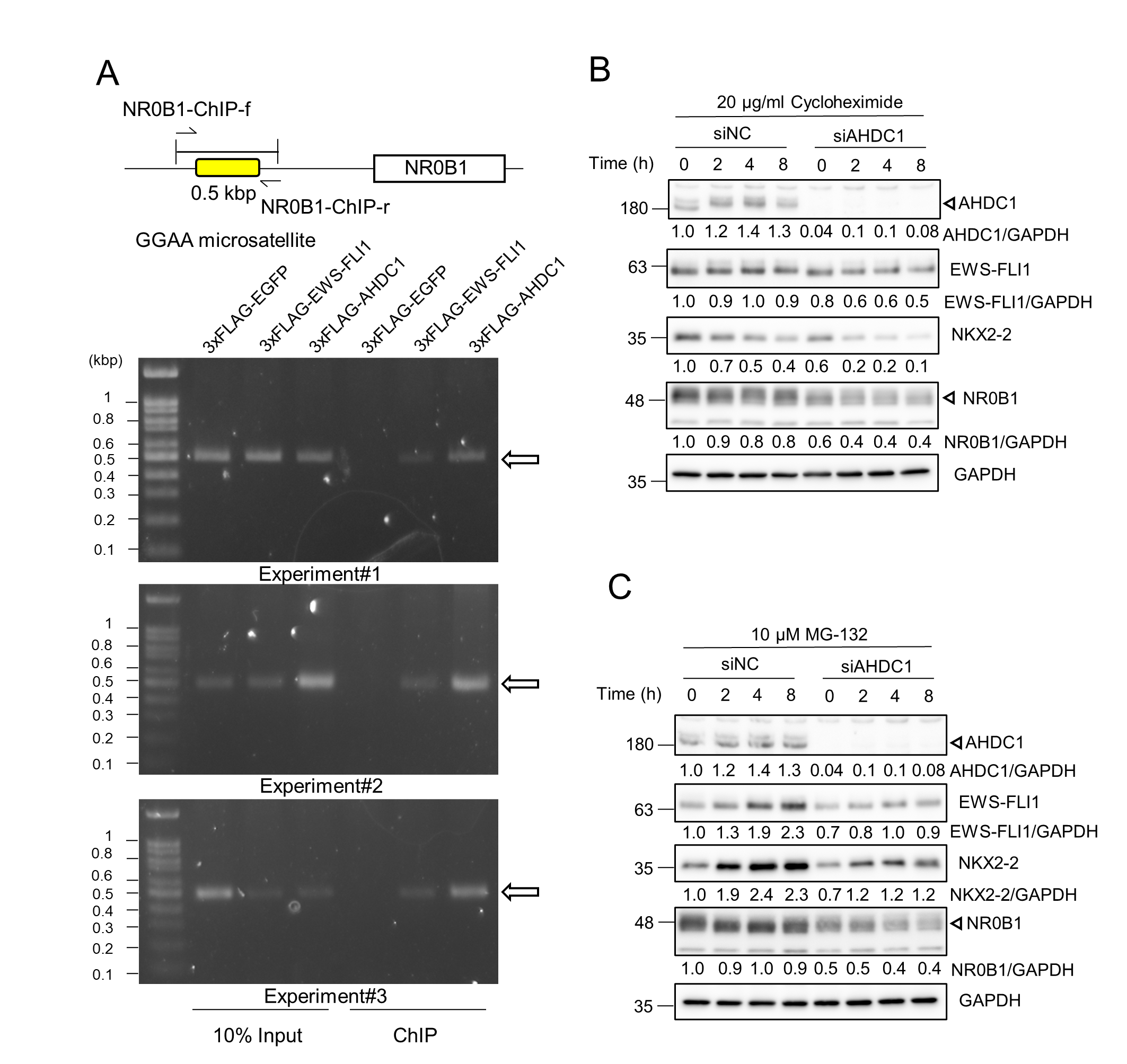

Supplement: S5 Fig — (A) For a ChIP assay, cells were expressed 3xFLAG-tagged EGFP, EWS-FLI1, and AHDC1 by 1 μg/ml doxycycline for 1 d. DDDDK magnetic beads purified cross-linked chromatin. The NR0B1 promoter that harbors a GGAA microsatellite region was performed using KOD one polymerase with NR0B1-ChIP-f and NRoB1-ChIP-r primers at 35 cycles. The ChIP assay was performed in three independent replicas. (B) Cells after treatment of siAHDC1 RNA were performed for 2 d, treated with 20 μg/ml Cycloheximide for 8 h, and lysed by 1xSDS sample buffer. Each protein was detected by its respective antibody. (C) Cells after treatment of siAHDC1 RNA were performed for 2 d, treated with 10 μM MG-132 for 8 h, and lysed by 1xSDS sample buffer. Each protein was detected by its respective antibody. (TIF) [file pone.0269077.s005.tif]

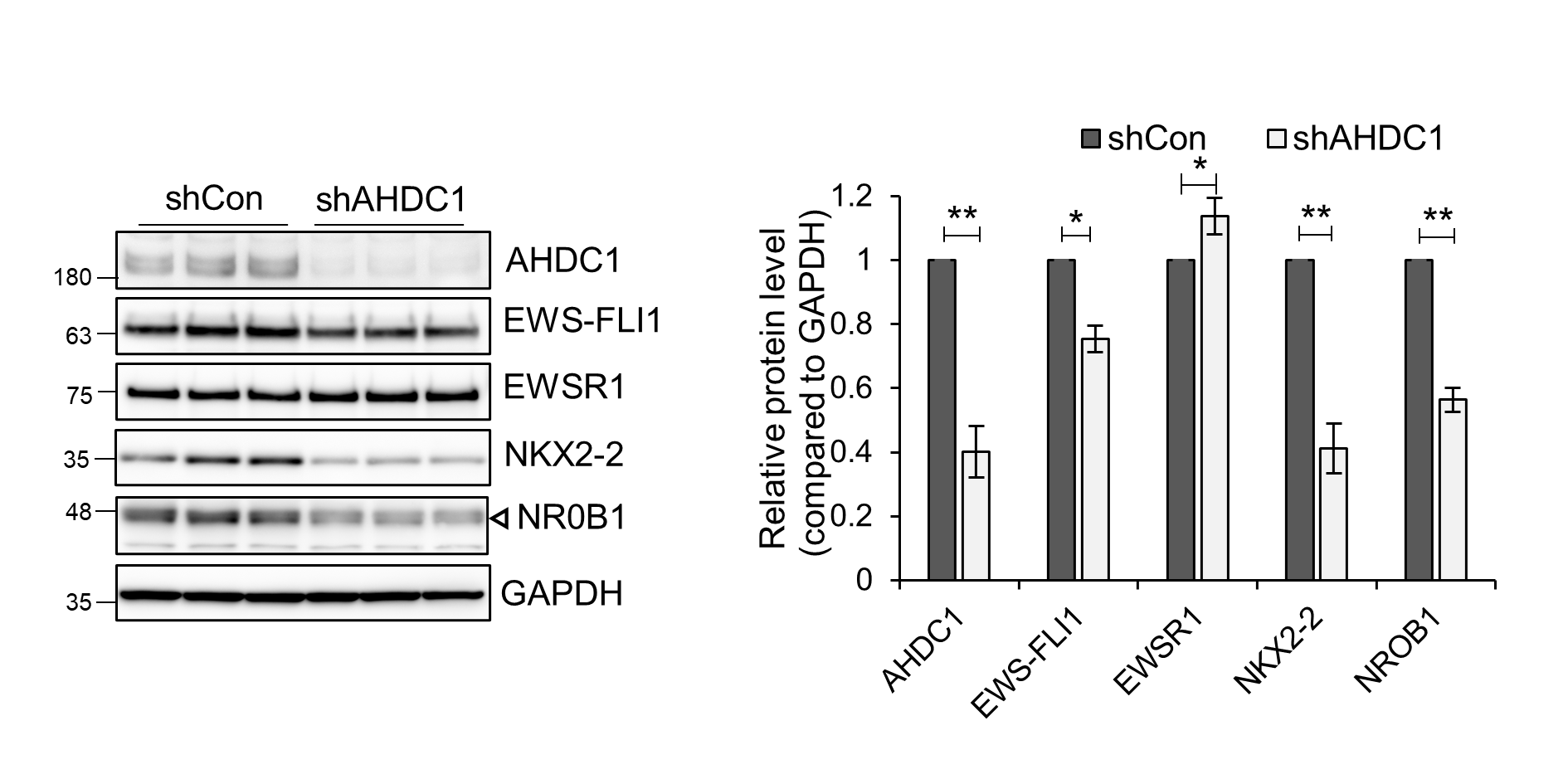

Supplement: S6 Fig — Lentivirus expressing shRNA was transduced to A673 cells for 3 d. Each protein was detected by its respective antibody. P values were calculated by the student’s t-test. * p<0.05; ** p<0.001. (TIF) [file pone.0269077.s006.tif]

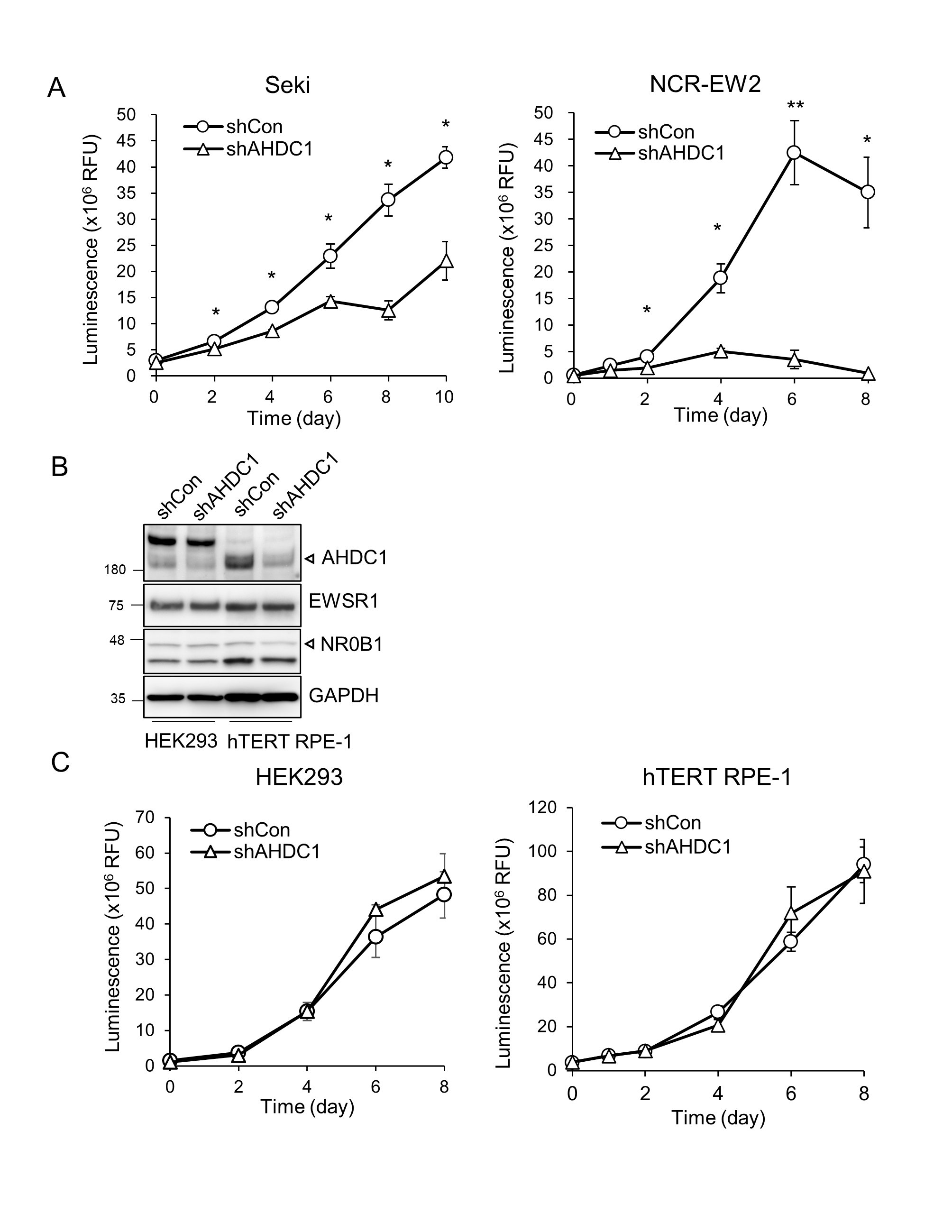

Supplement: S7 Fig — (A) Lentivirus expressing shRNA was transduced to Seki or NCR-EW2 cells for 3 d. 1 × 10^3^ Cells were spread onto a 96-well plate and cultured again. Cell viability was determined by CellTiter-Glo2.0 on the indicated day. (B) Lentivirus expressing shRNA was transduced to HEK293 or hTERT RPE-1 cells. Each protein was detected by its relative antibody. (C) Lentivirus expressing shRNA was transduced to HEK293 or hTERT RPE-1 cells for 3 d. 1 × 10^3^ Cells were spread onto a 96-well plate and cultured again. Cell viability was determined by CellTiter-Glo2.0 on the indicated day. P values were calculated by the student’s t-test. * p<0.05; ** p<0.001. (TIF) [file pone.0269077.s007.tif]

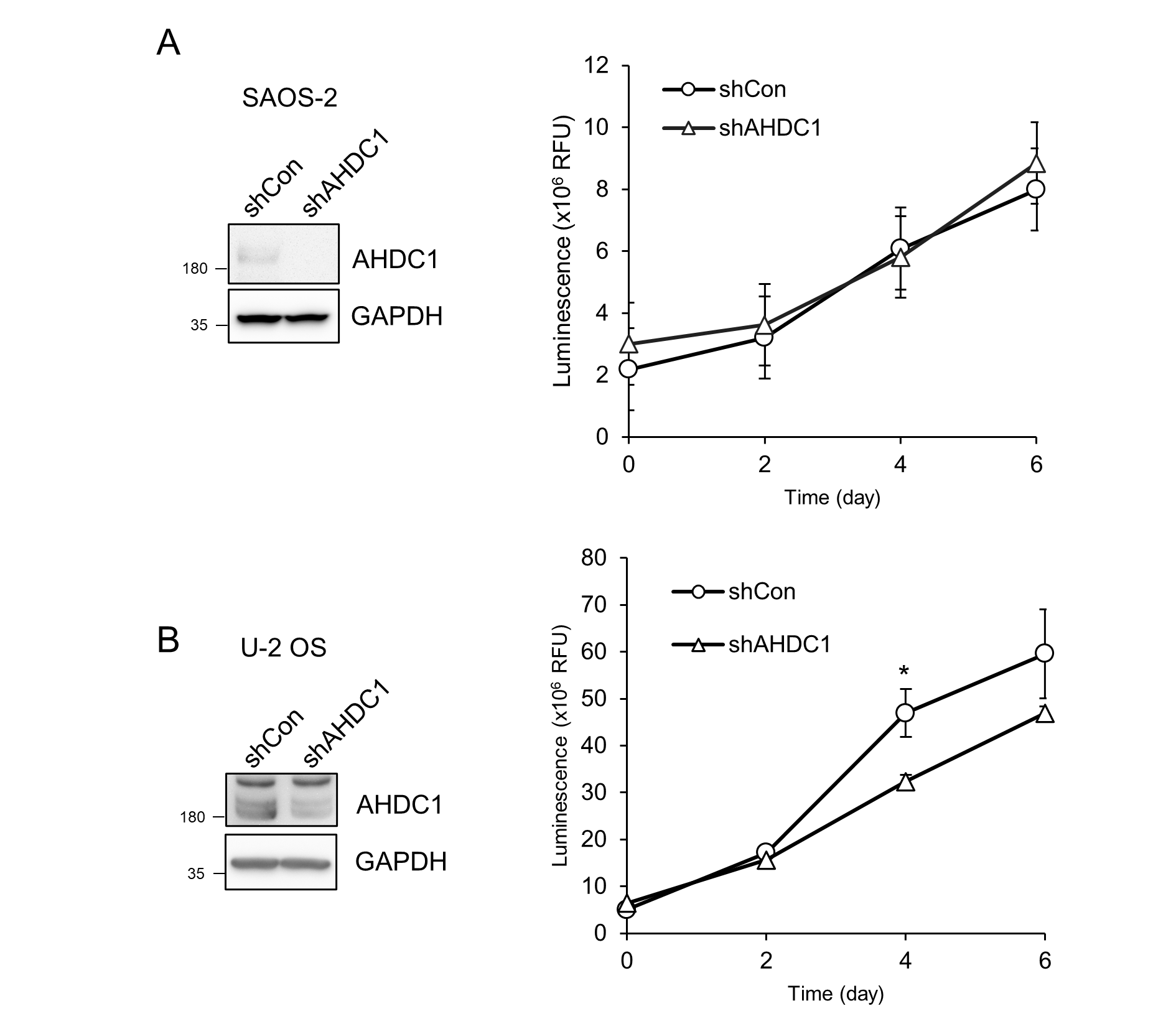

Supplement: S8 Fig — Cells were transduced by shAHDC1 lentivirus for 3 d. Cell viability was determined by CellTiter-Glo2.0 on the indicated day. (A) SAOS-2 cells. (B) U-2 OS cells. P values were calculated by the student’s t-test. * p<0.05. (TIF) [file pone.0269077.s008.tif]

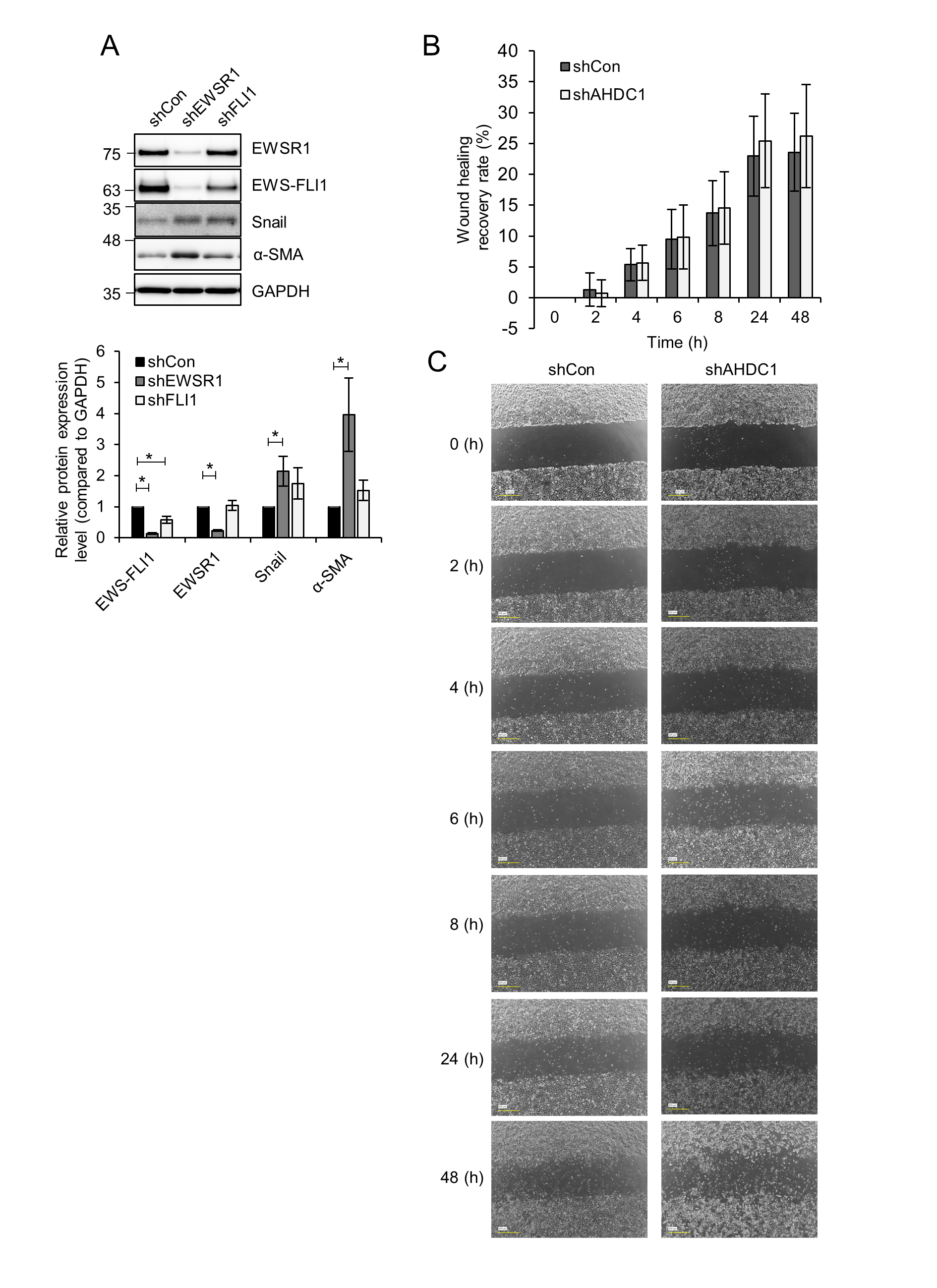

Supplement: S9 Fig — (A) A673 cells were transduced by shEWS or shFLI1 lentivirus for 3 d and lysed by RIPA buffer for western blotting. Each protein was detected by its relative antibody. (B) A673 cells were transduced by shAHDC1 lentivirus for 3 d and scratched on a dish. Cells were cultured in DMEM without FBS on the indicted day. Scale bar, 500 μm. P values were calculated by the student’s t-test. * p<0.05; ** p<0.001. (TIF) [file pone.0269077.s009.tif]
