# Supplementary material for: AT-hook DNA-binding motif-containing protein one knockdown downregulates EWS-FLI1 transcriptional activity in Ewing’s sarcoma cells
Source: PLoS One. 2022 Oct 4;17(10):e0269077. doi: 10.1371/journal.pone.0269077 (PMC9531837; doi:10.1371/journal.pone.0269077)
Supplement: S1 Raw images — (PDF) [file pone.0269077.s014.pdf]

Supporting Information

Original Western blots and gel images

AT-hook DNA-binding motif-containing protein one knockdown downregulates EWS-FLI1 transcriptional activity in Ewing's sarcoma cells

Takao Kitagawa[1*], Daiki Kobayashi[2,3], Byron Baron[4], Hajime Okita[5], Tatsuo Miyamoto[6], Rie Takai[1], Durga Paudel[1], Tohru Ohta[1], Yoichi Asaoka[7], Masayuki Tokunaga[8], Koji Nakagawa[1], Makoto Furutani-Seiki[7], Norie Araki[3], Yasuhiro Kuramitsu[1], Masanobu Kobayashi[1]

*Corresponding author
takao-k@hoku-iryo-u.ac.jp

1 Advanced Research Promotion Center, Health Sciences University of Hokkaido, 1757, Kanazawa, Ishikari-Tobetsu, Hokkaido, 061-0293, Japan

2 Department of Omics and Systems Biology, Graduate School of Medical and Dental Sciences, Niigata University, 757 Ichibancho, Asahimachi-dori, Chuo-ku, Niigata, 951-8510, Japan

3 Department of Tumor Genetics and Biology, Faculty of Life Sciences, Kumamoto University, Kumamoto-Shi, Kumamoto, 860-8556, Japan

4 Center for Molecular Medicine and Biobanking, University of Malta, Msida, MSD2080, Malta

5 Division of Diagnostic Pathology, Keio University School of Medicine, Shinano, Shinjuku-ku, Tokyo, 160-8582, Japan

6 Department of Molecular and Cellular Physiology, Yamaguchi University Graduate School of Medicine, Ube, Yamaguchi, 755-8505, Japan

7 Department of Systems Biochemistry in Pathology and Regeneration, Yamaguchi University Graduate School of Medicine, Ube, Yamaguchi, 755-8505, Japan

8 Department of Obstetrics and Gynecology, Yamaguchi University Graduate School of Medicine, Ube, Yamaguchi, 755-8505, Japan

Fig 1B

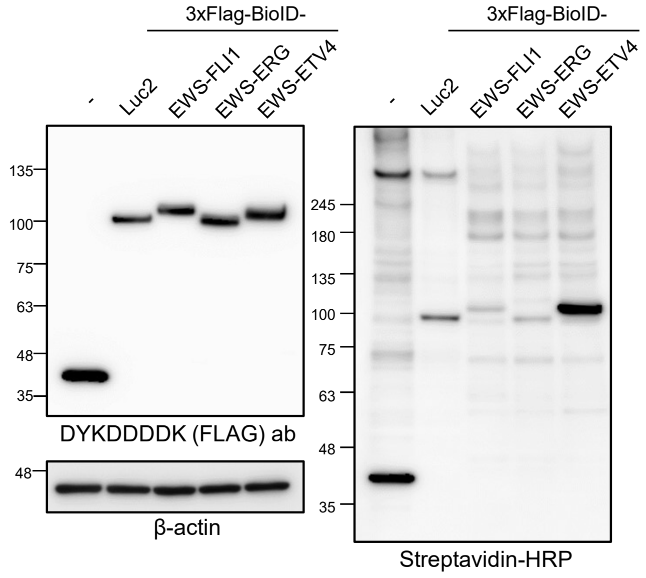

Raw data

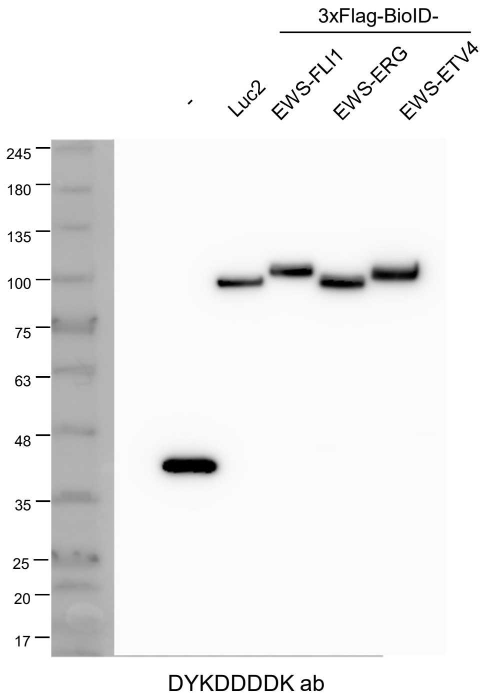

Raw data

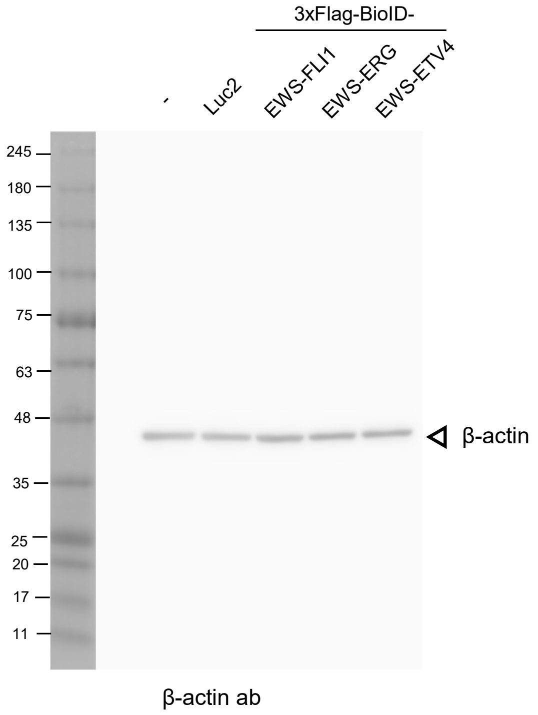

Raw data

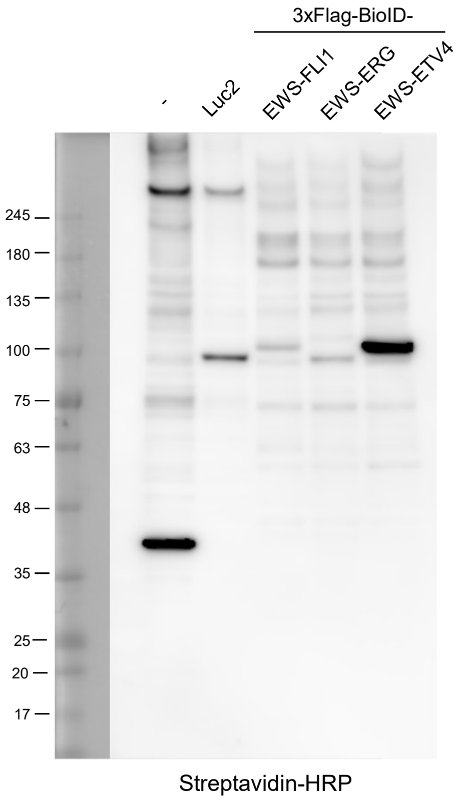

Fig 2A

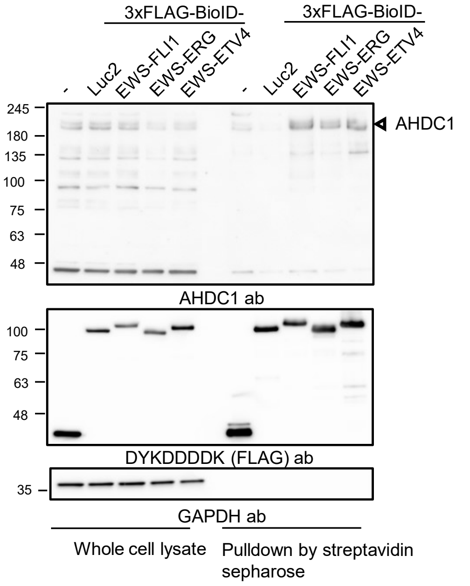

Raw data

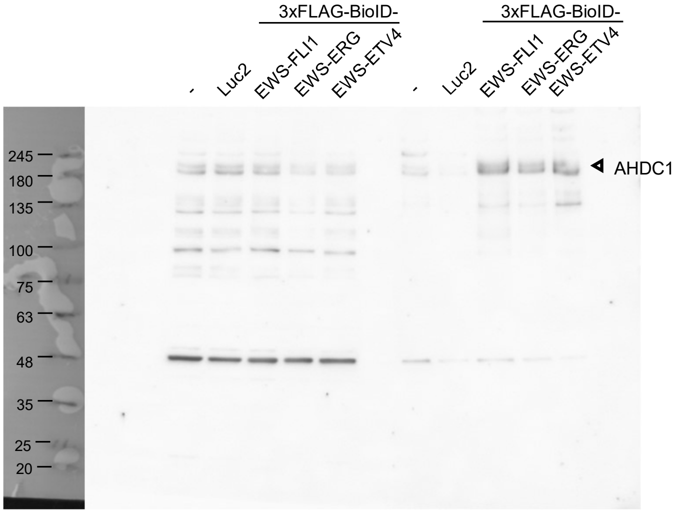

AHDC1 ab

Raw data

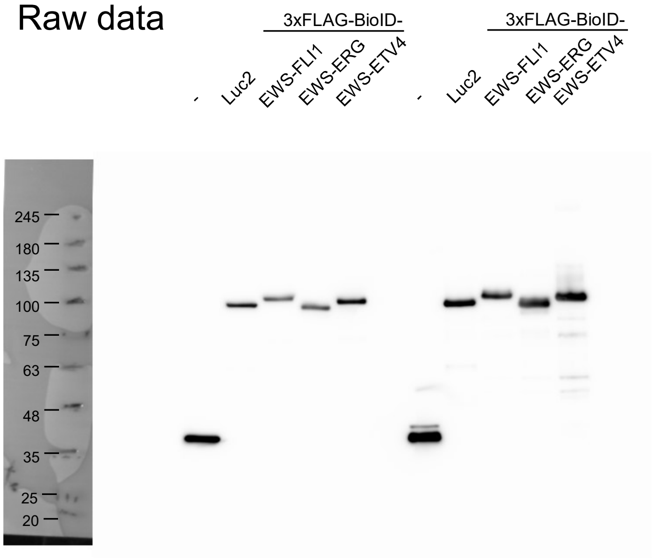

DYKDDDDK (FLAG) ab

Raw data

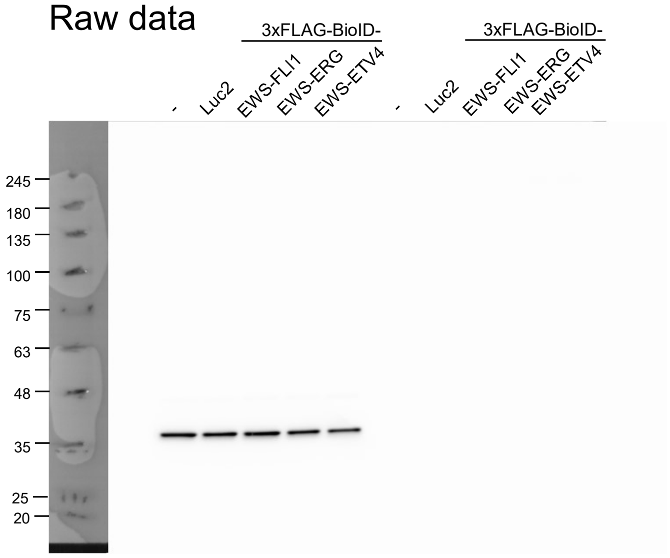

GAPDH ab

Fig 2B

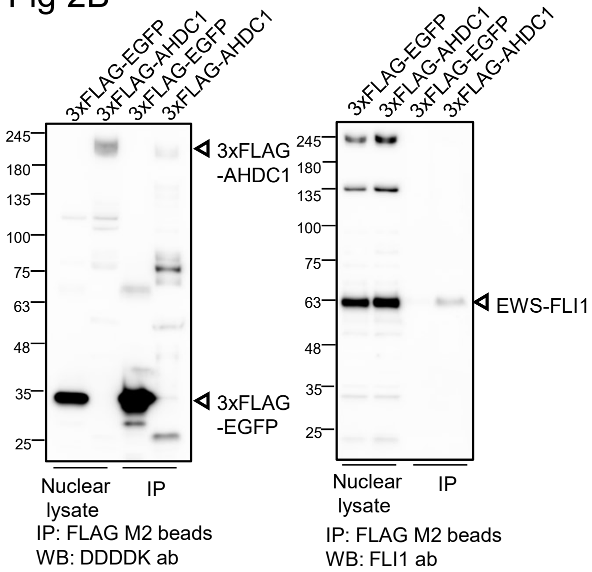

Raw data

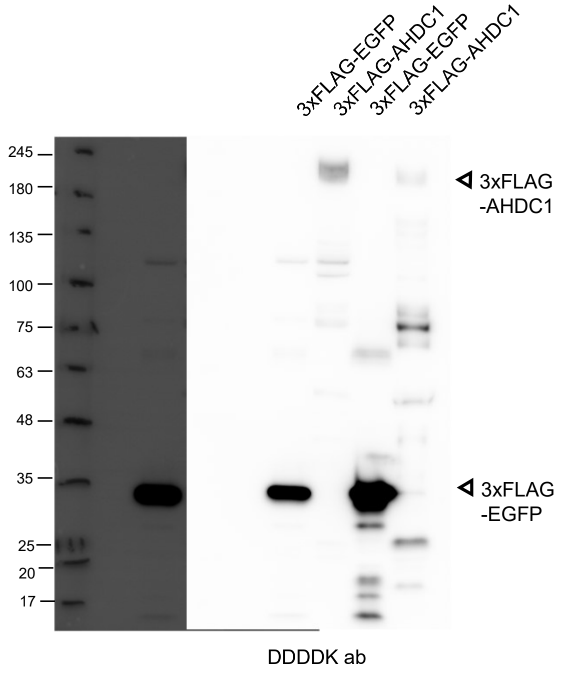

Raw data

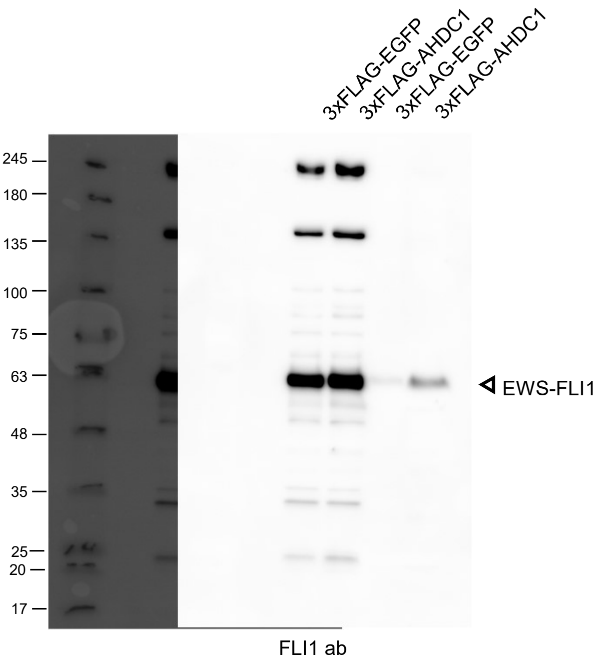

Fig 2C

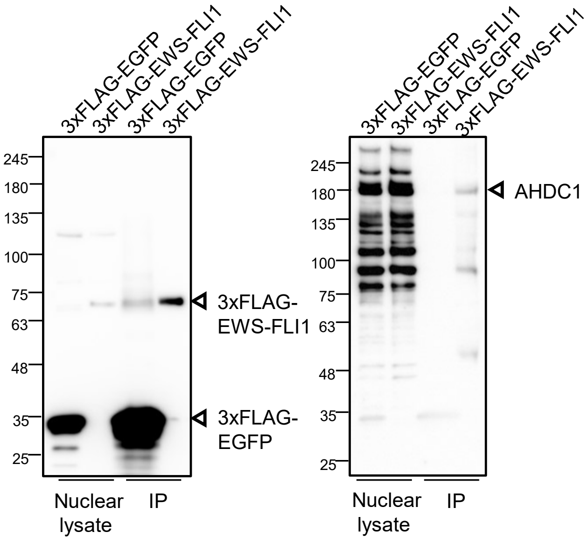

IP: FLAG M2 beads
WB: DDDDK ab

IP: FLAG M2 beads
WB: AHDC1 ab

Raw data

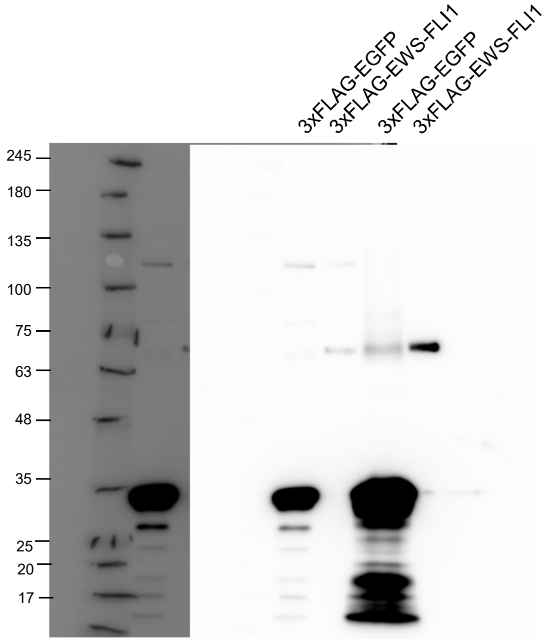

IP: FLAG M2 beads
WB: DDDDK ab

Raw data

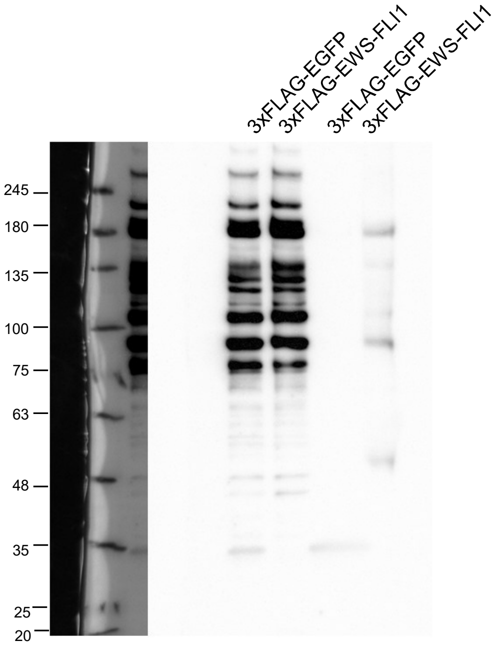

IP: FLAG M2 beads
WB: AHDC1 ab

Fig 2D

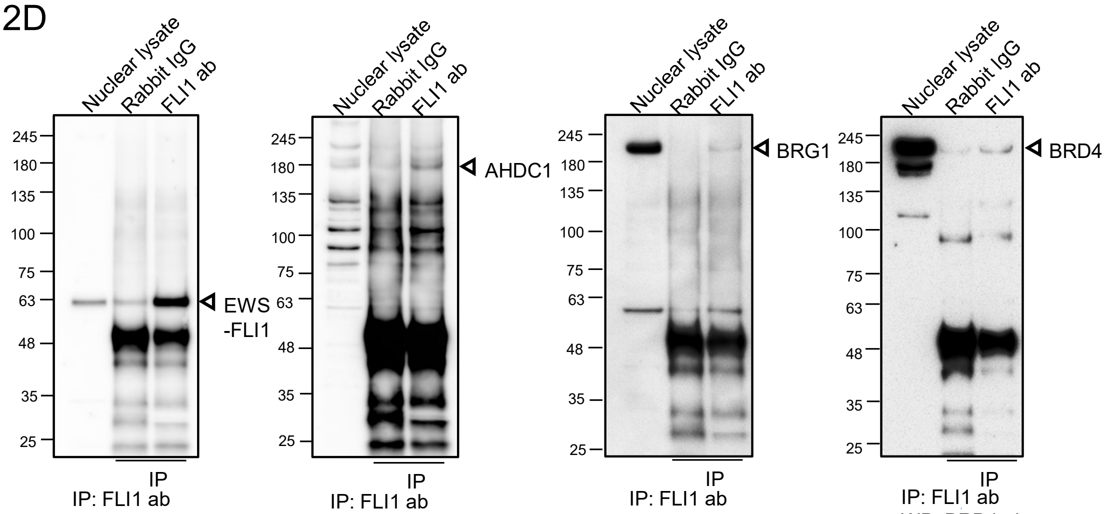

IP: FLI1 ab
WB: FLI1 ab

IP: FLI1 ab
WB: AHDC1 ab

IP: FLI1 ab
WB: BRG1 ab

IP: FLI1 ab
WB: BRD4 ab

Raw data

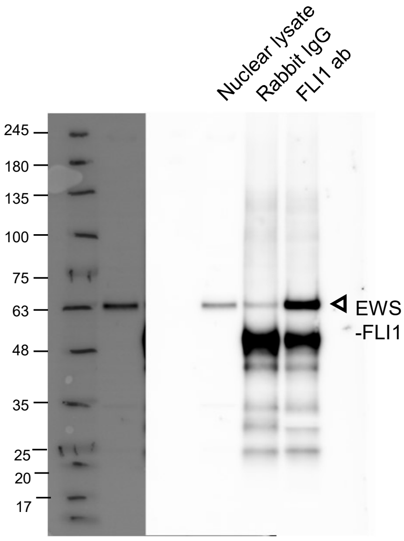

IP: FLI1 ab
WB: FLI1 ab

Raw data

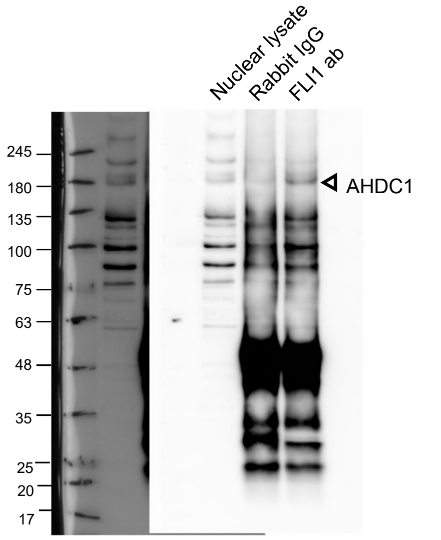

IP: FLI1 ab
WB: AHDC1 ab

Raw data

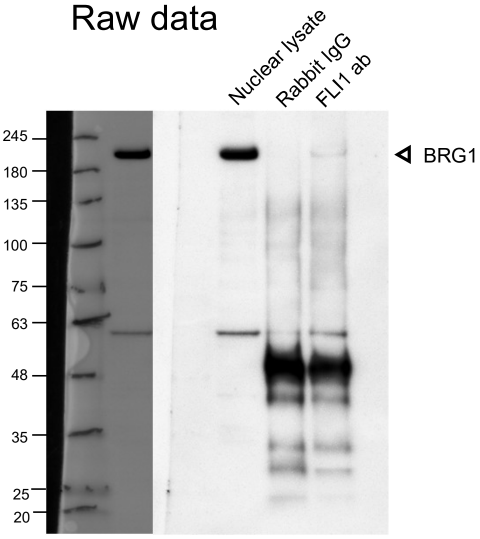

IP: FLI1 ab
WB: BRG1 ab

Raw data

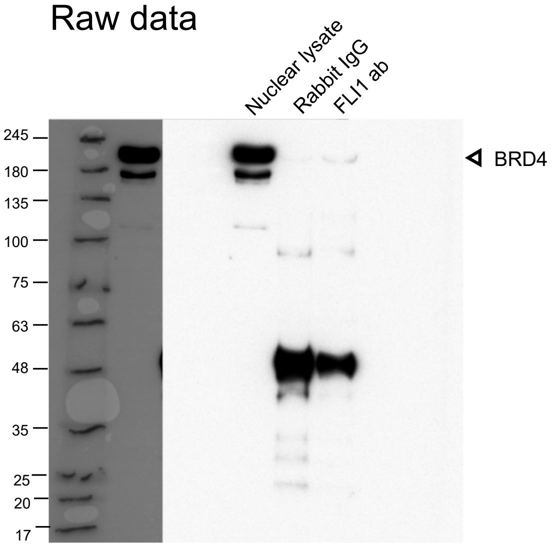

IP: FLI1 ab
WB: BRD4 ab

Fig 3A

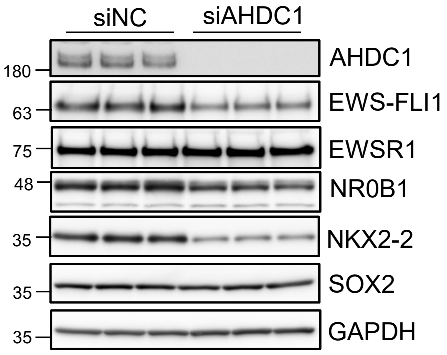

Raw data

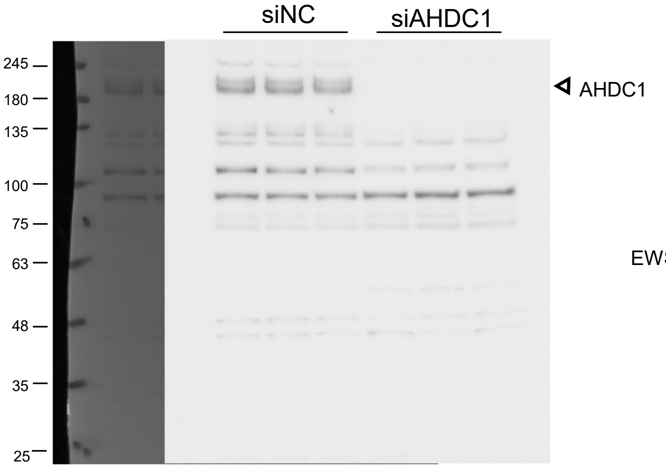

AHDC1 ab

Raw data

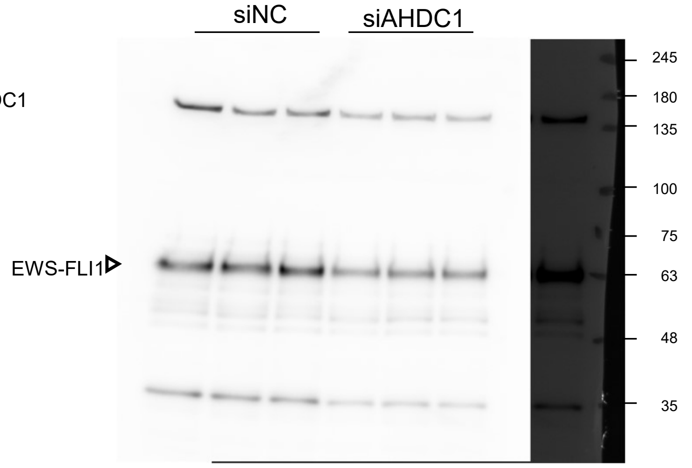

FLI1 ab

Raw data

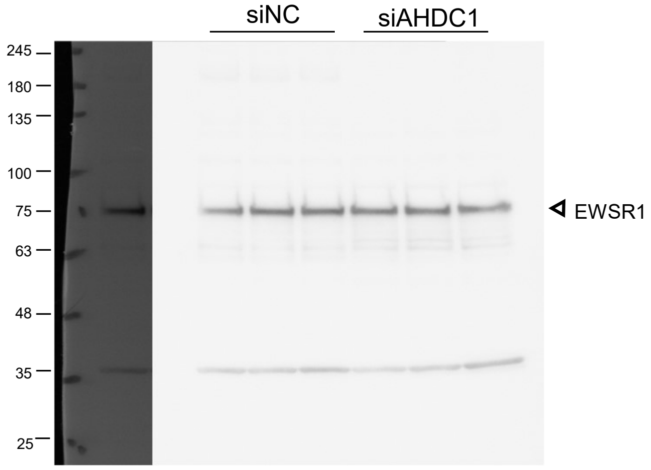

EWSR1 ab

Raw data

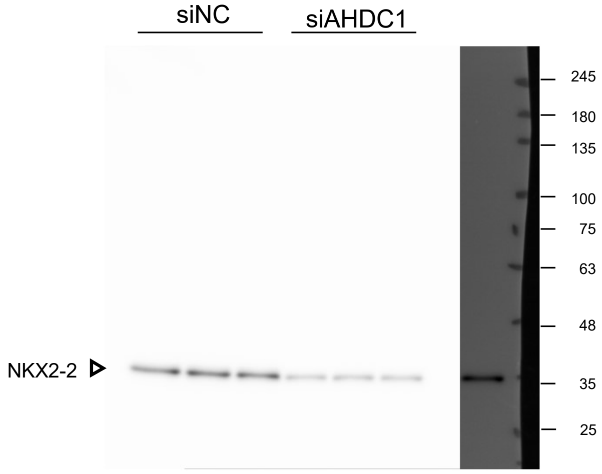

NKX2-2 ab

Fig 3A

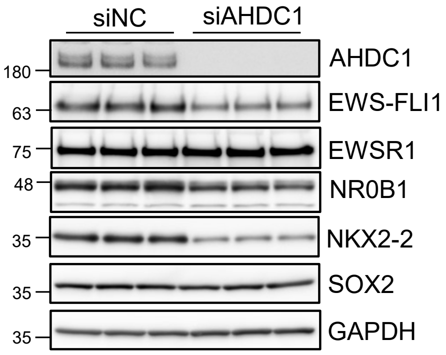

Raw data

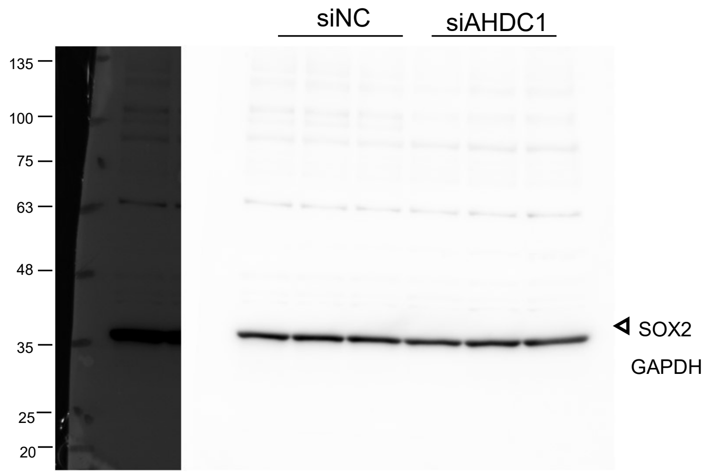

SOX2 ab

Raw data

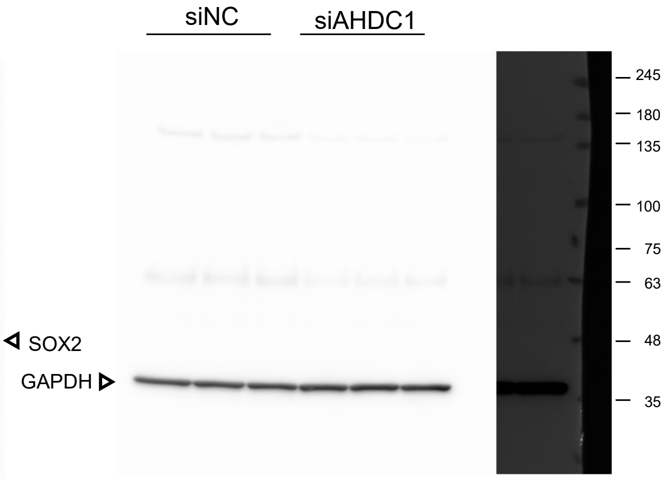

GAPDH ab

Raw data

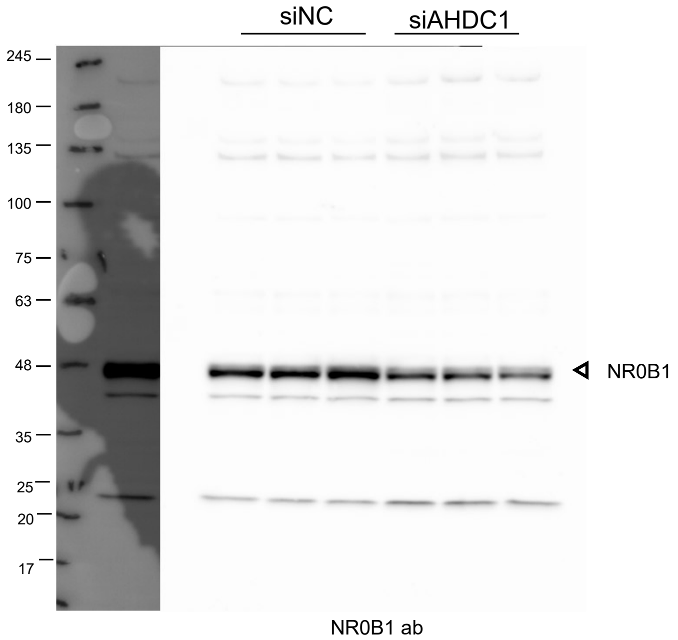

NR0B1 ab

Fig 3D

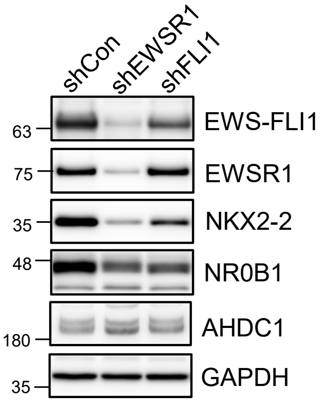
shCon
shEWSR1
shFLI1
63
EWS-FLI1
75
EWSR1
35
NKX2-2
48
NR0B1
180
AHDC1
35
GAPDH

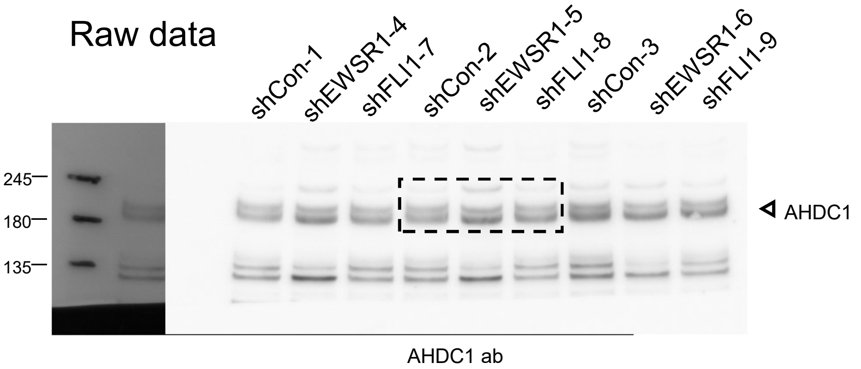
Raw data
shCon-1
shEWSR1-4
shFLI1-7
shCon-2
shEWSR1-5
shFLI1-8
shCon-3
shEWSR1-6
shFLI1-9
245
180
135
AHDC1
AHDC1 ab

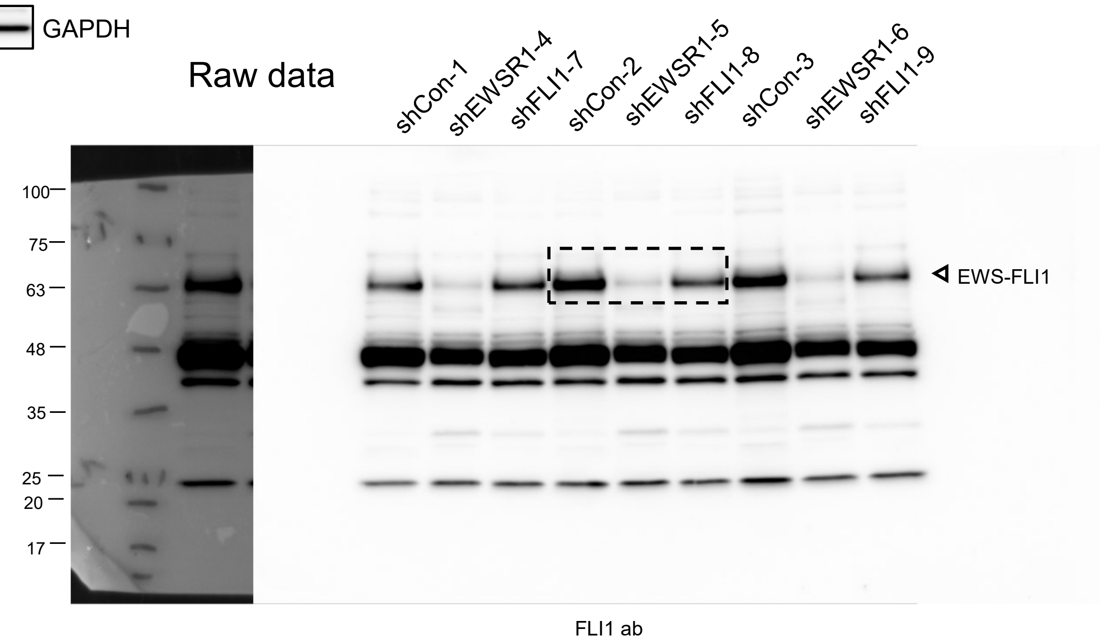
Raw data
shCon-1
shEWSR1-4
shFLI1-7
shCon-2
shEWSR1-5
shFLI1-8
shCon-3
shEWSR1-6
shFLI1-9
100
75
63
48
35
25
20
17
EWS-FLI1
FLI1 ab

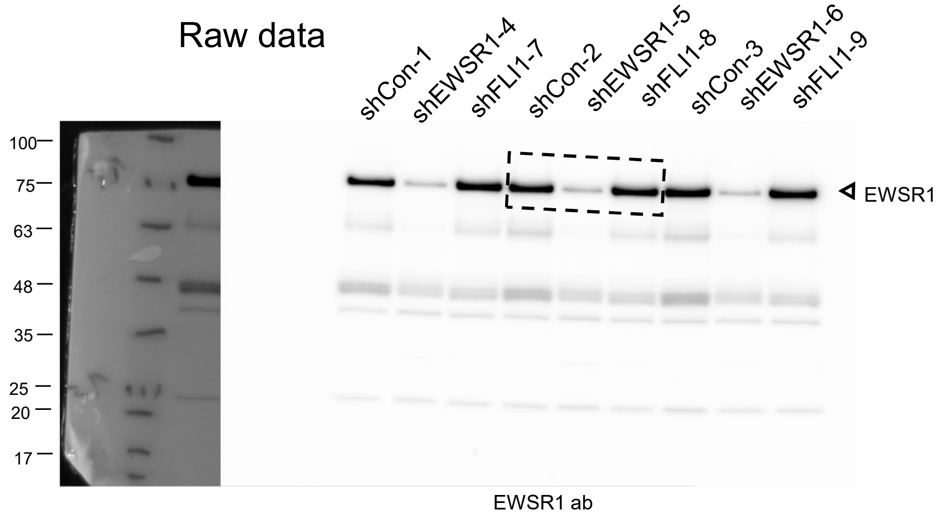
Raw data
shCon-1
shEWSR1-4
shFLI1-7
shCon-2
shEWSR1-5
shFLI1-8
shCon-3
shEWSR1-6
shFLI1-9
100
75
63
48
35
25
20
17
EWSR1
EWSR1 ab

Fig 3D

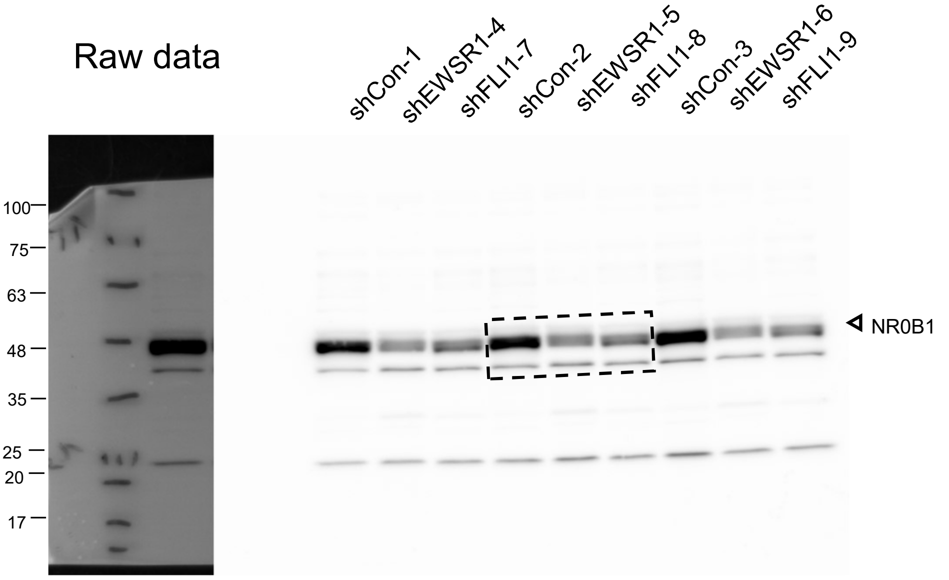

NR0B1 ab

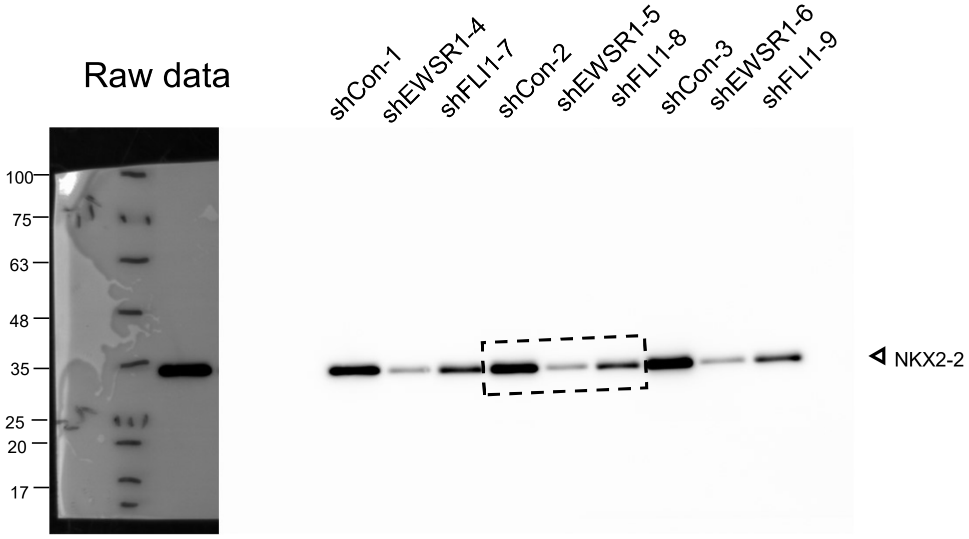

NKX2-2 ab

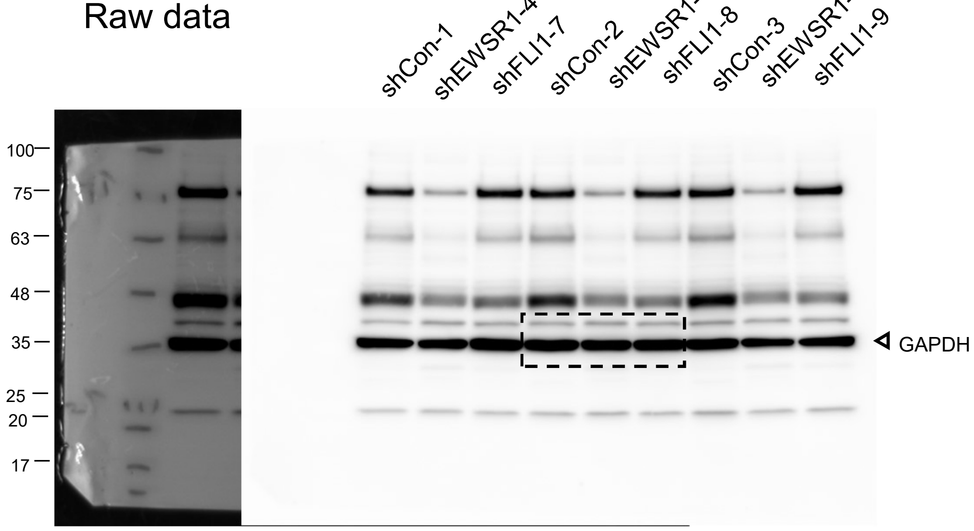

GAPDH ab

Fig 4C

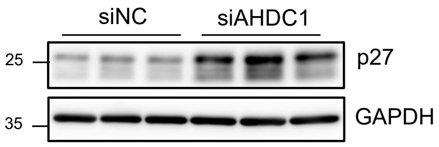

Raw data

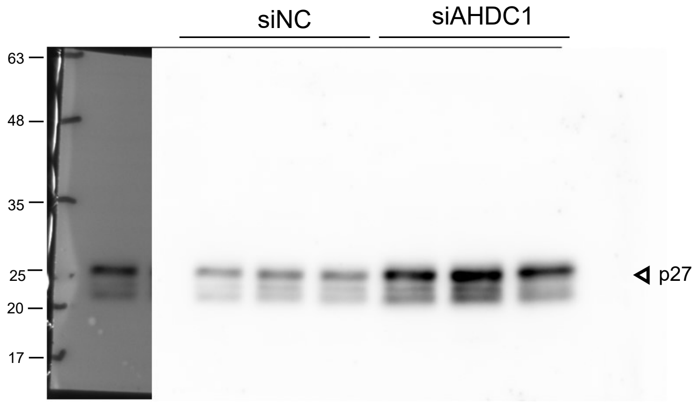

p27 ab

Raw data

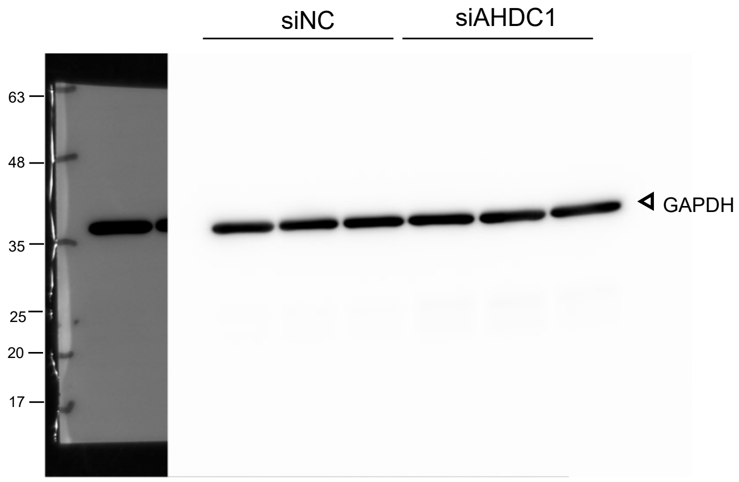

GAPDH ab

Fig 4E

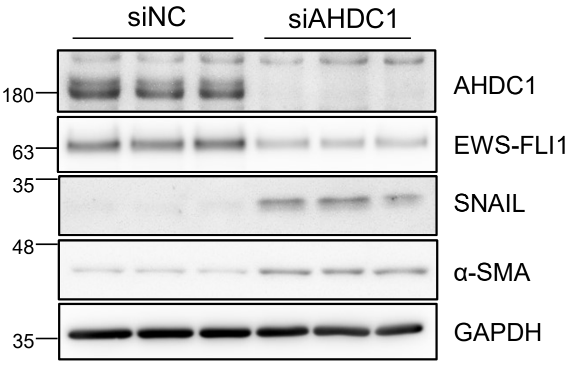

Raw data

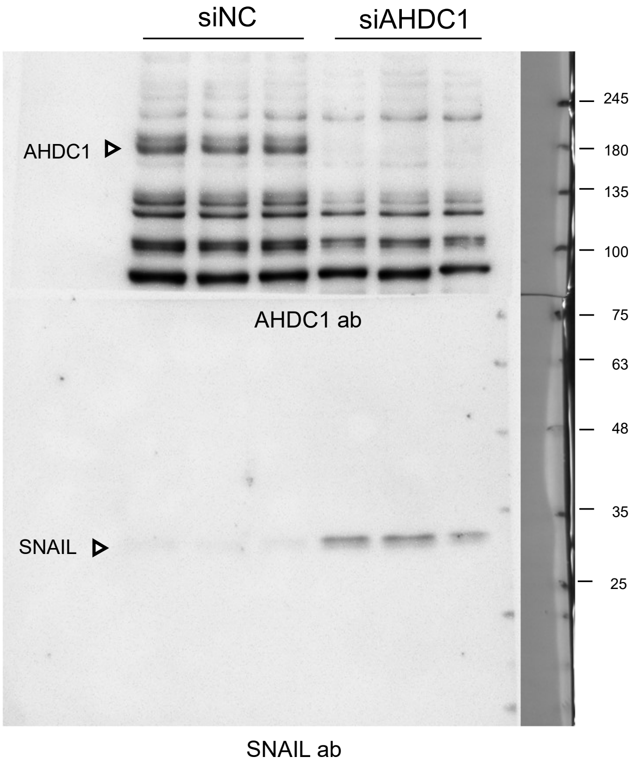

Raw data

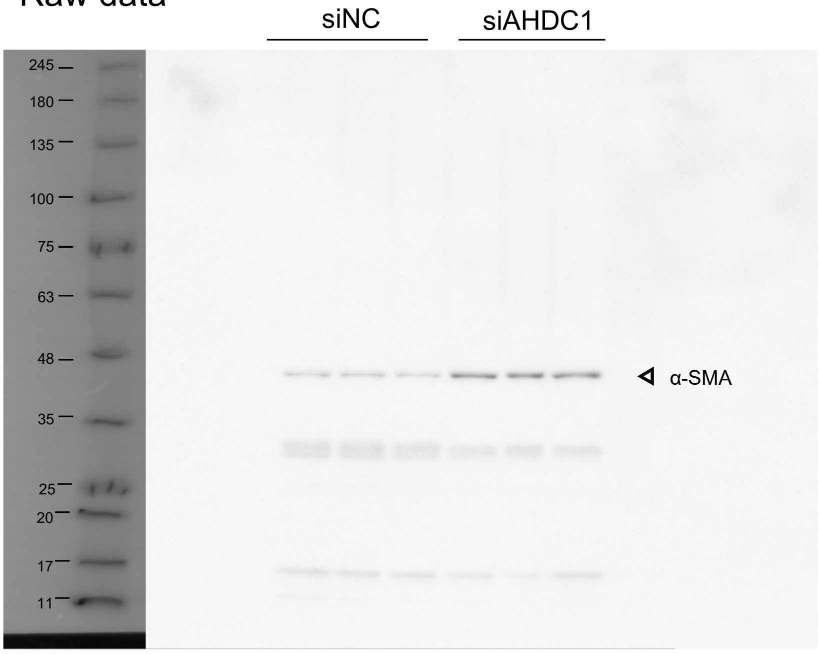

Fig 4E

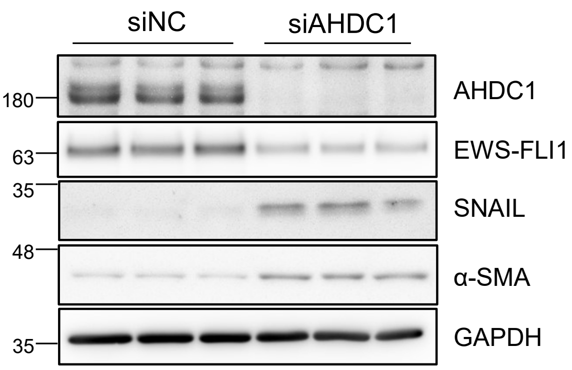

Raw data

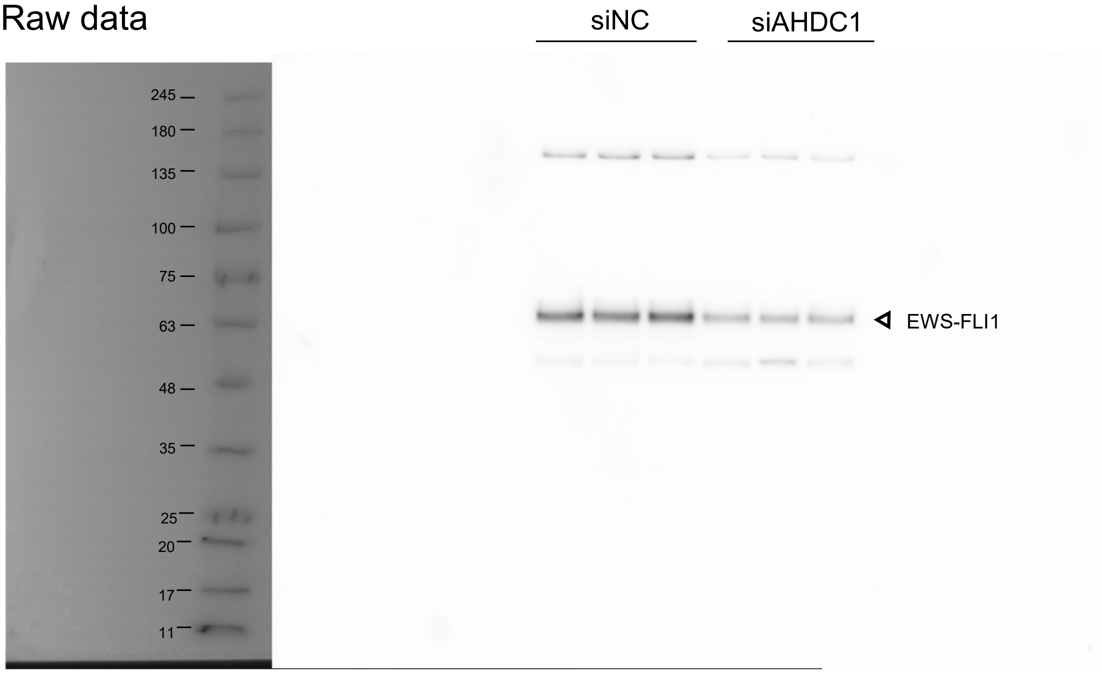

FLI1 ab

Raw data

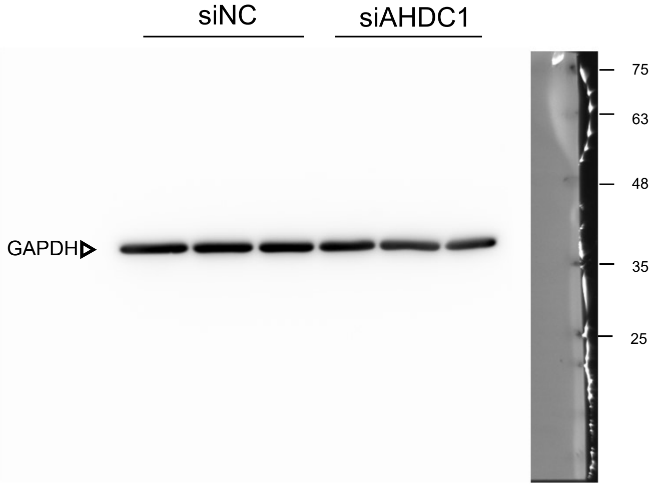

GAPDH ab

Fig 5A

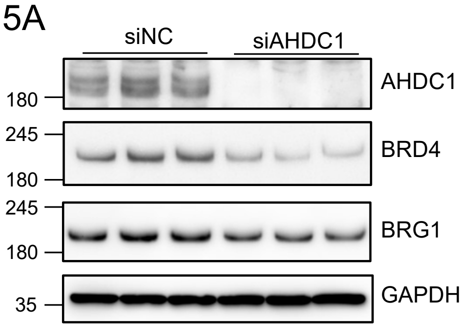

Raw data

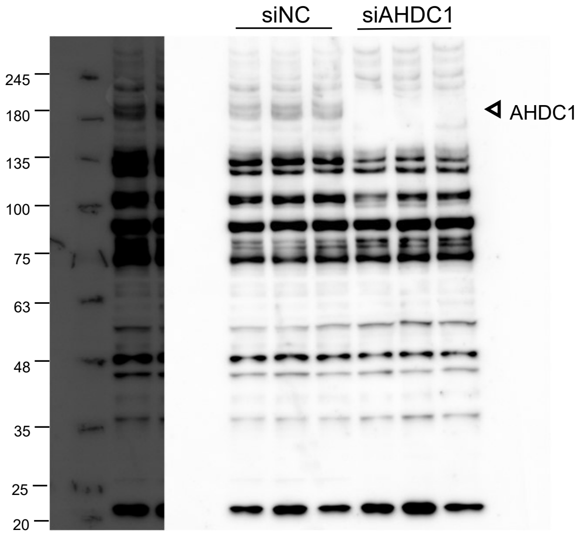

AHDC1 ab

Raw data

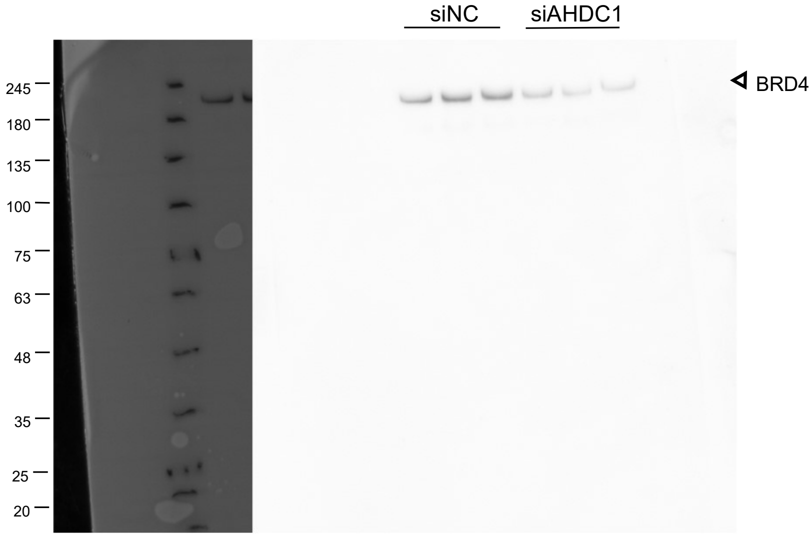

BRD4 ab

Raw data

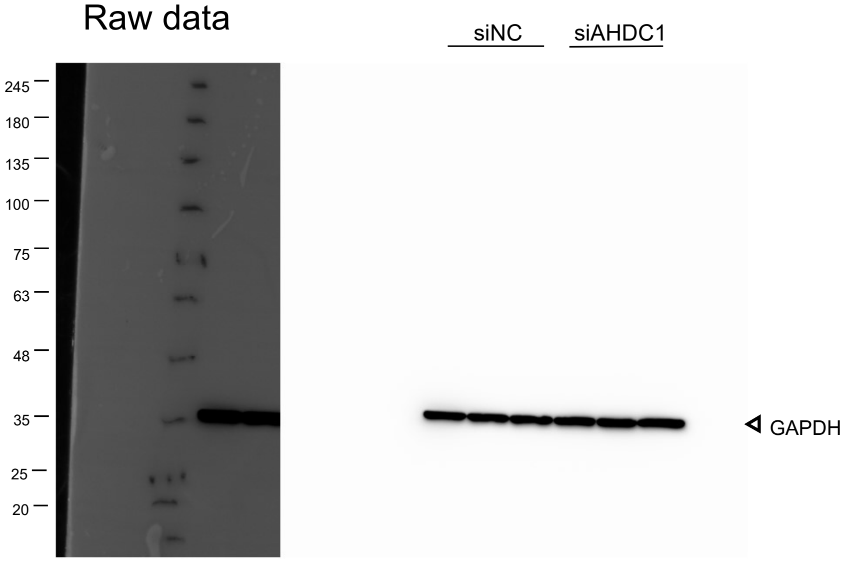

GAPDH ab

Fig 5A

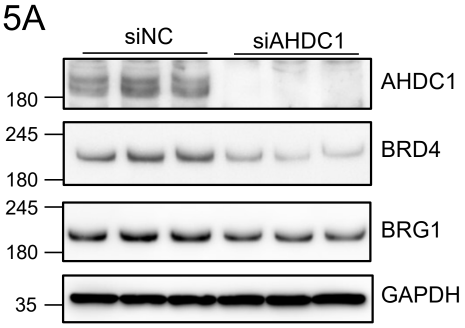

Raw data

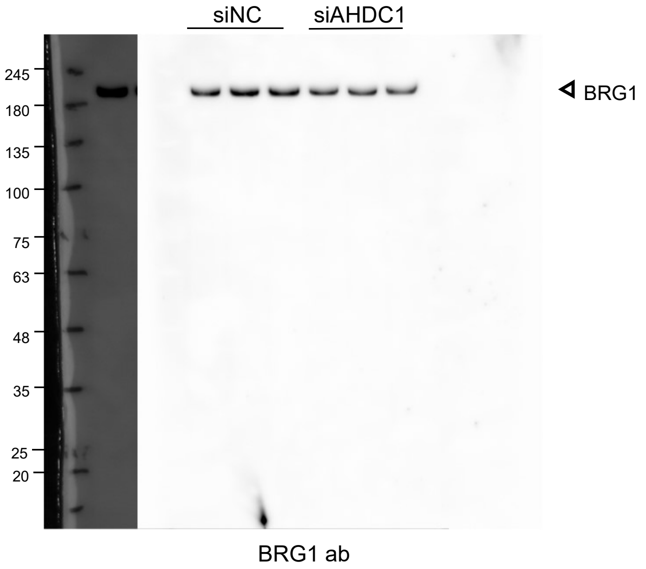

Fig S1A

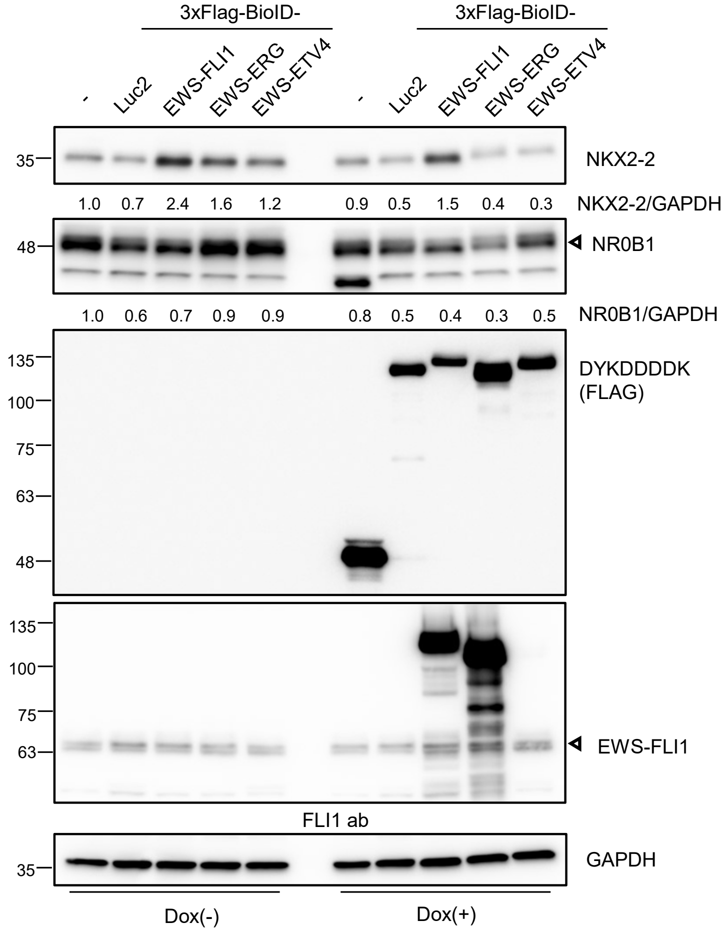

Raw data

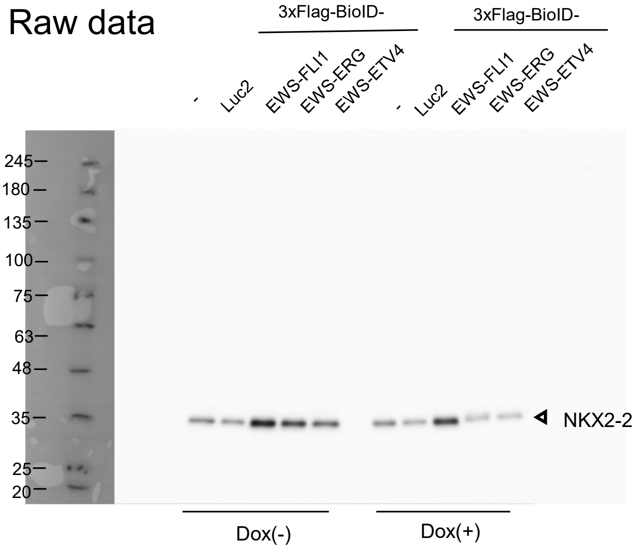

Fig S1A

Raw data

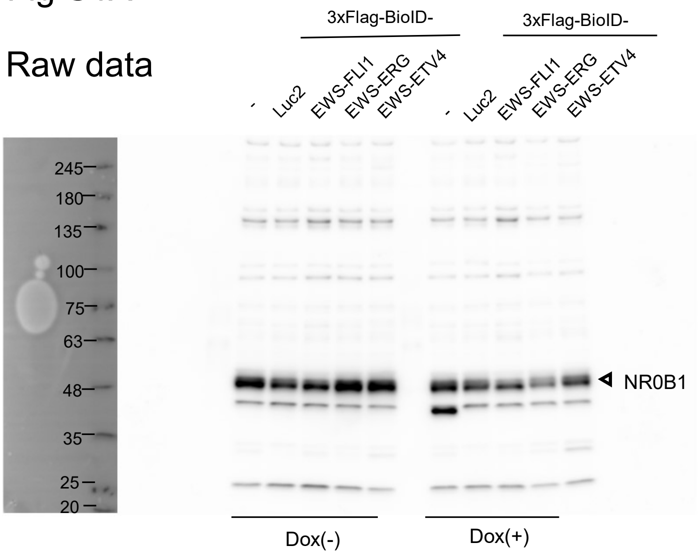

NR0B1 ab

Raw data

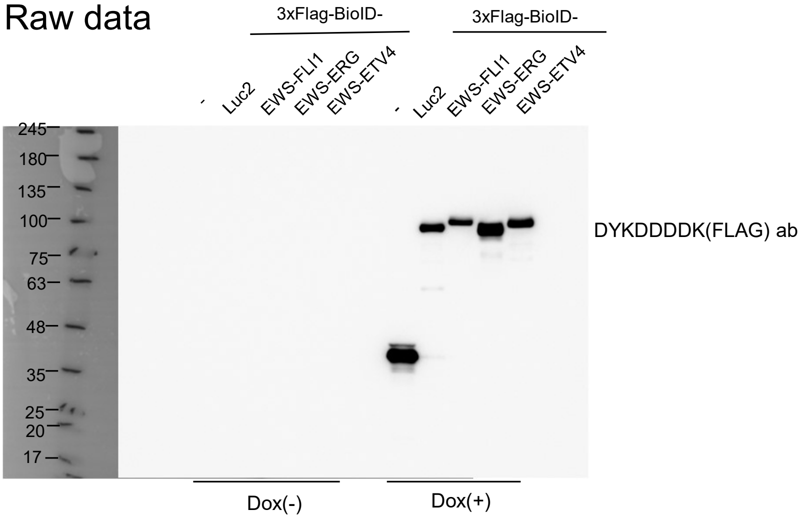

Raw data

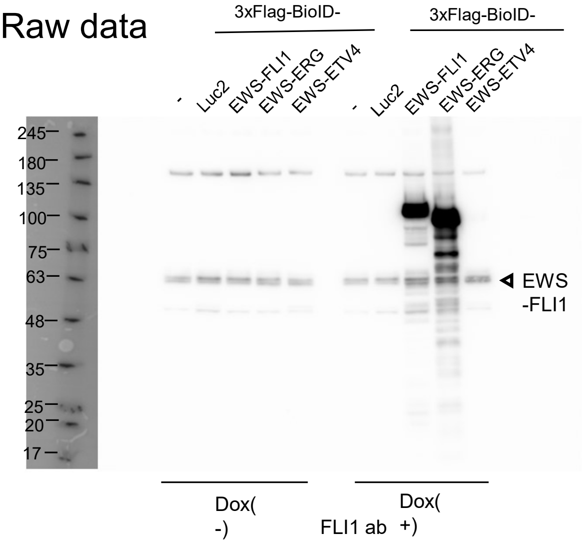

FLI1 ab

Raw data

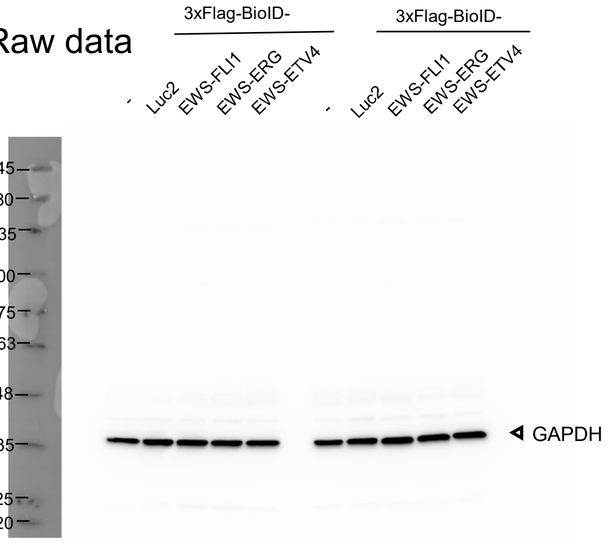

GAPDH ab

Fig S3

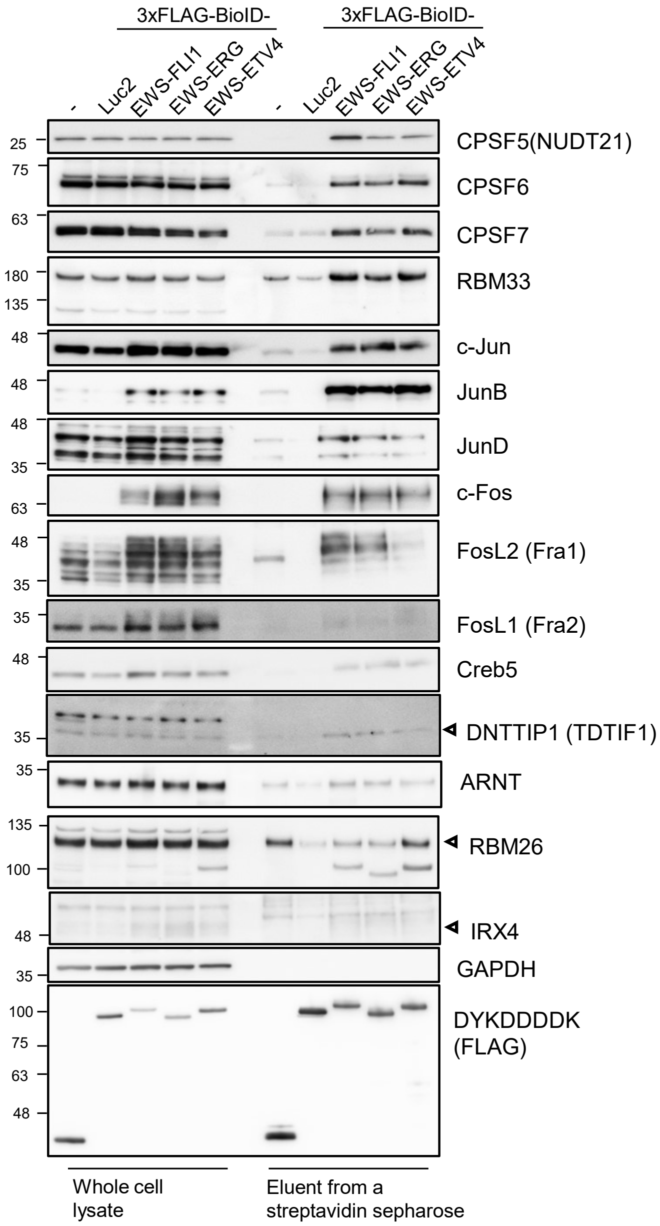
3xFLAG-BioID-
3xFLAG-BioID-
-
Luc2
EWS-FLI1
EWS-ERG
EWS-ETV4
-
Luc2
EWS-FLI1
EWS-ERG
EWS-ETV4
25
75
63
180
135
48
48
48
35
63
48
35
35
48
35
35
135
100
48
35
100
75
63
48
CPSF5(NUDT21)
CPSF6
CPSF7
RBM33
c-Jun
JunB
JunD
c-Fos
FosL2 (Fra1)
FosL1 (Fra2)
Creb5
DNTTIP1 (TDTIF1)
ARNT
RBM26
IRX4
GAPDH
DYKDDDDK
(FLAG)
Whole cell
lysate
Eluent from a
streptavidin sepharose

Fig S3

Raw data

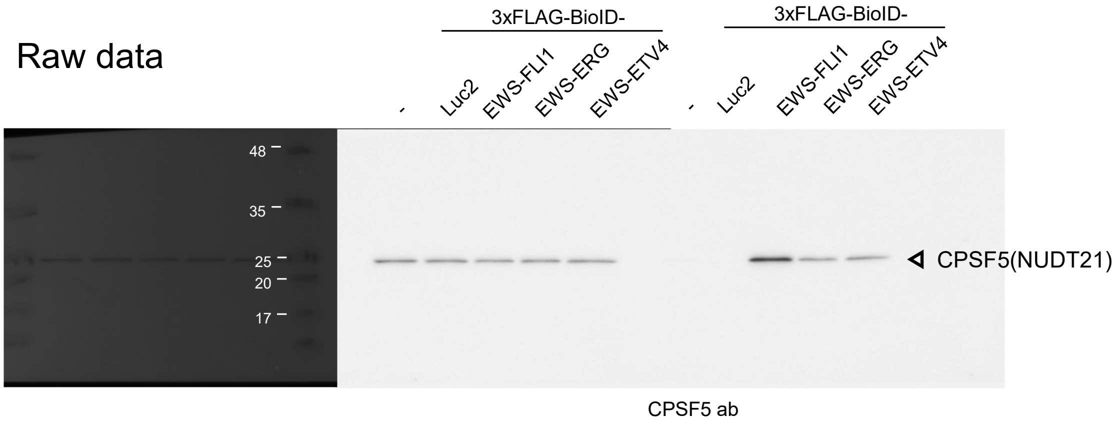

Raw data

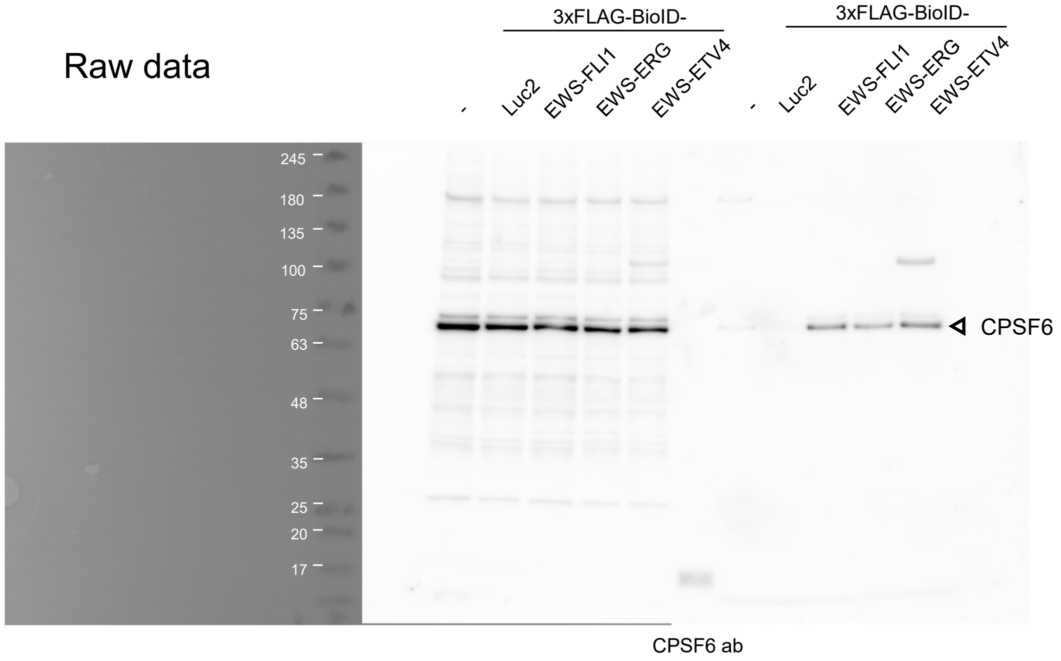

Raw data

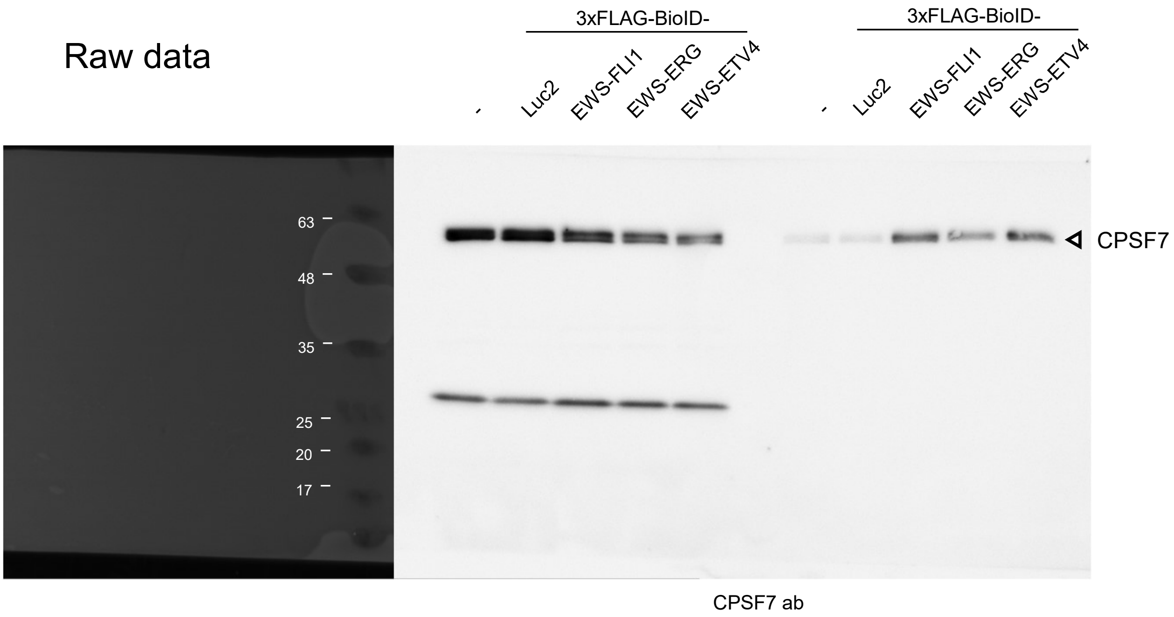

Fig S3

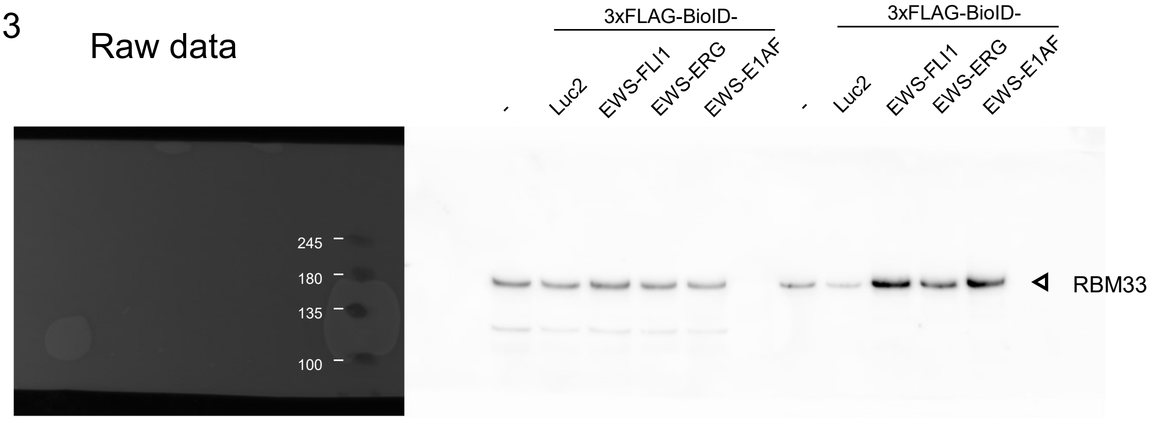

RBM33 ab

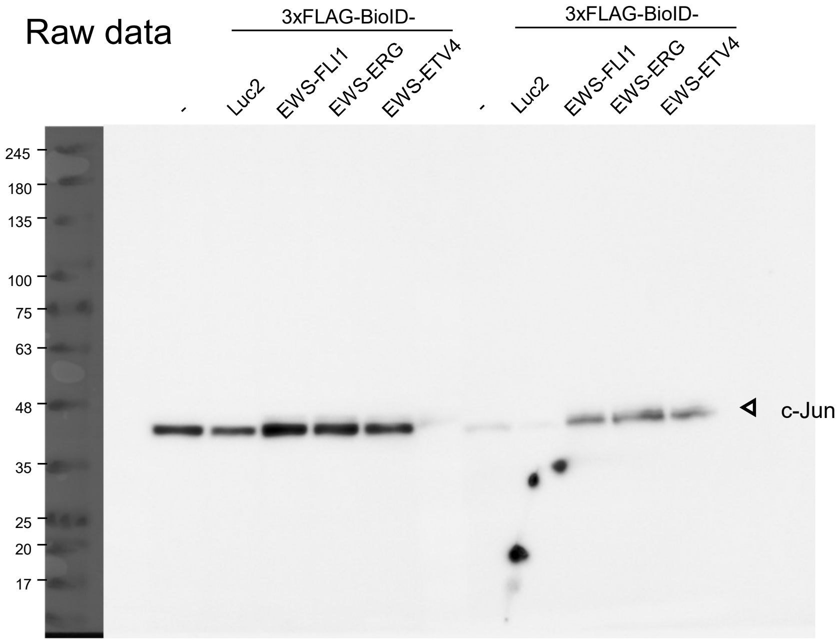

c-Jun ab

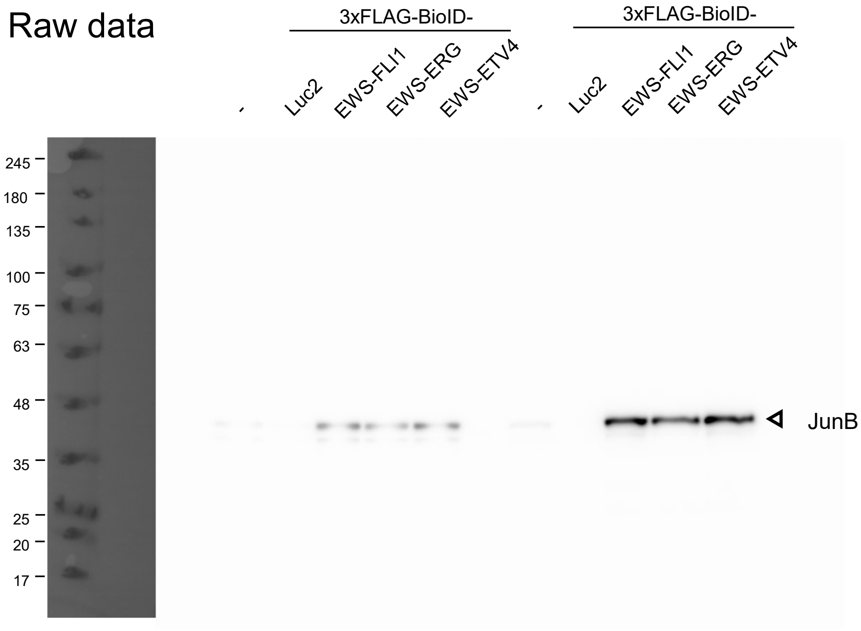

JunB ab

Fig S3

Raw data

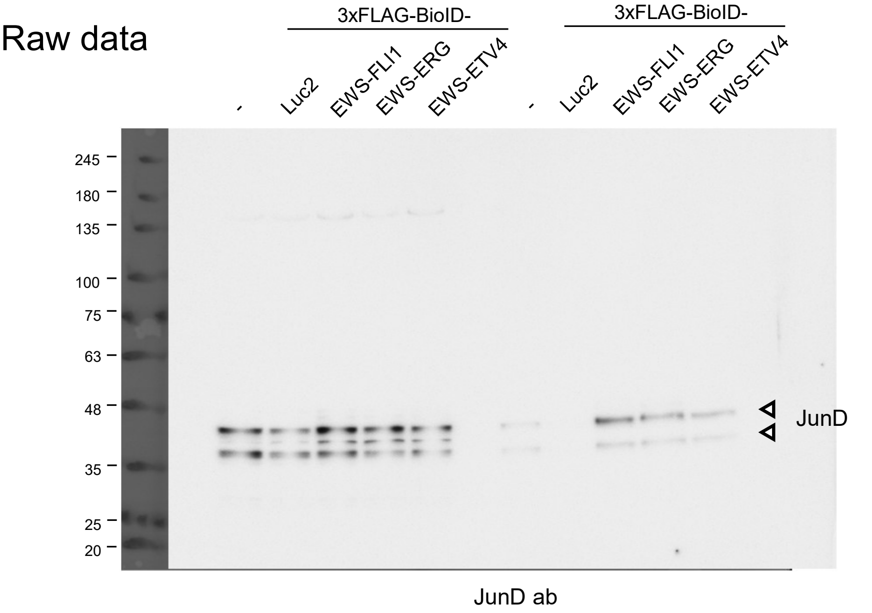

Raw data

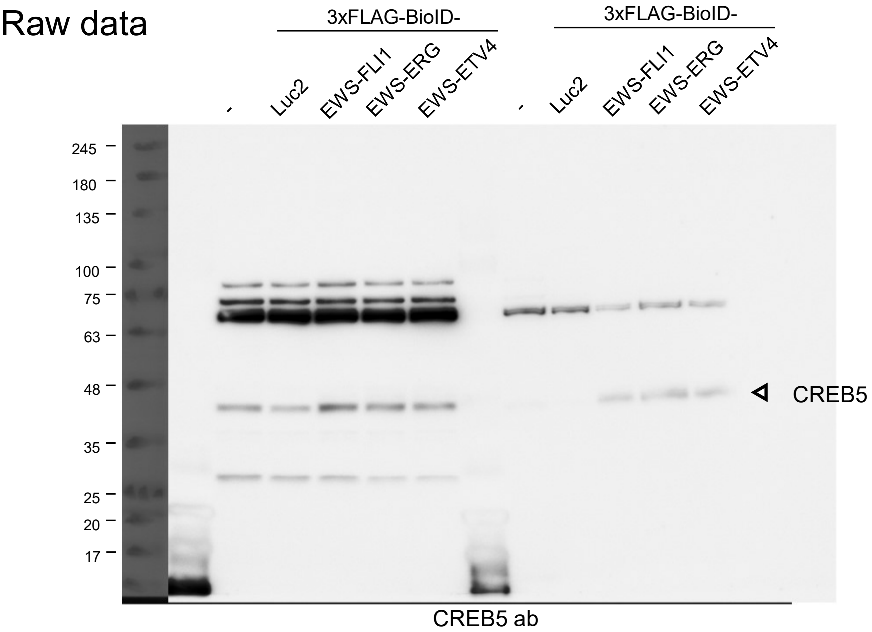

Raw data

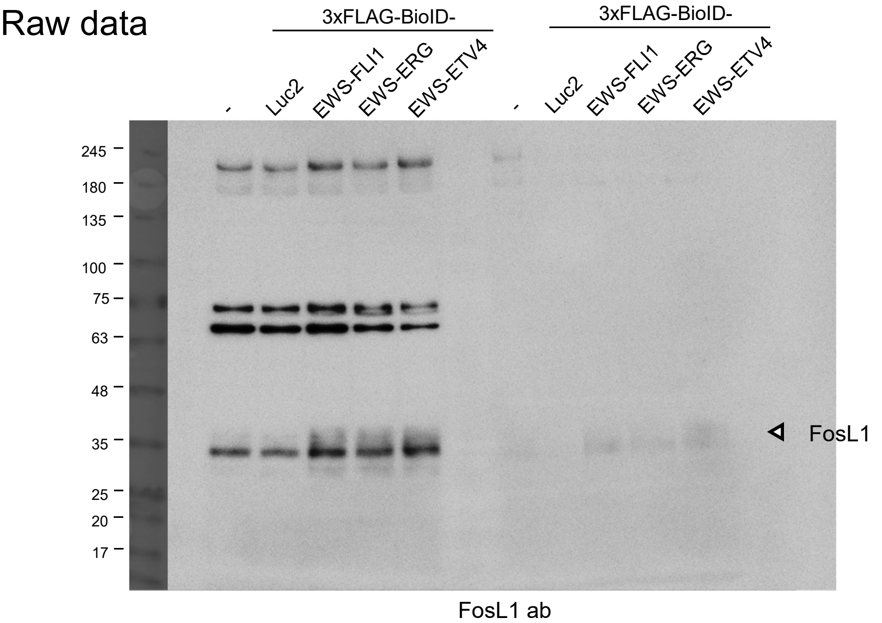

Fig S3

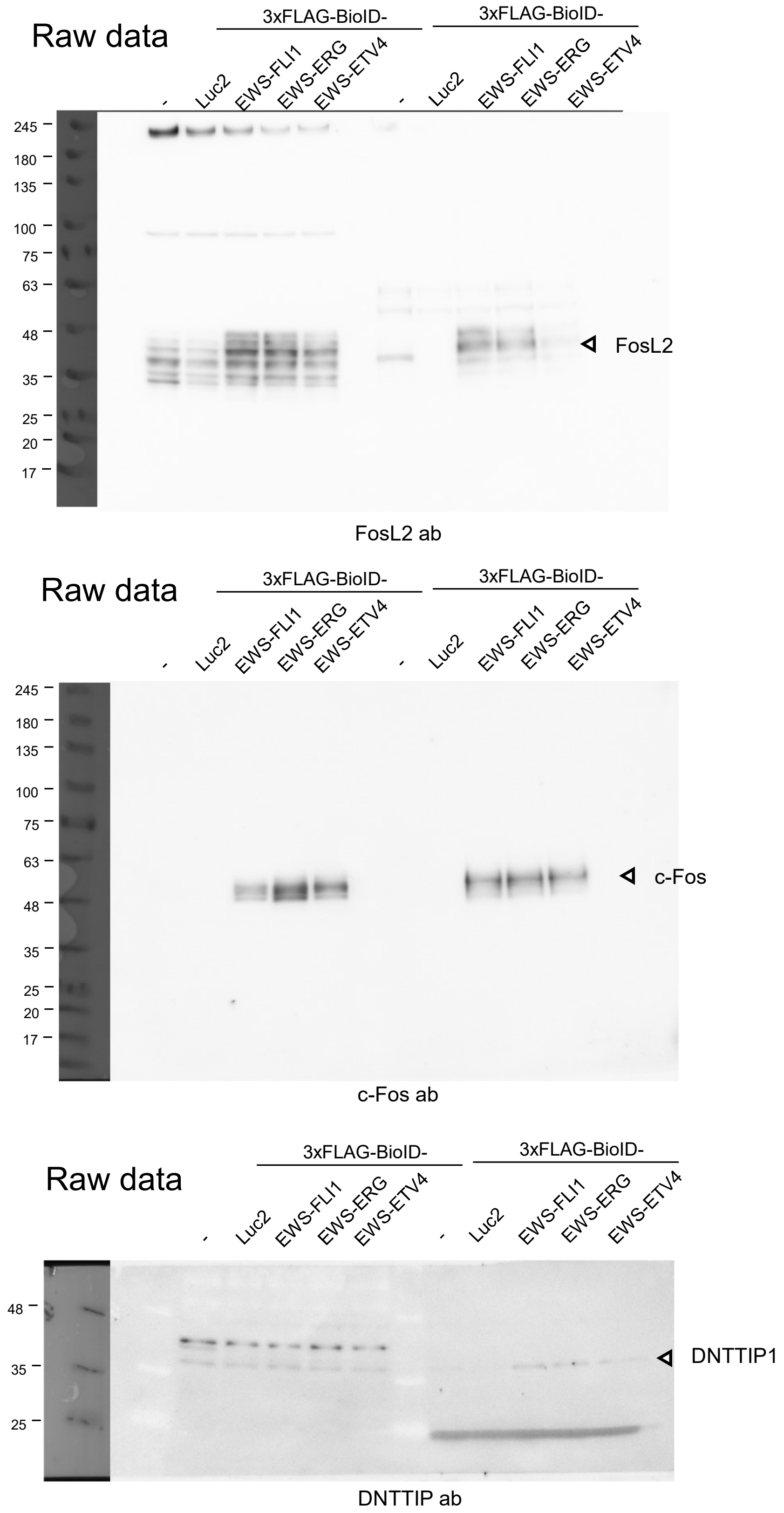
Raw data
3xFLAG-BioID-
3xFLAG-BioID-
-
Luc2
EWS-FLI1
EWS-ERG
EWS-ETV4
-
Luc2
EWS-FLI1
EWS-ERG
EWS-ETV4
245
180
135
100
75
63
48
35
25
20
17
FosL2
FosL2 ab
Raw data
3xFLAG-BioID-
3xFLAG-BioID-
-
Luc2
EWS-FLI1
EWS-ERG
EWS-ETV4
-
Luc2
EWS-FLI1
EWS-ERG
EWS-ETV4
245
180
135
100
75
63
48
35
25
20
17
c-Fos
c-Fos ab
Raw data
3xFLAG-BioID-
3xFLAG-BioID-
-
Luc2
EWS-FLI1
EWS-ERG
EWS-ETV4
-
Luc2
EWS-FLI1
EWS-ERG
EWS-ETV4
48
35
25
DNTTIP1
DNTTIP ab

Fig S3

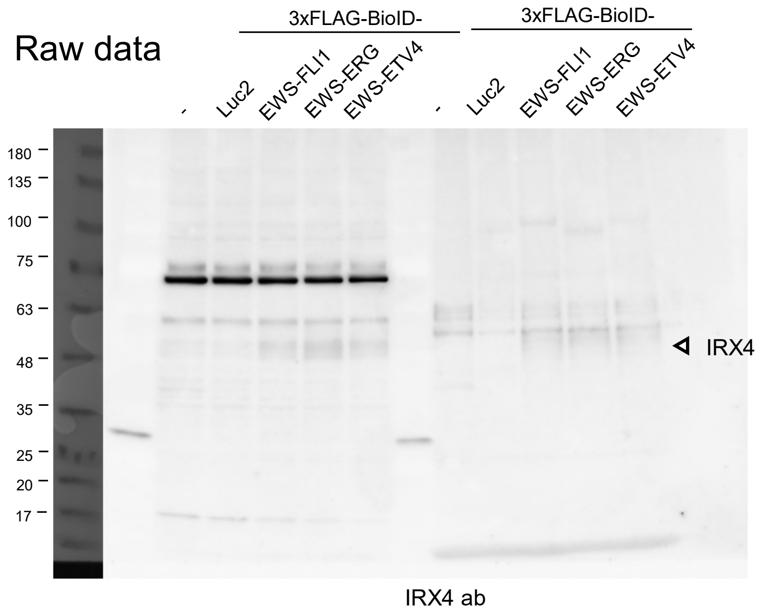
Raw data
3xFLAG-BioID-
3xFLAG-BioID-
-
Luc2
EWS-FLI1
EWS-ERG
EWS-ETV4
-
Luc2
EWS-FLI1
EWS-ERG
EWS-ETV4
180
135
100
75
63
48
35
25
20
17
IRX4
IRX4 ab

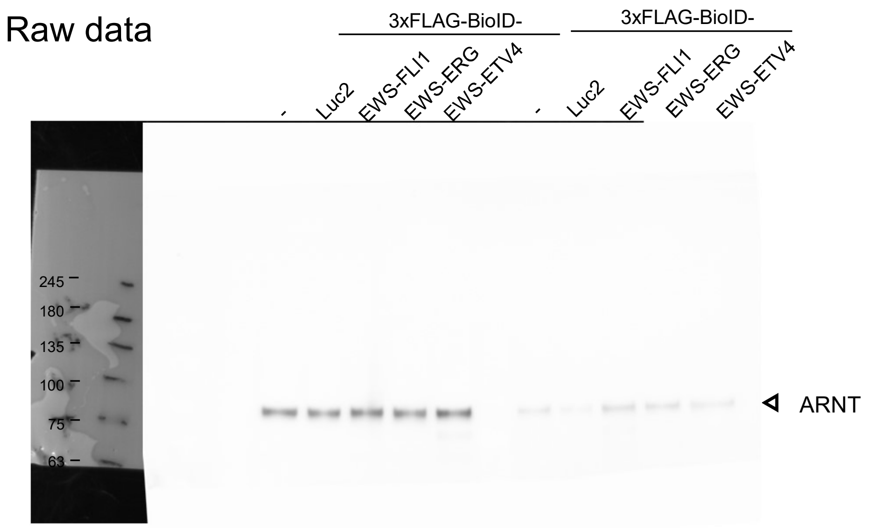
Raw data
3xFLAG-BioID-
3xFLAG-BioID-
-
Luc2
EWS-FLI1
EWS-ERG
EWS-ETV4
-
Luc2
EWS-FLI1
EWS-ERG
EWS-ETV4
245
180
135
100
75
63
ARNT

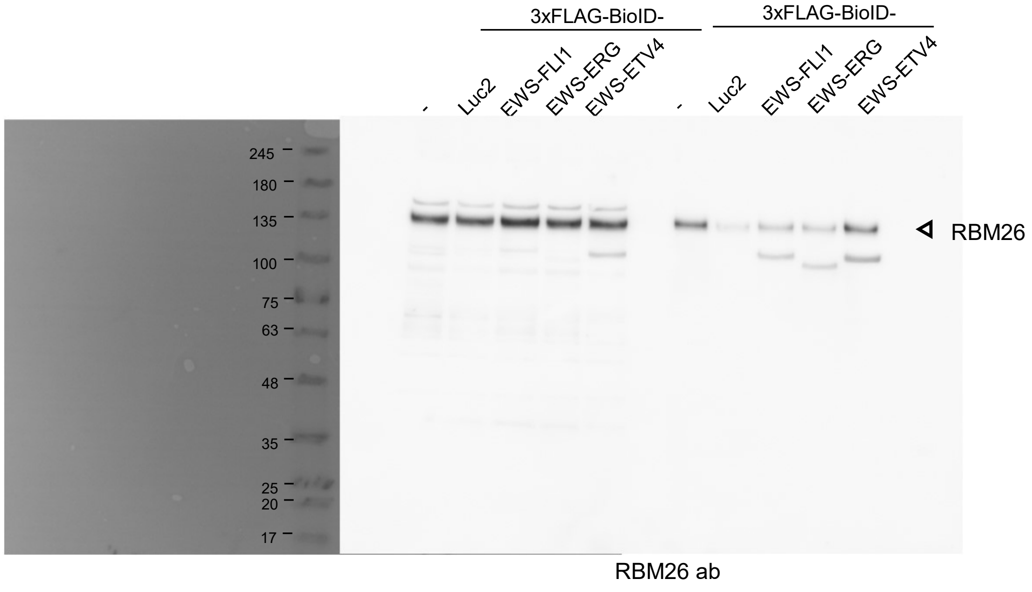
3xFLAG-BioID-
3xFLAG-BioID-
-
Luc2
EWS-FLI1
EWS-ERG
EWS-ETV4
-
Luc2
EWS-FLI1
EWS-ERG
EWS-ETV4
245
180
135
100
75
63
48
35
25
20
17
RBM26
RBM26 ab

Fig S3

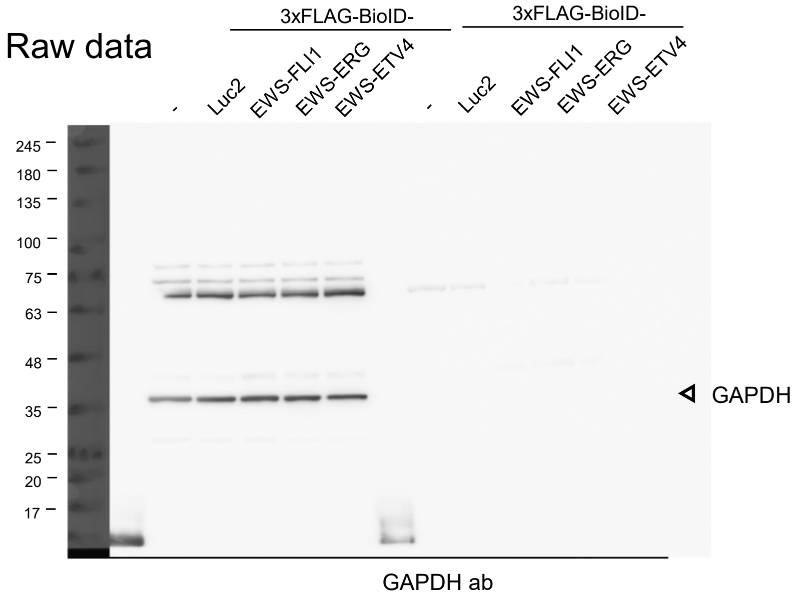
Raw data
3xFLAG-BioID-
3xFLAG-BioID-
-
Luc2
EWS-FLI1
EWS-ERG
EWS-ETV4
-
Luc2
EWS-FLI1
EWS-ERG
EWS-ETV4
245
180
135
100
75
63
48
35
25
20
17
GAPDH
GAPDH ab

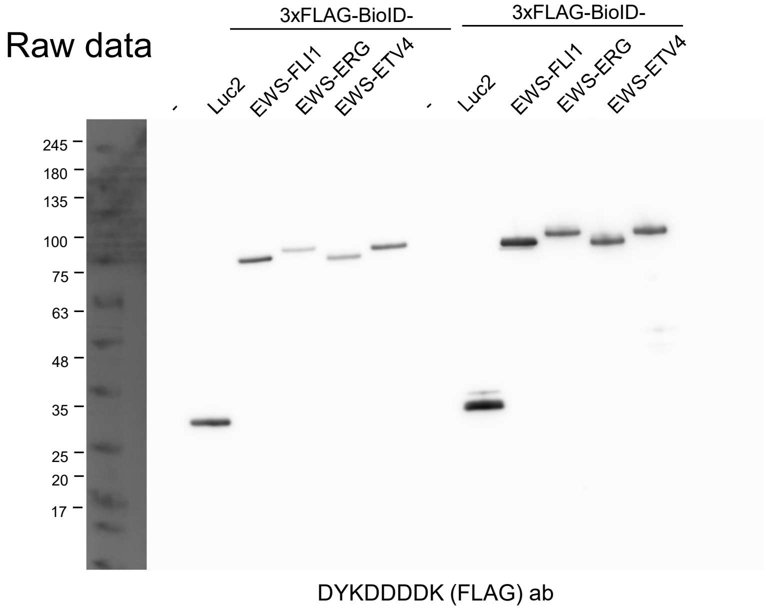
Raw data
3xFLAG-BioID-
3xFLAG-BioID-
-
Luc2
EWS-FLI1
EWS-ERG
EWS-ETV4
-
Luc2
EWS-FLI1
EWS-ERG
EWS-ETV4
245
180
135
100
75
63
48
35
25
20
17
DYKDDDDK (FLAG) ab

Fig S4B

A

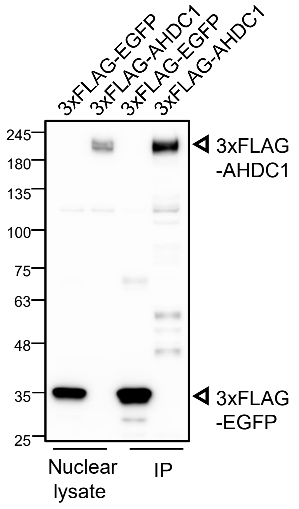

IP: DDDDK beads
WB: DDDDK ab

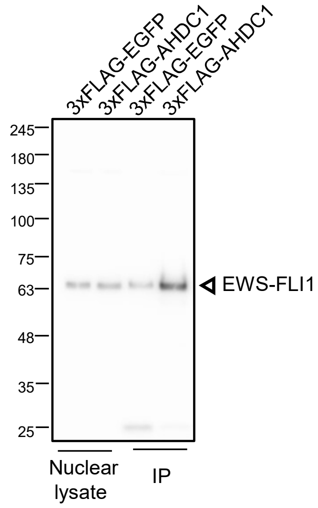

IP: DDDDK beads
WB: FLI1 ab

Raw data

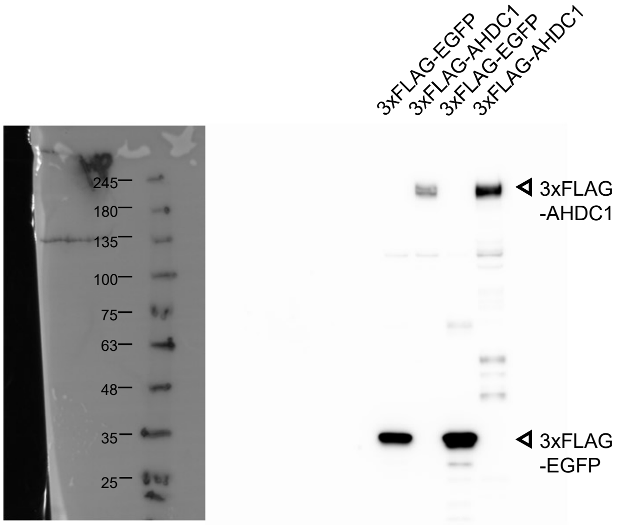

DDDDK ab

Raw data

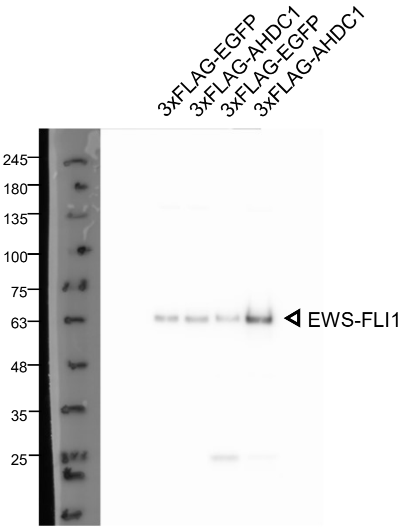

FLI1 ab

Fig S4C

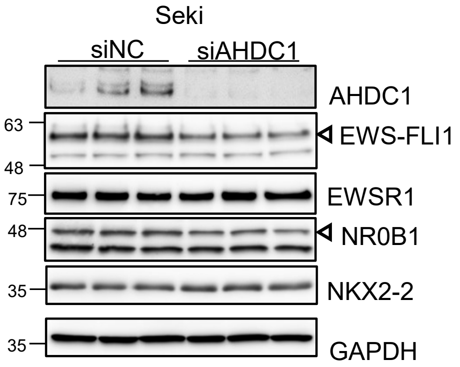

Raw data

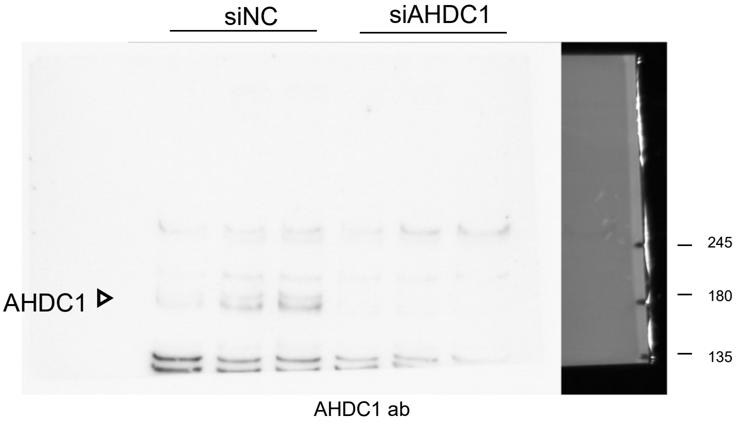

Raw data

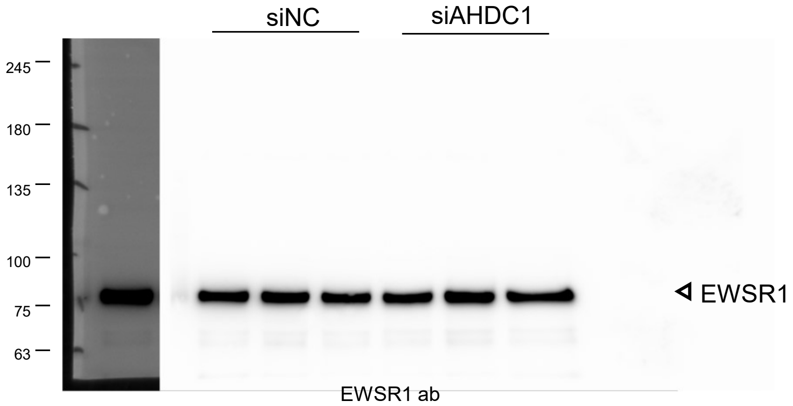

Raw data

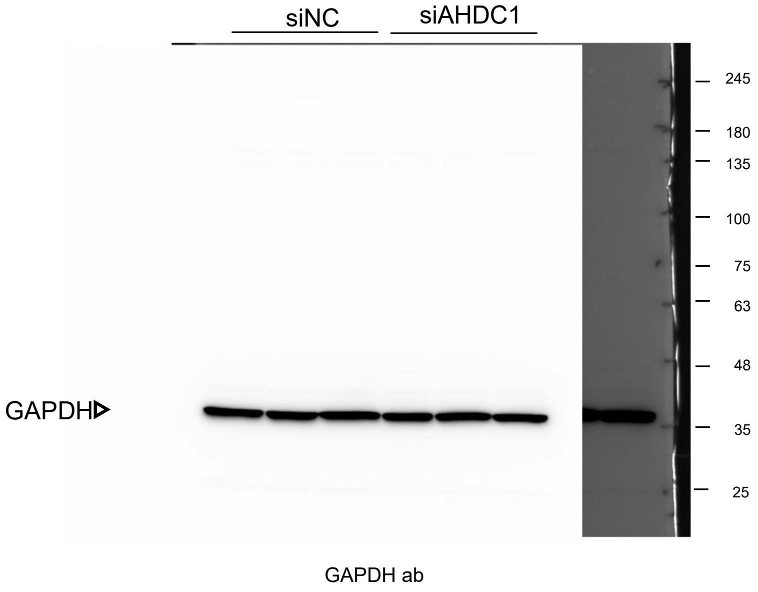

Fig S4C

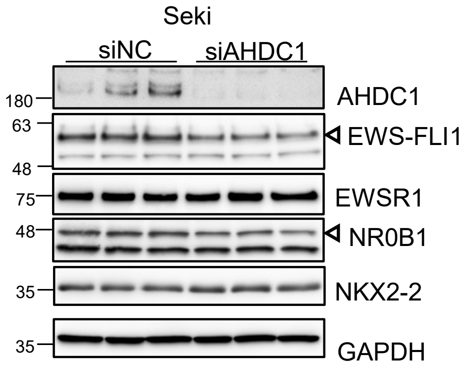

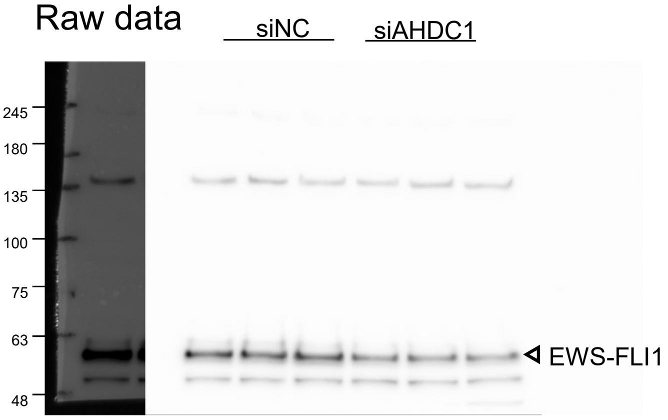

FLI1 ab

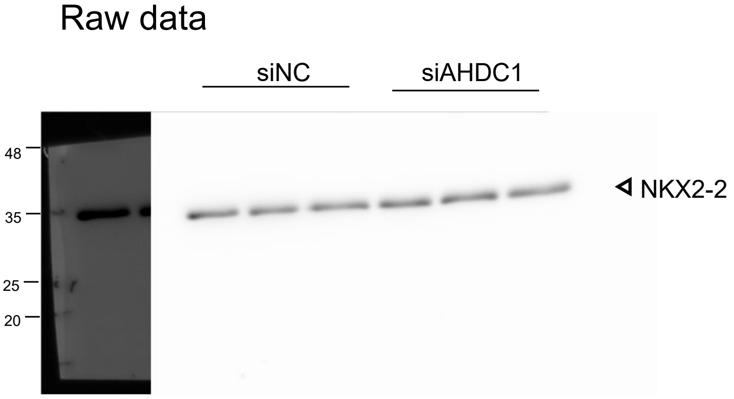

NKX2-2 ab

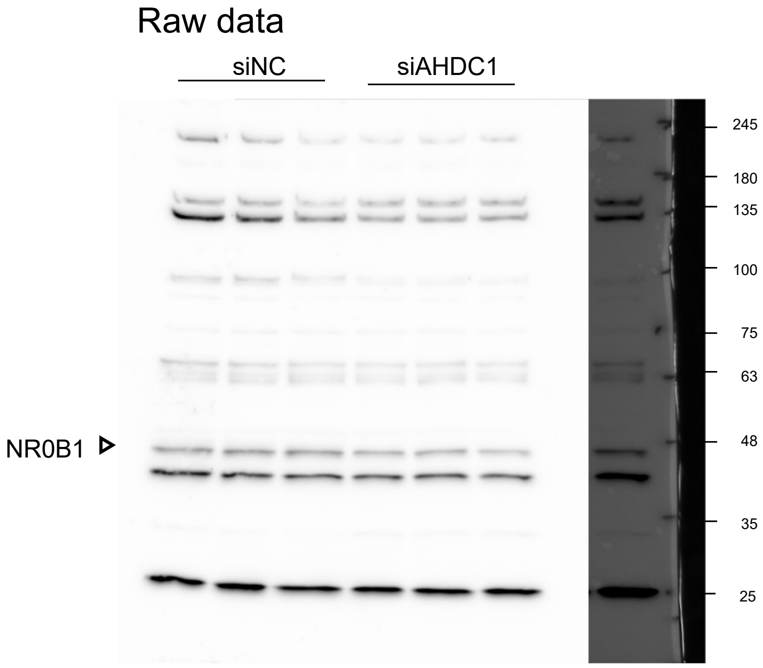

NR0B1 ab

Fig S4D

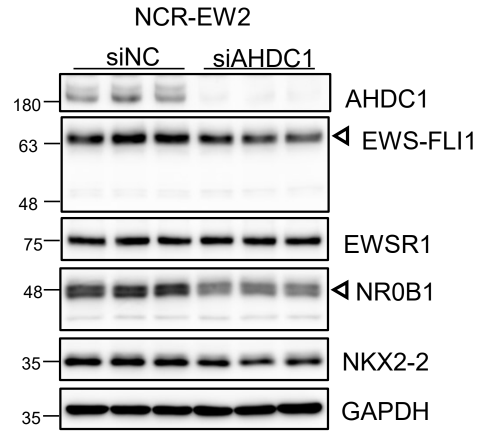

Raw data

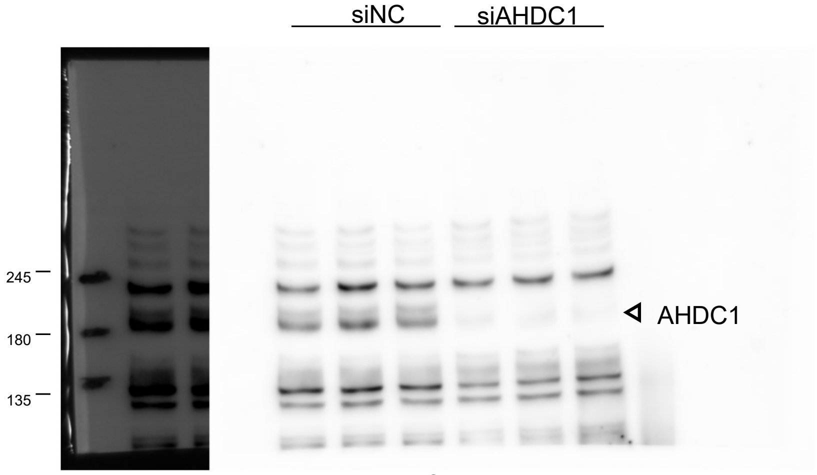

AHDC1 ab

Raw data

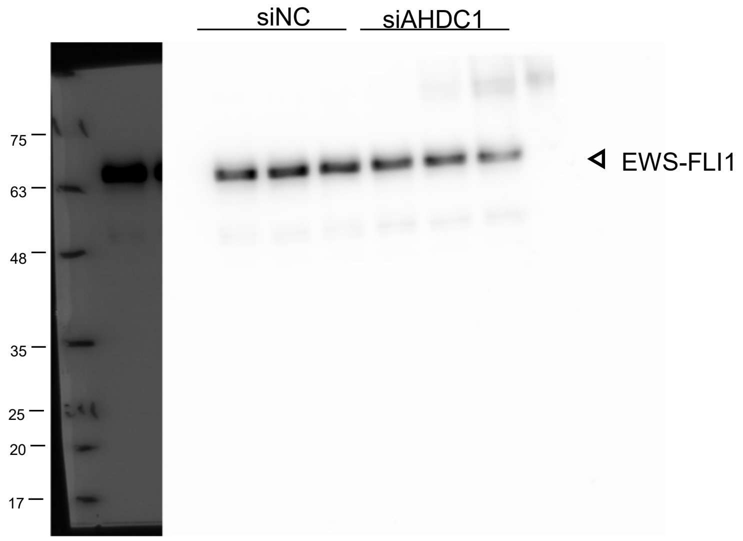

FLI1 ab

Raw data

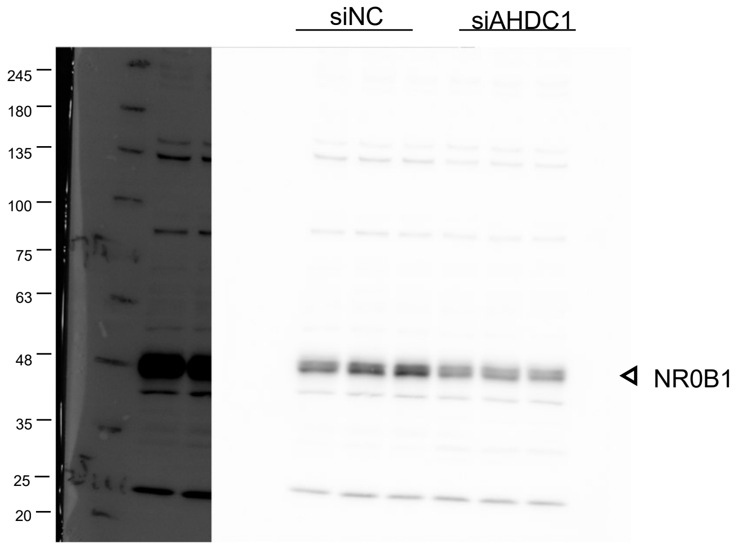

NR0B1 ab

Fig S4D

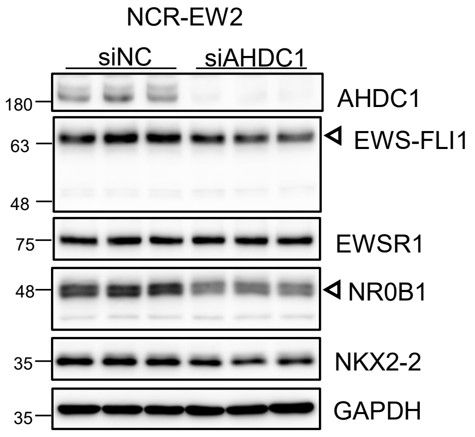

Raw data

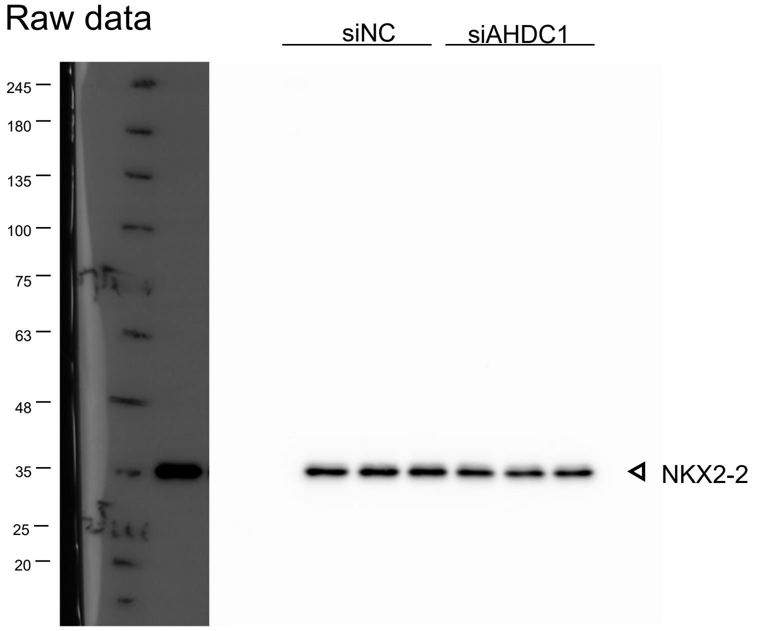

NKX2-2 ab

Raw data

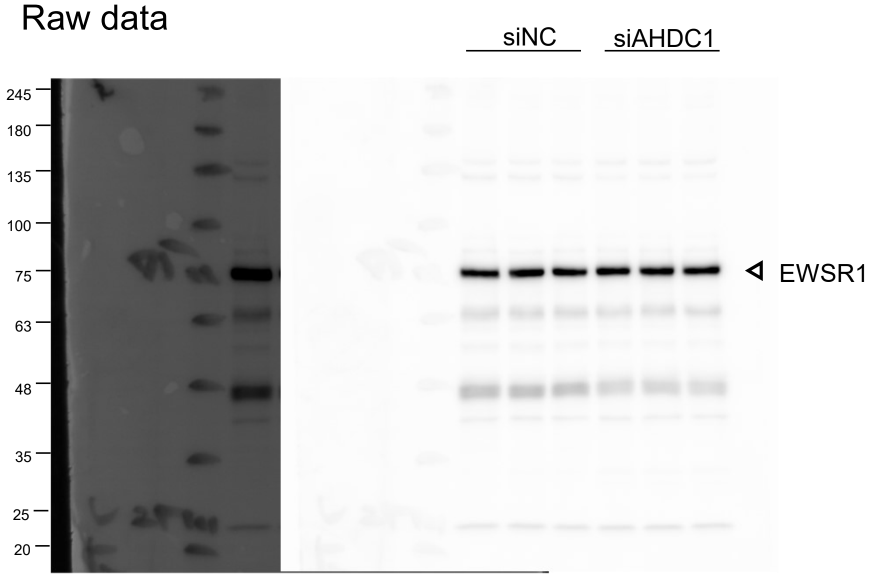

EWSR1 ab

Raw data

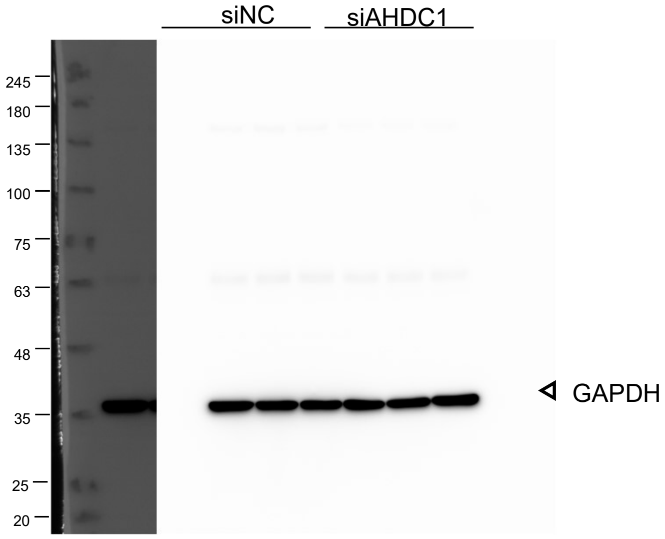

GAPDH ab

Fig S5A

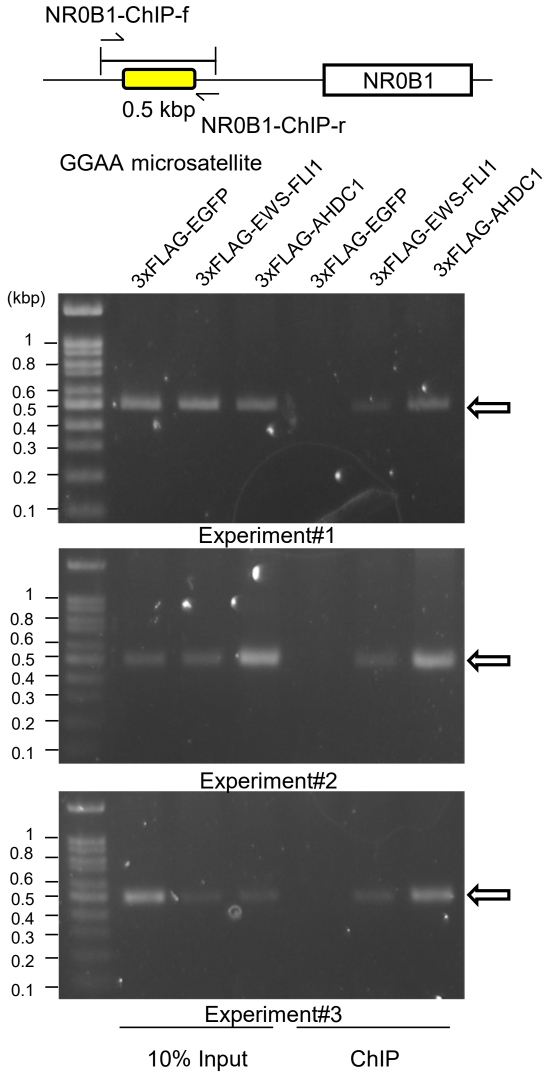

Raw data

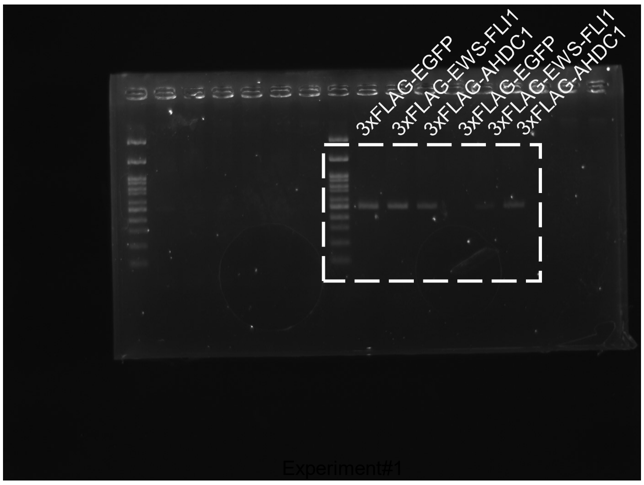

Experiment#1

Raw data

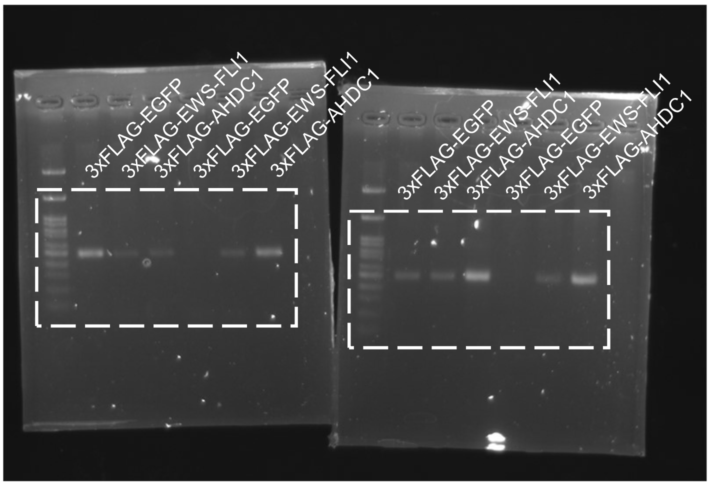

Experiment#3 Experiment#2

Fig S5B

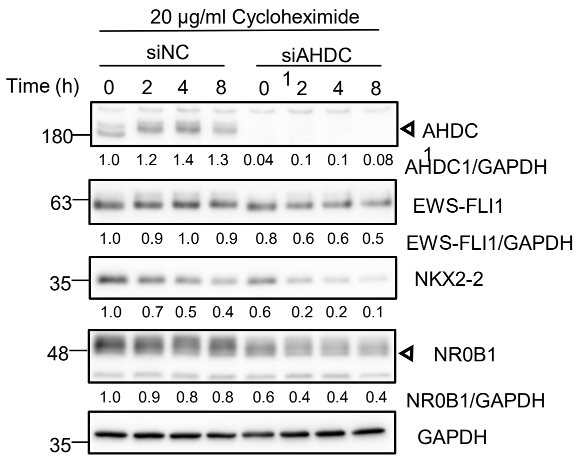

Raw data

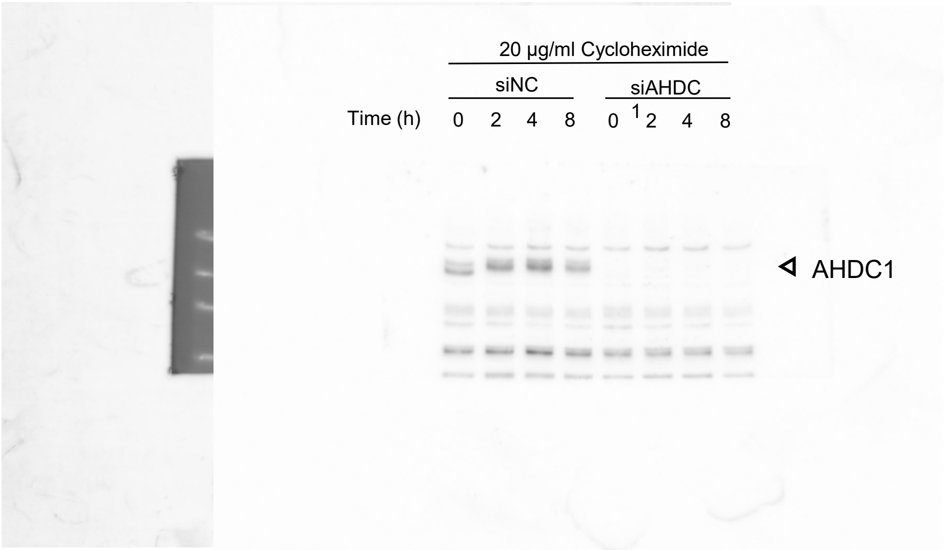

AHDC1 ab

Raw data

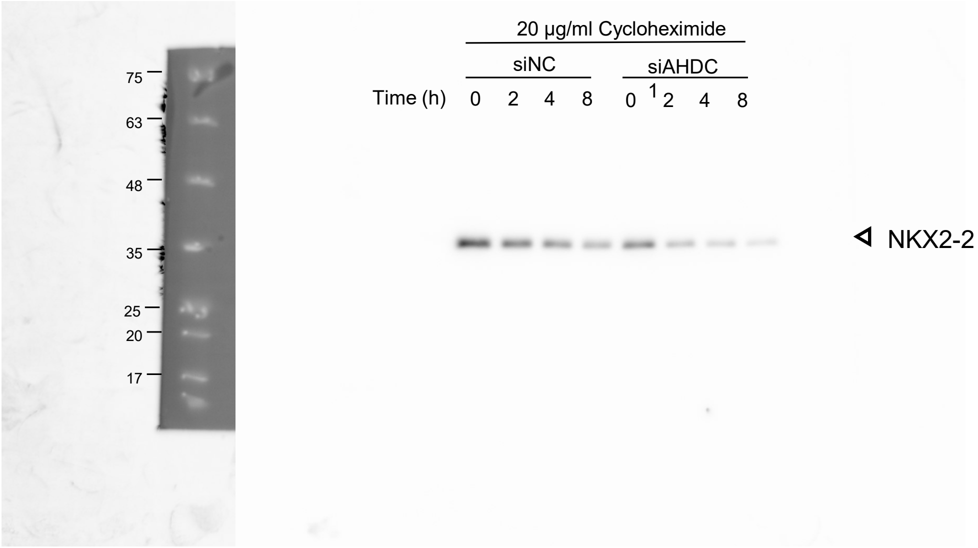

NKX2-2 ab

Fig S5B

Raw data

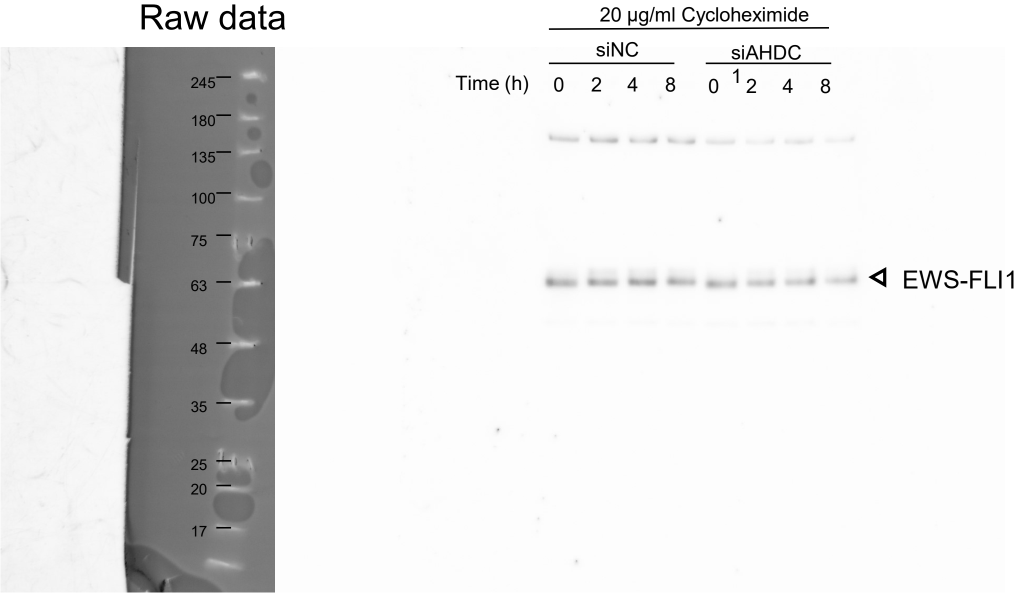

FLI1 ab

Raw data

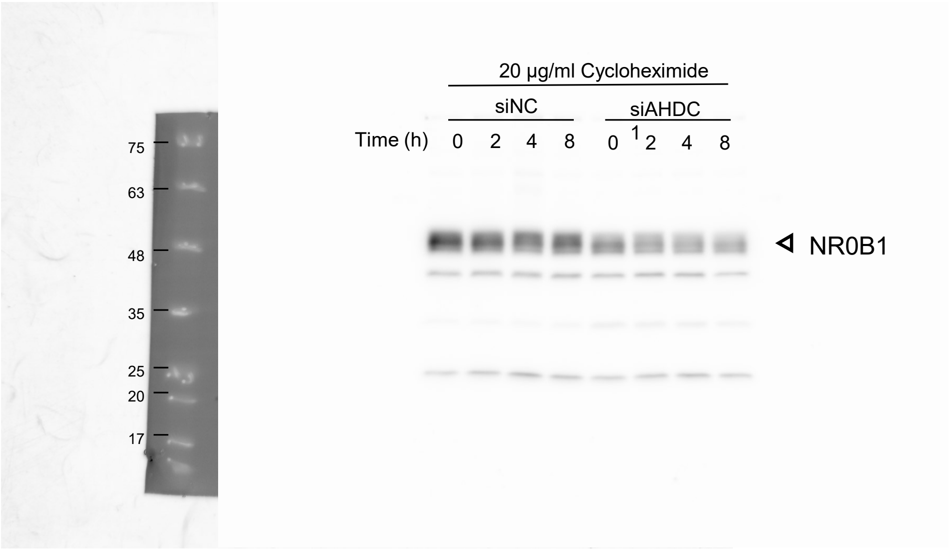

NR0B1 ab

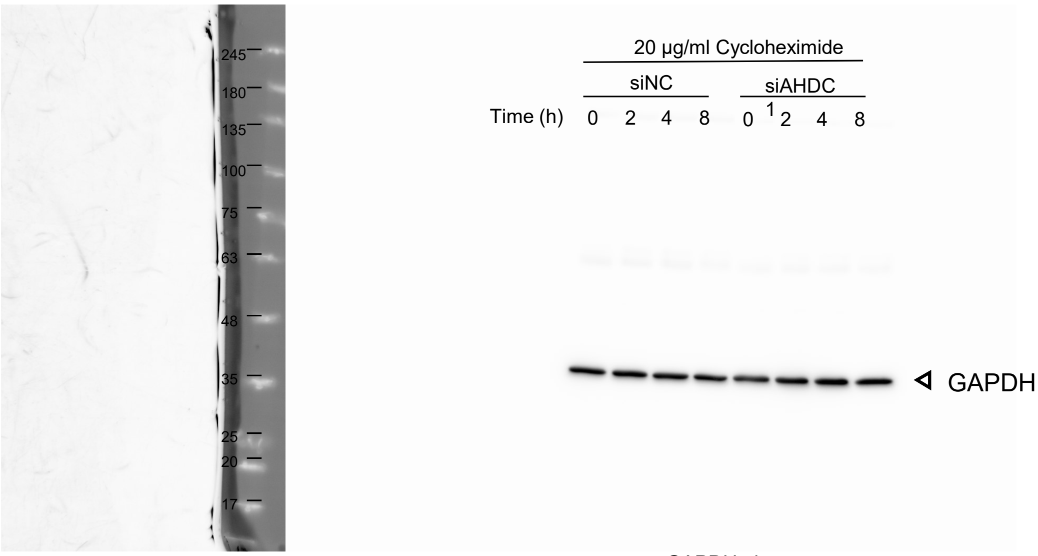

GAPDH ab

Fig S5C

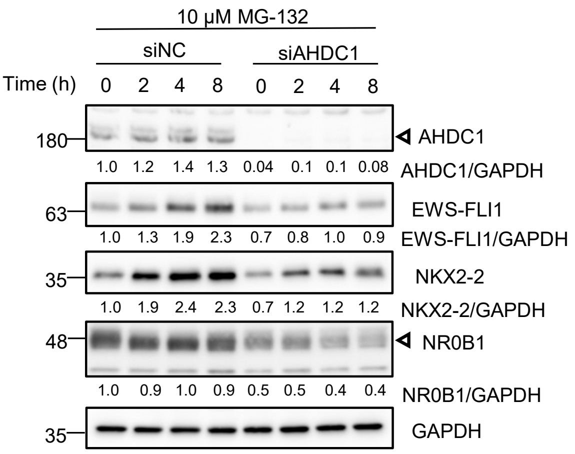

Raw data

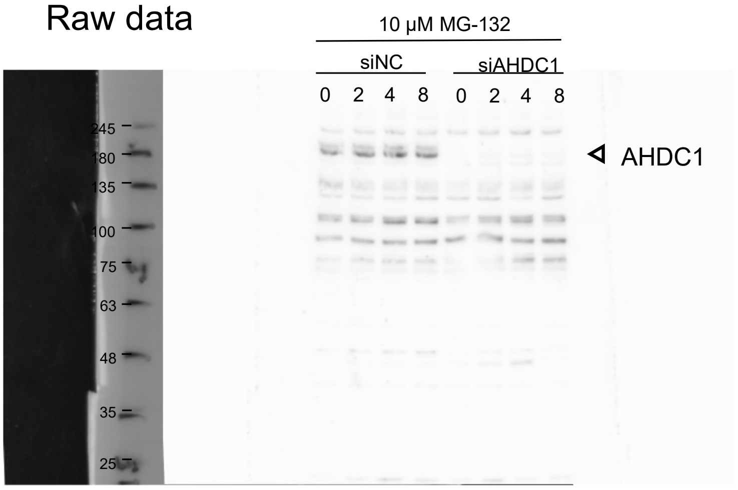

AHDC1 ab

Raw data

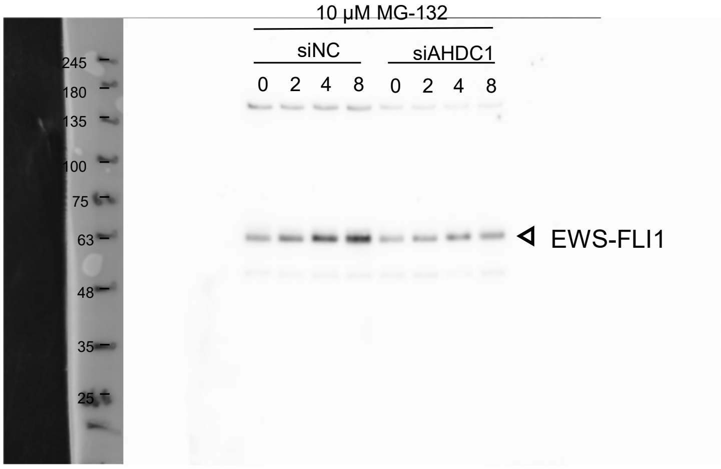

FLI1 ab

Fig S5C

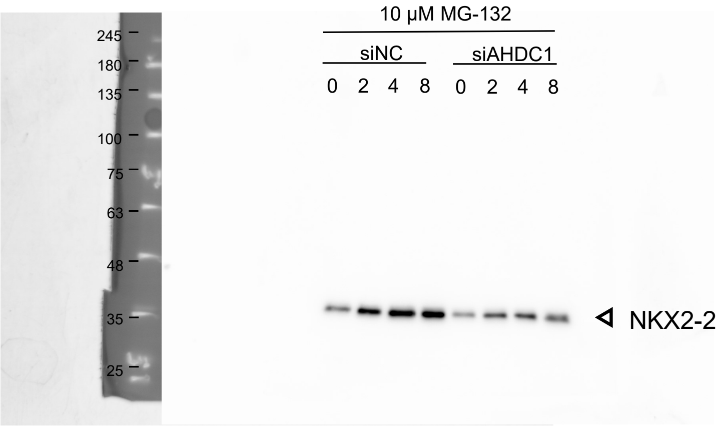

NKX2-2 ab

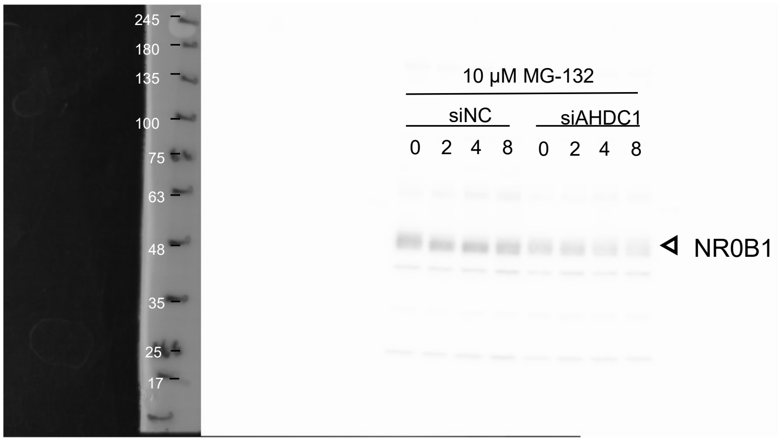

NR0B1 ab

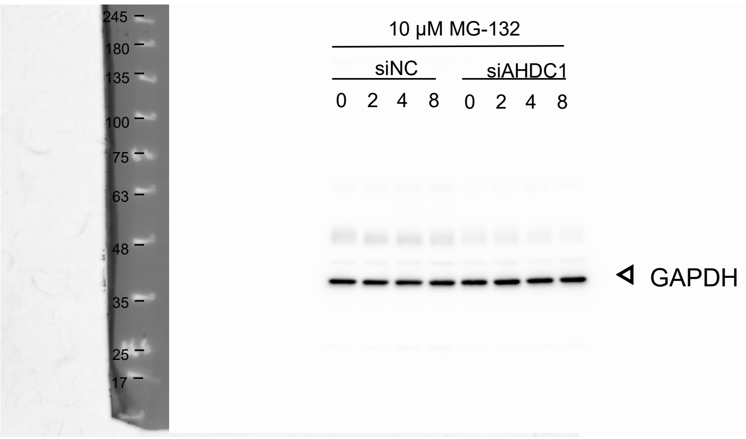

GAPDH ab

S6 Fig

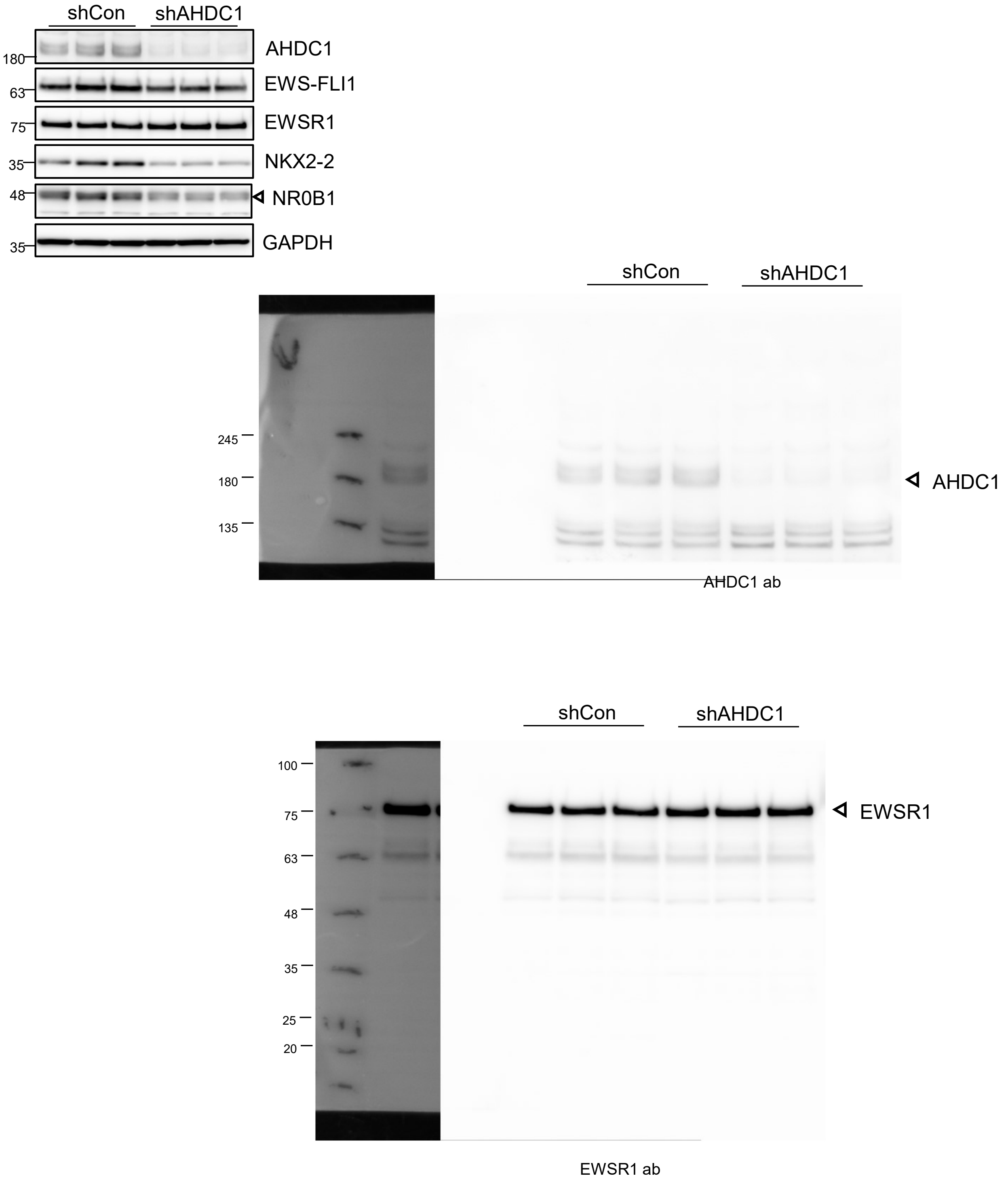
shCon
shAHDC1
180
AHDC1
63
EWS-FLI1
75
EWSR1
35
NKX2-2
48
NR0B1
35
GAPDH
shCon
shAHDC1
245
180
135
AHDC1
AHDC1 ab
shCon
shAHDC1
100
75
63
48
35
25
20
EWSR1
EWSR1 ab

Fig S6

Raw data

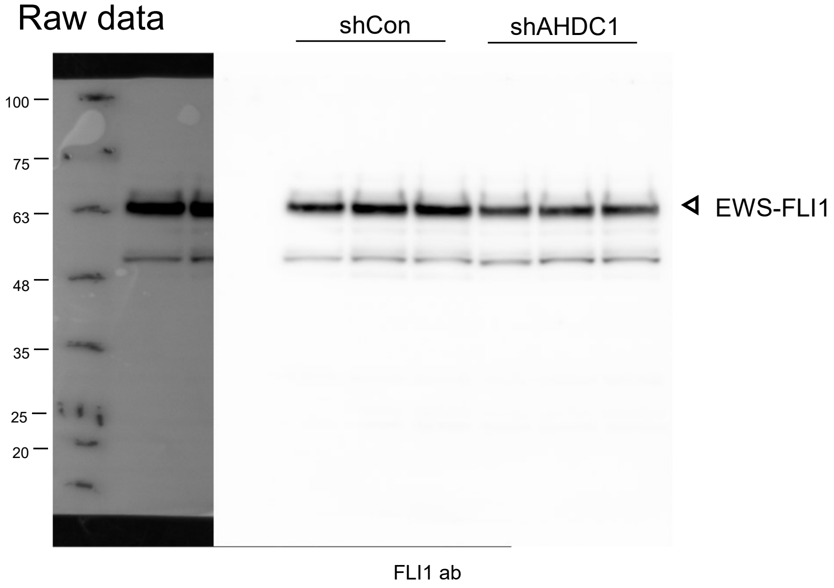

Raw data

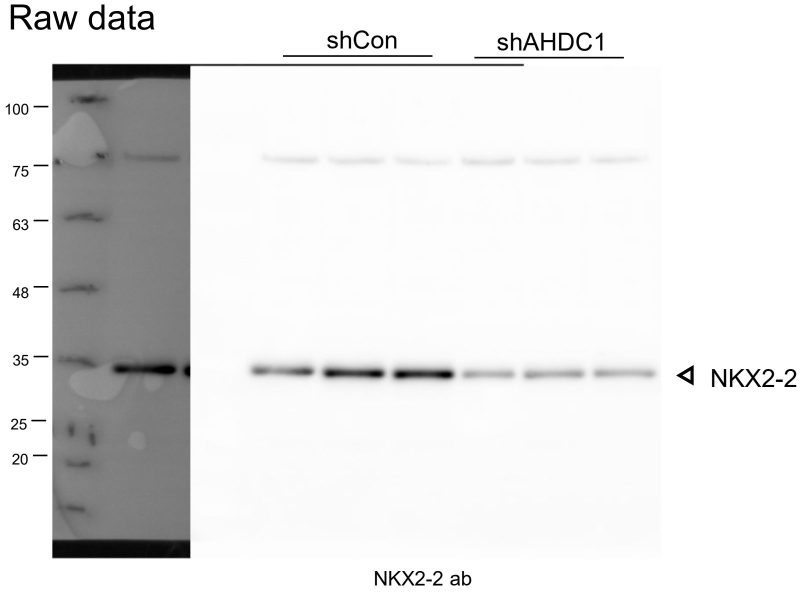

Raw data

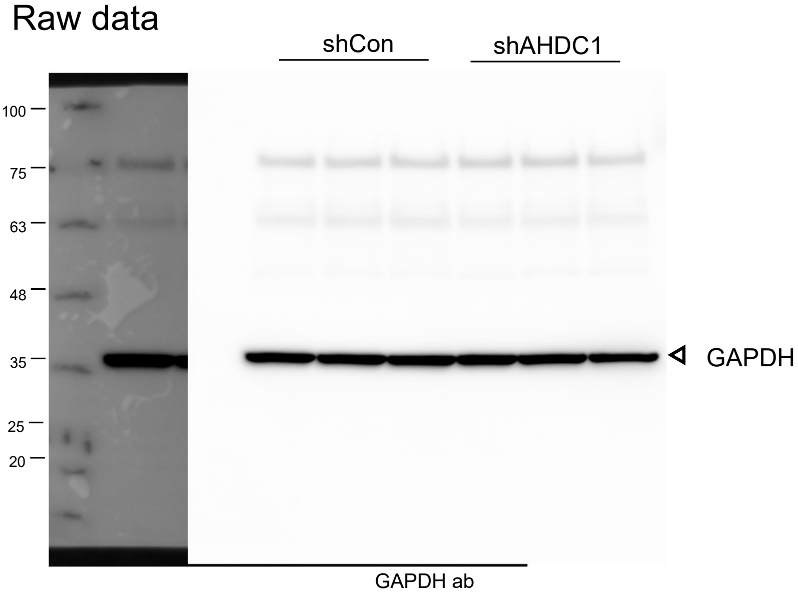

Fig S6

Raw data

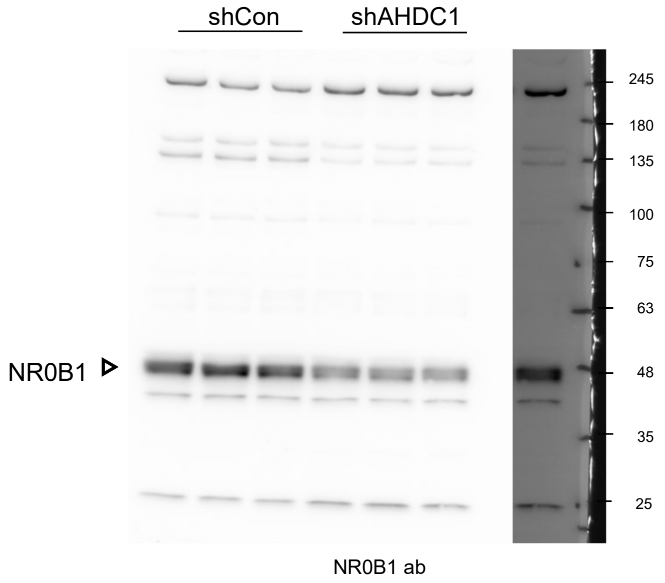

Fig S7B

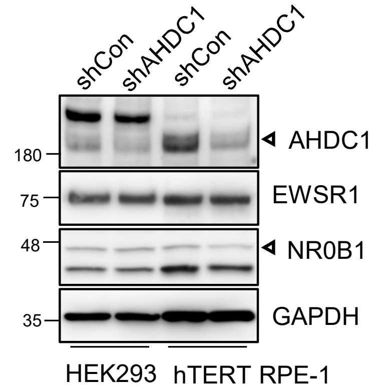

Raw data

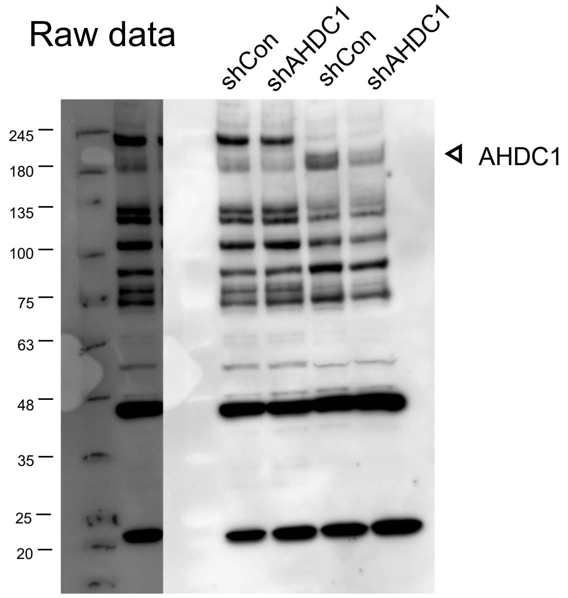

AHDC1 ab

Raw data

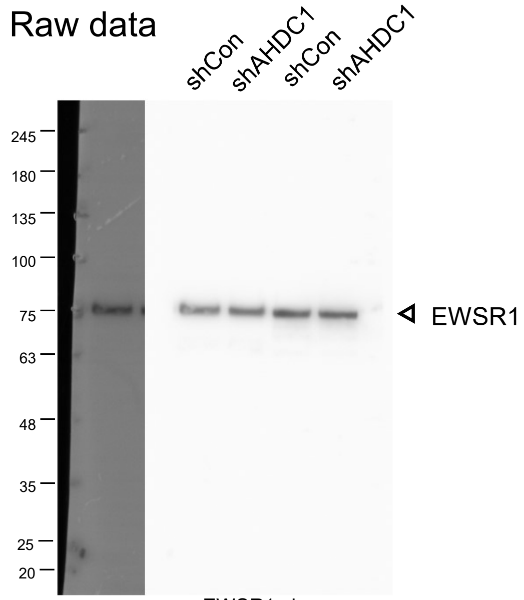

EWSR1 ab

Raw data

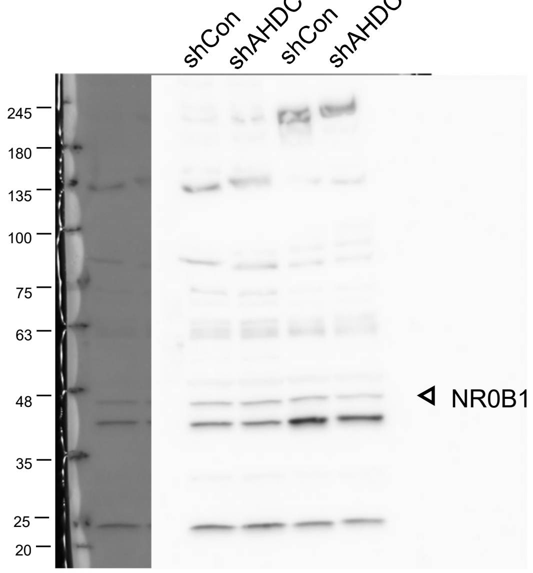

NR0B1 ab

Raw data

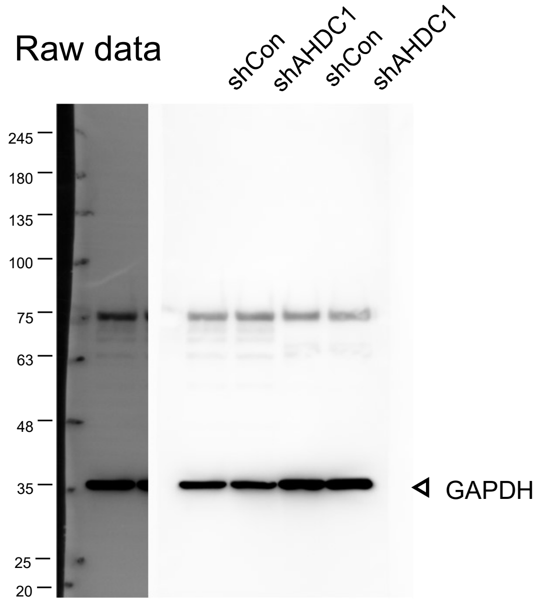

GAPDH ab

Fig S8A

SAOS-2

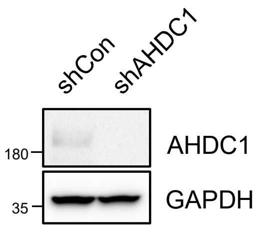

Raw data

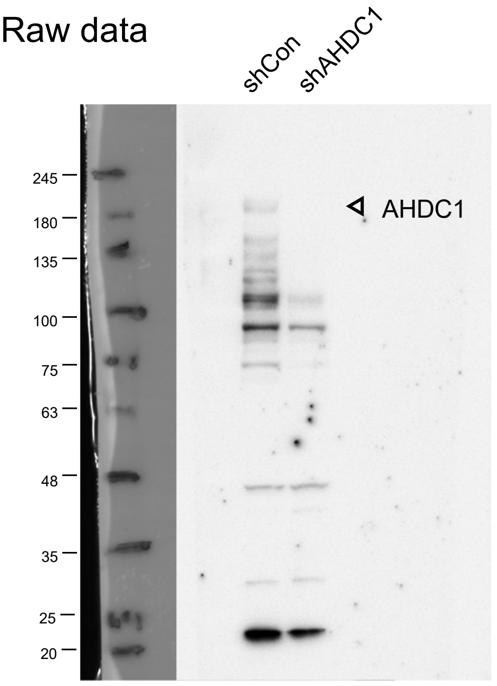

AHDC1 ab

Raw data

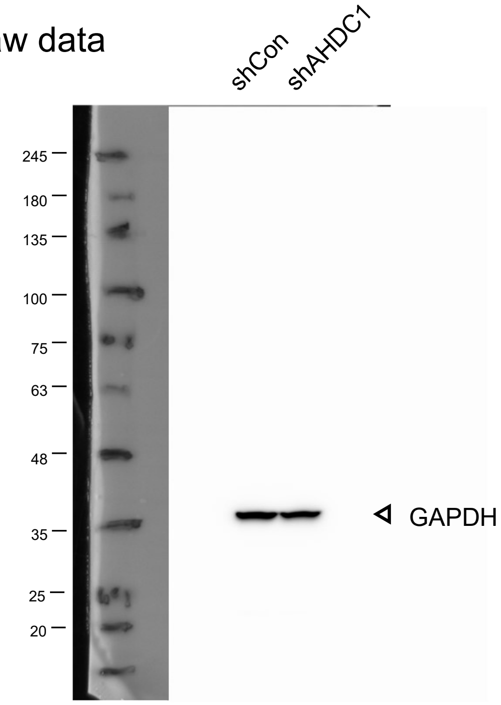

GAPDH ab

Fig S8B

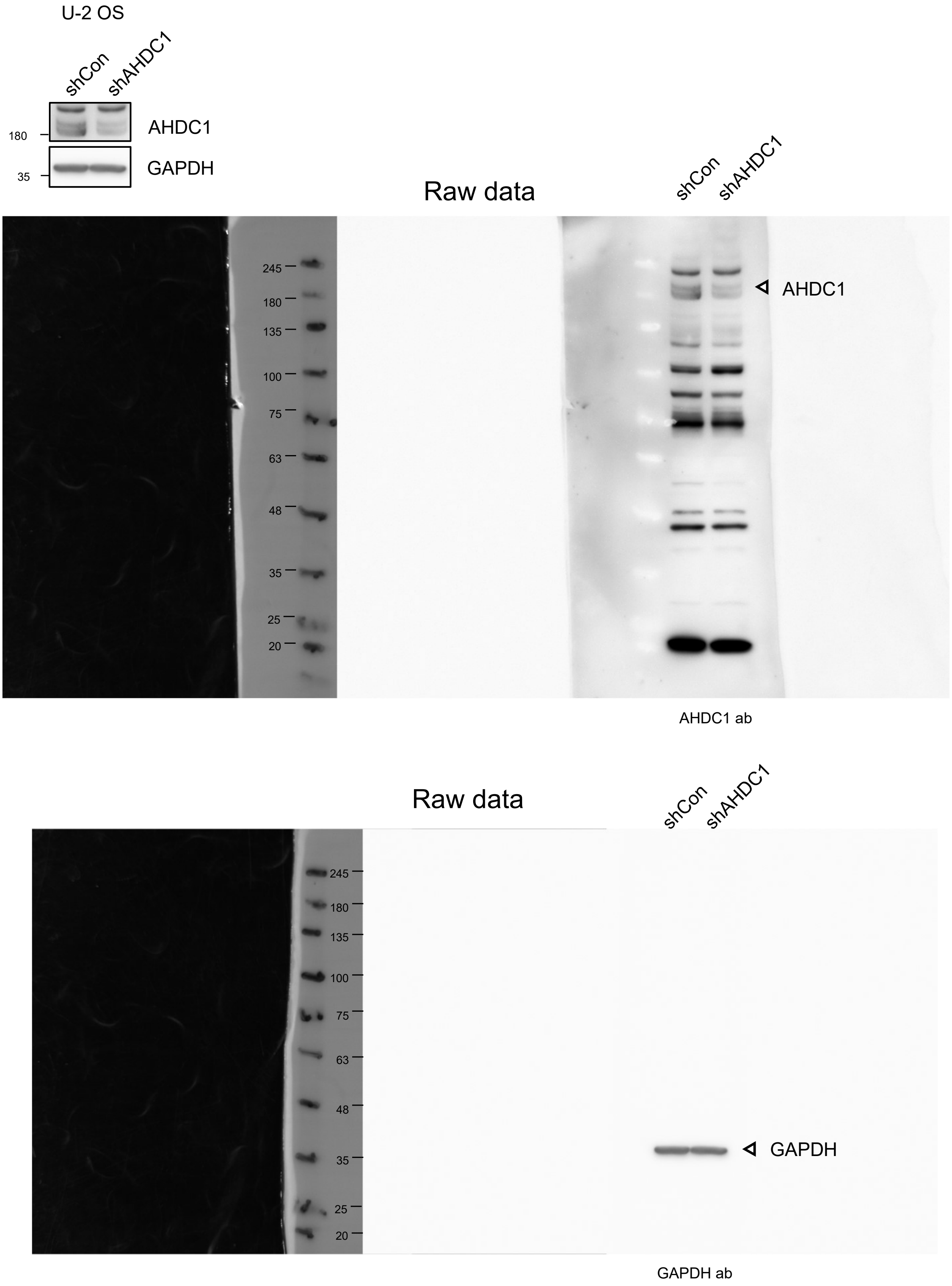

U-2 OS
shCon
shAHDC1
180
AHDC1
35
GAPDH
Raw data
shCon
shAHDC1
245
180
135
100
75
63
48
35
25
20
AHDC1
AHDC1 ab
Raw data
shCon
shAHDC1
245
180
135
100
75
63
48
35
25
20
GAPDH
GAPDH ab

Fig S9A

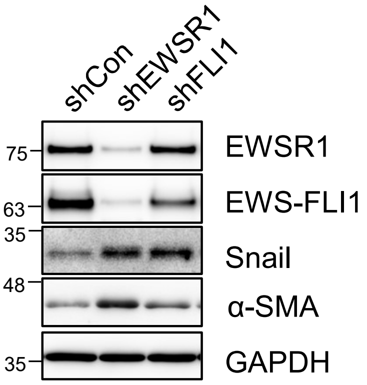

Raw data

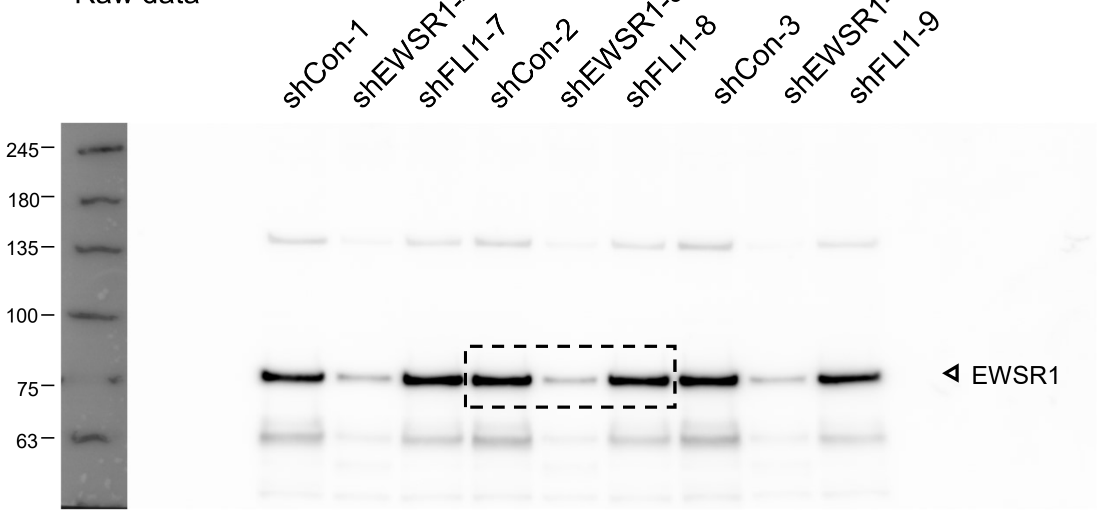

EWSR1 ab

Raw data

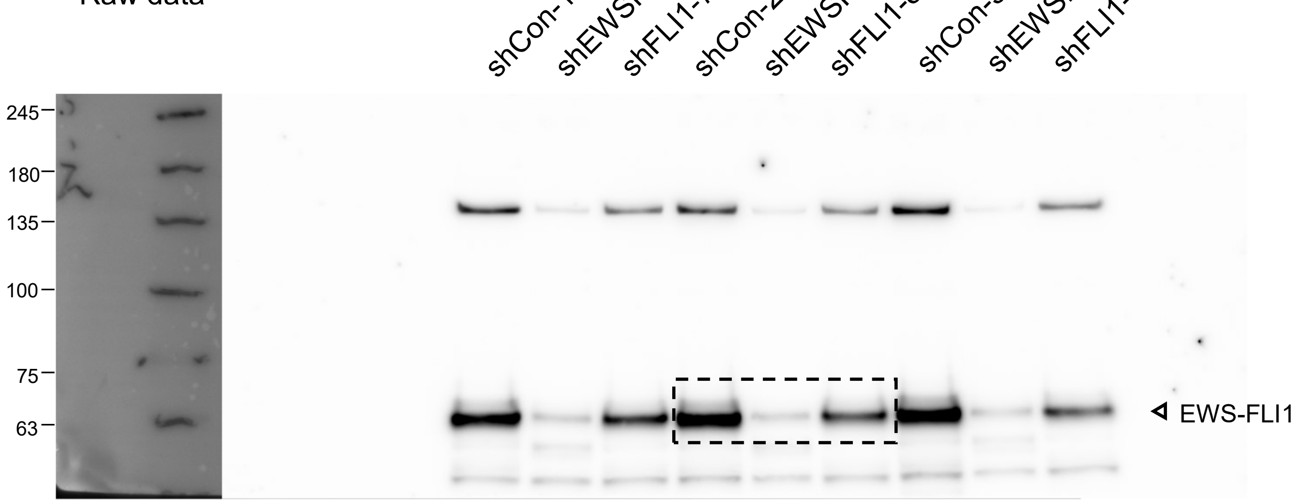

FLI1 ab

Fig S9A

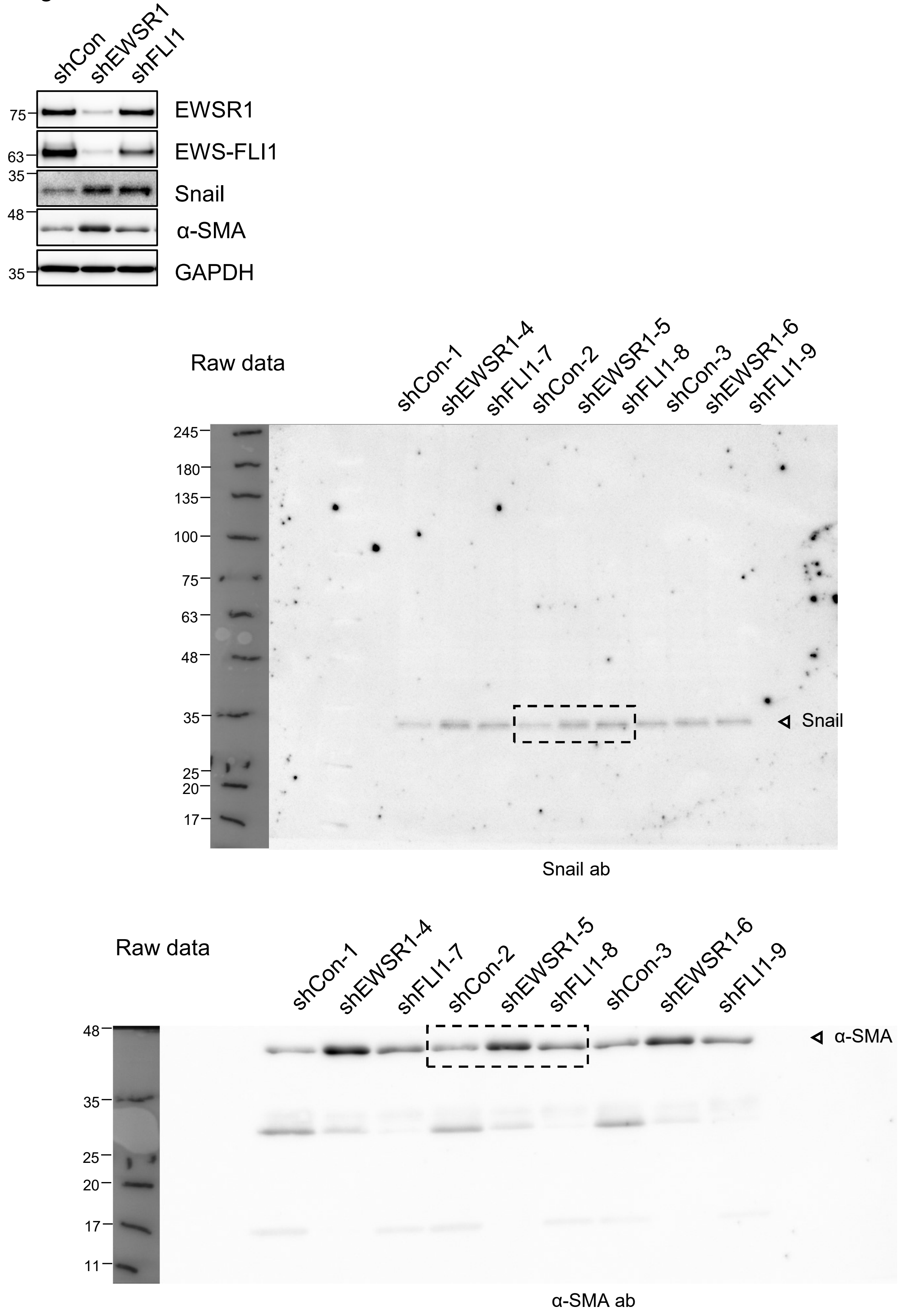
shCon
shEWSR1
shFLI1
75
EWSR1
63
EWS-FLI1
35
Snail
48
α-SMA
35
GAPDH
Raw data
shCon-1
shEWSR1-4
shFLI1-7
shCon-2
shEWSR1-5
shFLI1-8
shCon-3
shEWSR1-6
shFLI1-9
245
180
135
100
75
63
48
35
25
20
17
Snail
Snail ab
Raw data
shCon-1
shEWSR1-4
shFLI1-7
shCon-2
shEWSR1-5
shFLI1-8
shCon-3
shEWSR1-6
shFLI1-9
48
35
25
20
17
11
α-SMA
α-SMA ab

Fig S9A

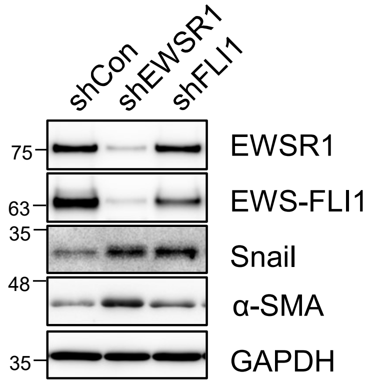

Raw data

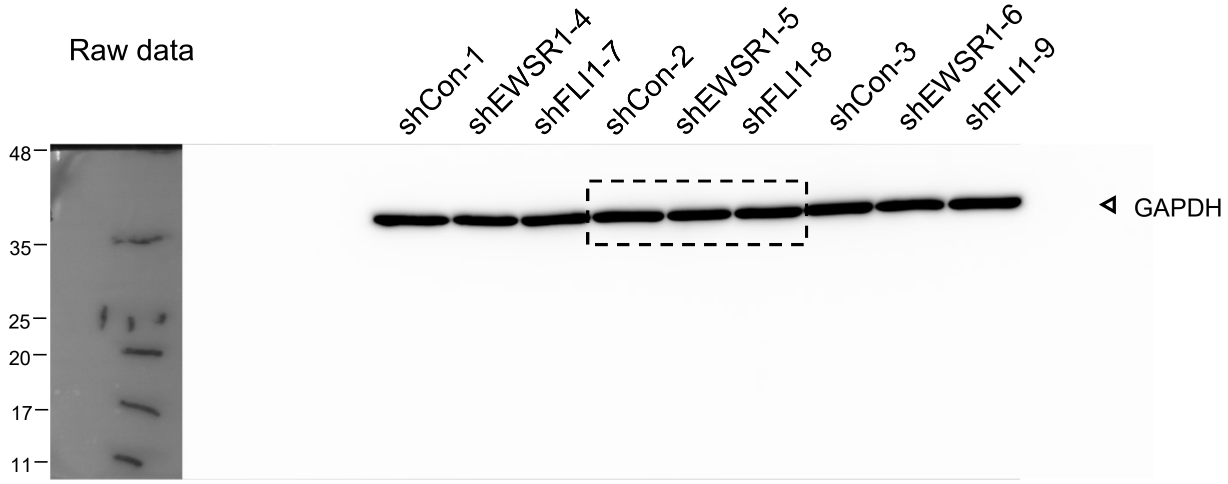

GAPDH ab
